# Highly Stereoselective Synthesis of Polycyclic Indoles through Rearrangement/[4+2] Cycloaddition under Sequential Catalysis

**DOI:** 10.1002/open.201200028

**Published:** 2012-09-11

**Authors:** Di-Han Zhang, Min Shi

**Affiliations:** a State Key Laboratory of Organometallic Chemistry, Shanghai Institute of Organic Chemistry, Chinese Academy of Sciences345 Lingling Road, Shanghai 200032 (China) E-mail: Mshi@mail.sioc.ac.cn

**Keywords:** cycloaddition reactions, homogeneous catalyses, polycyclic compounds, rearrangements, sequential catalyses

The indole moiety is a privileged structural motif in many biologically active and medicinally valuable molecules.[1] Polycyclic frameworks lead to relatively rigid structures that could be expected to show substantial selectivity in their interactions with enzymes or receptors.[2] Construction of polycyclic indoles usually requires multistep approaches.[3] The preparation of polyfunctional indoles is therefore an important research field.[4]

Sequential catalysis involving a binary catalytic system often reduces labor and waste and therefore has attracted much attention recently.[5] Homogeneous catalysis by gold complex has also received considerable attention in recent years.[6] The combination of mechanistically distinct organocatalysis and transition-metal catalysis, especially gold catalysis, has enabled novel transformations beyond those possible with single catalytic systems.[7–9] During our ongoing investigation on the nitrogen- or phosphine-containing Lewis base-catalyzed chemical transformation, we found that nitrogen-containing Lewis bases are efficient catalysts for highly regioselective and stereoselective cycloadditions of allenoates.[10, 11] Thus, we envisaged that it might be possible to explore a direct route to polycyclic indoles by means of a sequential catalysis of gold complex and a nitrogen-containing Lewis base.[12]

In 2010, Gagosz’s group reported a novel gold-catalyzed rearrangement of propargyl benzyl ethers that allows for rapid preparation of variously substituted allenes (Scheme 1 A).[13] As for isatin-derived propargyl benzyl ether **1 a**, the α,β-unsaturated ketone **2 a** could be formed in 20 % yield along with the release of HOBn (determined by GC analysis) rather than the allene product in wet dichloromethane (Scheme 1 B). Herein, we wish to report an interesting rearrangement/cycloaddition based on sequential catalysis of gold complex and a nitrogen-containing Lewis base to construct polycyclic indoles.

**Scheme 1:**
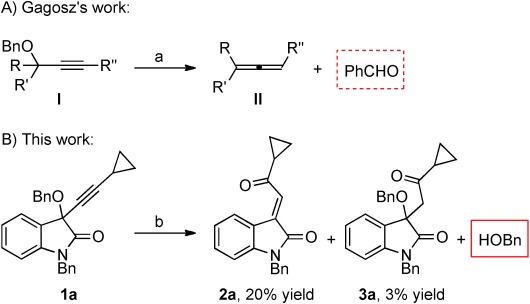
Gold-catalyzed rearrangement of propargyl benzyl ethers according to A) Gagosz et al.[13] and B) this work. *Reagents and conditions:* a) [XPhosAu(NCMe)SbF_6_] (4 mol %), CHCl_3_, 20 °C or 60 °C, 1–3 h. b) [(Ph_3_P)AuCl]/AgOTf (5 mol %), CH_2_Cl_2_ (wet), RT, 2 h.

In order to clarify the effect of water on the rearrangement of benzyl ether **1 a**, we first carried out the reaction in freshly distilled dichloromethane containing various concentrations of water. The results are summarized in Table 1, and as can be seen the concentration of water has an obvious effect on this reaction: 1.0 equiv of water is enough to give **2 a** in good yield.

**Table 1:** Effect of different water concentrations for the gold(I)-catalyzed rearrangement[a]

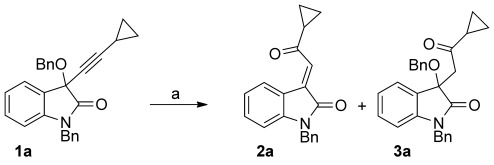		

H_2_O [equiv]	Yield 2 a[%]	Yield 3 a[%]
0.5	30	5
1.0	41	5
1.5	36	20
2.0	33	35

[a] *Reagents and conditions:* a) [(Ph_3_P)AuCl]/AgOTf (5 mol %), CH_2_Cl_2_, RT, 2–10 h.

Next, we used propargyl benzyl ether **1 a** (0.1 mmol) as the substrate to optimize the reaction conditions. The results are summarized in Table 2. Examination of solvent effects revealed that chloroform was the solvent of choice giving **2 a** in 67 % yield, whereas, in other organic solvents such as 1,2-dichloroethane, toluene, acetonitrile or 1,4-dioxane, **2 a** was formed in lower yield (Table 2, Entries 1–5). Carrying out the reaction in the presence of [(*t*Bu_3_P)AuCl] or [(Me_3_P)AuCl] (5 mol %) afforded the desired product **2 a** in 40 % and 52 % yields, respectively (Table 2, Entries 6 and 7). Using [AuCl] or [AuCl_3_] instead of [(Ph_3_P)AuCl] as the gold catalyst gave **2 a** in 46 % and 42 % yields, respectively, and [Ph_3_PAu]_3_OBF_4_ as well as [(*t*BuXPhos)Au(NCMe)]SbF_6_ were not effective gold catalysts in this reaction (Table 2, Entries 8–11). Changing silver salt to AgSbF_6_ or AgBF_4_ did not improve the reaction outcomes (Table 2, Entries 12 and 13). Moreover, adding [(Ph_3_P)AuCl]/AgOTf (10 mol %) afforded **2 a** in 52 % yield (Table 2, Entry 14). Control experiments indicated that using [(Ph_3_P)AuCl] or AgOTf alone as the catalyst did not promote the reaction (Table 2, Entries 15 and 16). Therefore, optimal reaction conditions were found when the reactions were carried out in chloroform at room temperature using [(Ph_3_P)AuCl]/AgOTf (5 mol %) as the catalyst in the presence of water (1.0 equiv).

**Table 2:** Optimization of the reaction conditions for the gold(I)-catalyzed rearrangement[a,b]

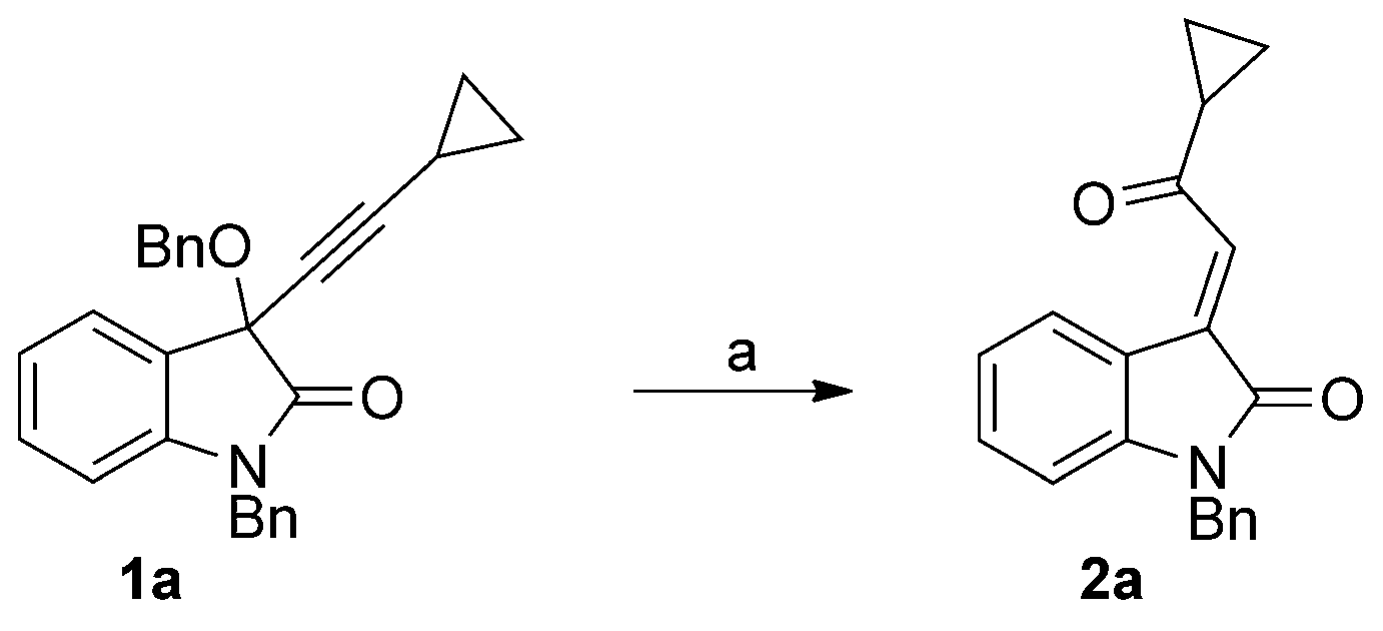				

Entry	Catalyst	Solvent	*t* [h]	Yield [%][c]
1	[(Ph_3_P)AuCl]/AgOTf	DCE	2	21
2	[(Ph_3_P)AuCl]/AgOTf	Toluene	2	37
3	[(Ph_3_P)AuCl]/AgOTf	CH_3_CN	10	NR
4	[(Ph_3_P)AuCl]/AgOTf	1,4-Dioxane	15	41
5	[(Ph_3_P)AuCl]/AgOTf	CHCl_3_	2	67
6	[(*t*Bu_3_P)AuCl]/AgOTf	CHCl_3_	2	40
7	[(Me_3_P)AuCl]/AgOTf	CHCl_3_	2	52
8	[AuCl]/AgOTf	CHCl_3_	2	46
9	[AuCl_3_]/AgOTf	CHCl_3_	2	42
10	[Ph_3_PAu]_3_OBF_4_	CHCl_3_	2	24
11	[(*t*BuXPhos)Au(NCMe)]SbF	CHCl_3_	15	8
12	[(Ph_3_P)AuCl]/AgSbF_6_	CHCl_3_	2	38
13	[(Ph_3_P)AuCl]/AgBF_4_	CHCl_3_	2	27
14	[(Ph_3_P)AuCl]/AgOTf[d]	CHCl_3_	1	52
15	AgOTf	CH_2_Cl_2_	10	NR
16	[(Ph_3_P)AuCl]	CHCl_3_	10	NR

[a] *Reagents and conditions:* a) **1 a** (0.1 mmol), H_2_O (1.0 equiv), catalyst (5 mol %), solvent (2.0 mL), RT, unless otherwise specified.

[b] 10–20 % of benzyl ether **3 a** was formed in the reaction.

[c] Yield of isolated product.

[d] 10 mol % calalyst was used. NR=no reaction; Bn=benzyl; DCE=1,2-dichloroethane.

We subsequently examined the substrate scope of the reaction catalyzed by gold under the optimized conditions, and the results are shown in Table 3. As can be seen, as for *N*-Bn protected substrates **1 b**–**1 d** having an alkyl group at the terminus of the alkyne moiety (R^1^), α,β-unsaturated ketones **2 b**–**2 d** could be afforded in 45–50 % yields (Table 3, Entries 1–3). Regardless of whether electron-withdrawing or electron-donating groups at the 5-, 6- or 7-position of the benzene ring of *N*-Bn protected isatins **1 e**–**1 o** were employed, the reactions proceeded smoothly to give the corresponding products **2 e**–**2 o** in moderate yields (up to 61 % yield; Table 3, Entries 4–14). In the case of other substrates **1 p**–**1 s** bearing different *N*-protecting groups, the reaction also produced the desired products **2 p**–**2 s** in 34–55 % yields (Table 3, Entries 15–18). It should be mentioned here that 10–25 % of benzyl ether **3** were formed in all cases. Moreover, as for propargylic acetate **1 t**, the corresponding enone **2 a** was afforded only in 15 % yield under the standard conditions (Scheme 2). The structure of compound **2 i** was confirmed by NMR spectroscopy and X-ray crystal structure analysis.[14] The ORTEP drawing of **2 i** is shown in Figure 1. The structures of products **2 b**–**2 s** were determined by NMR, MS, and HRMS (for details, see the Supporting Information).

**Table 3:** Substrate scope of the gold(I)-catalyzed rearrangement[a,b]

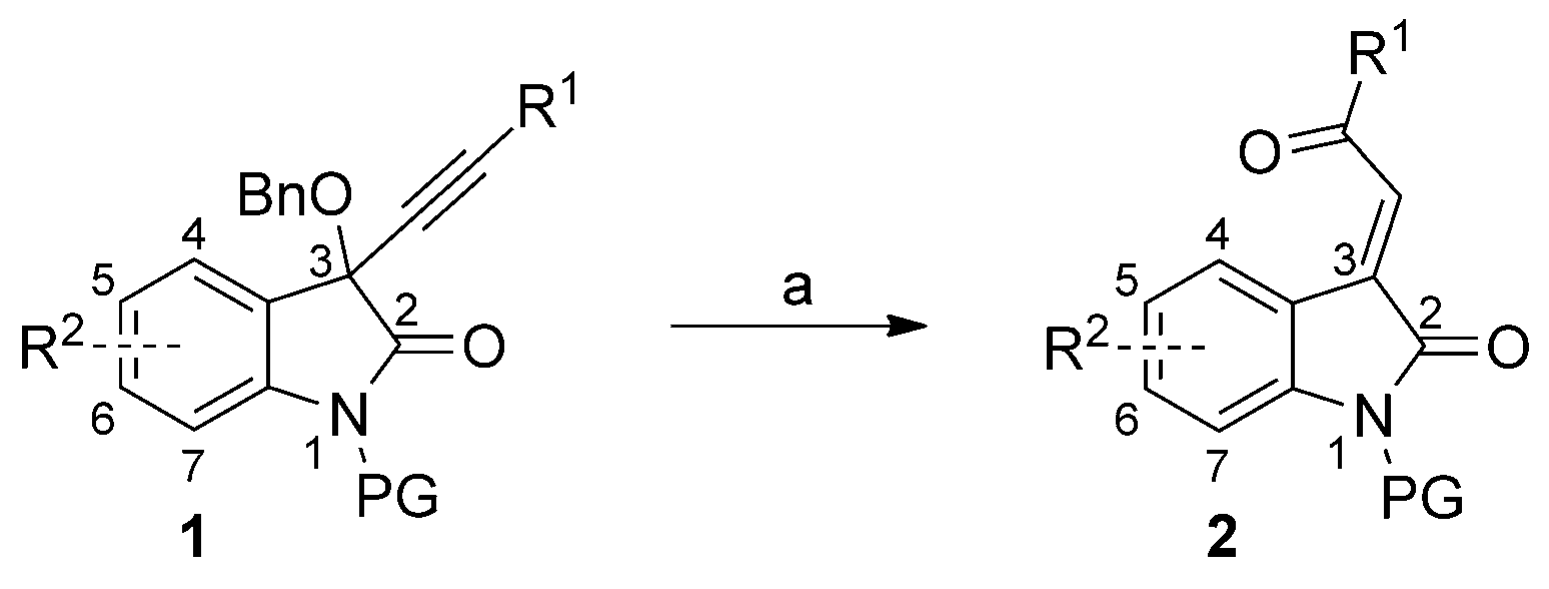						

Entry	Compd	R^1^	R^2^	PG	Product	Yield [%][c]
1	**1 b**	Cyclohexyl	H	Bn	**2 b**	50
2	**1 c**	Me	H	Bn	**2 c**	45
3	**1 d**	*n*Bu	H	Bn	**2 d**	46
4	**1 e**	Cyclopropyl	5-Br	Bn	**2 e**	60
5	**1 f**	Cyclopropyl	5-Cl	Bn	**2 f**	57
6	**1 g**	Cyclopropyl	5-F	Bn	**2 g**	48
7	**1 h**	Cyclopropyl	5-Me	Bn	**2 h**	58
8	**1 i**	Cyclopropyl	5-MeO	Bn	**2 i**	61
9	**1 j**	Cyclopropyl	6-Br	Bn	**2 j**	58
10	**1 k**	Cyclopropyl	6-Cl	Bn	**2 k**	53
11	**1 l**	Cyclopropyl	6-Me	Bn	**2 l**	59
12	**1 m**	Cyclopropyl	7-Br	Bn	**2 m**	47
13	**1 n**	Cyclopropyl	7-Cl	Bn	**2 n**	44
14	**1 o**	Cyclopropyl	7-F	Bn	**2 o**	45
15	**1 p**	Cyclopropyl	H	Allyl	**2 p**	52
16	**1 q**	Cyclopropyl	H	Anthracen-9-ylmethyl	**2 q**	34
17	**1 r**	Cyclopropyl	H	Me	**2 r**	55
18	**1 s**	Cyclopropyl	5-Br	CPh_3_	**2 s**	39

[a] *Reagents and conditions:* a) **1** (0.2 mmol), H_2_O (1.0 equiv), [(Ph_3_P)AuCl]/AgOTf (5 mol %), CHCl_3_ (2.0 mL), RT, 3–10 h.

[b] 10–25 % benzyl ether **3** was formed during the reaction.

[c] Yield of isolated product. PG=protecting group; Bn=benzyl.

**Scheme 2:**
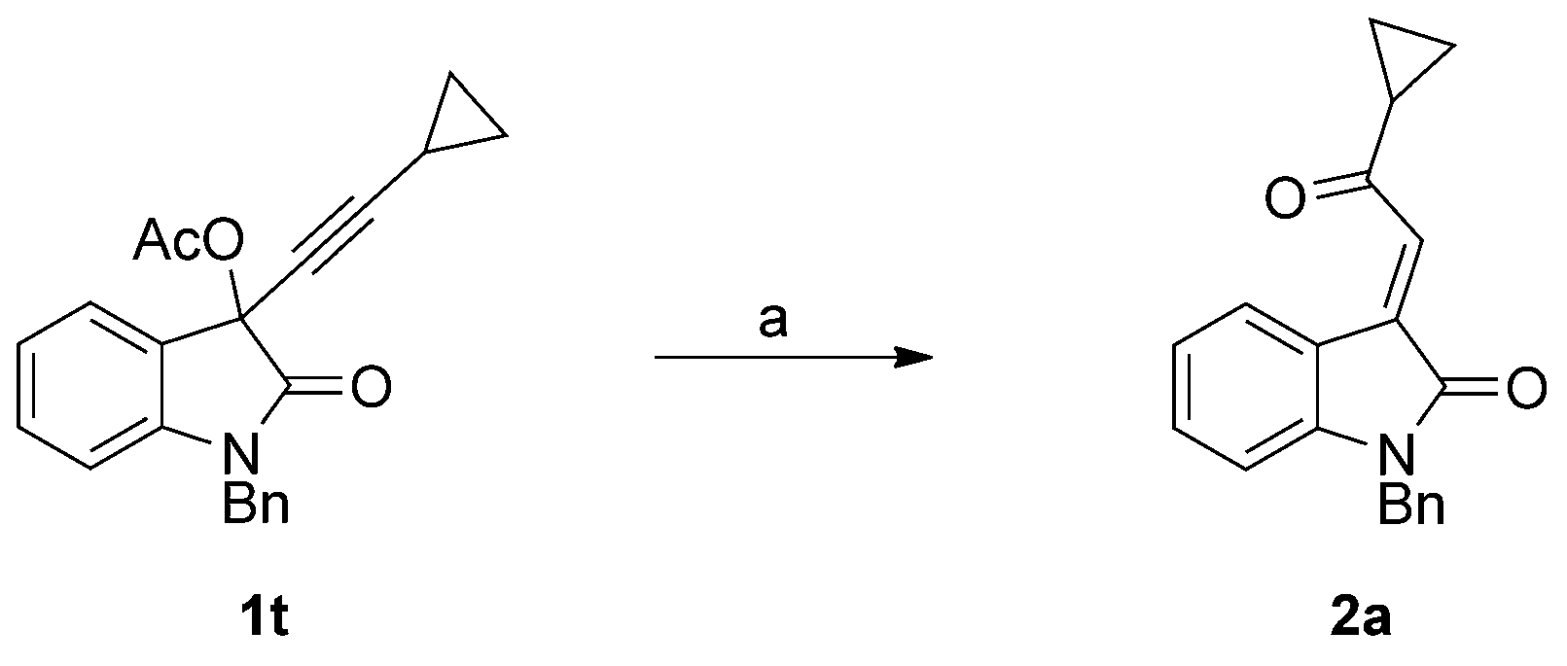
Progargylic acetate 1 t. *Reagents and conditions:* a) [(Ph_3_P)AuCl]/AgOTf (5 mol %), H_2_O (1.0 equiv), CHCl_3_, RT, 2 h, 15 %.

**Figure 1:**
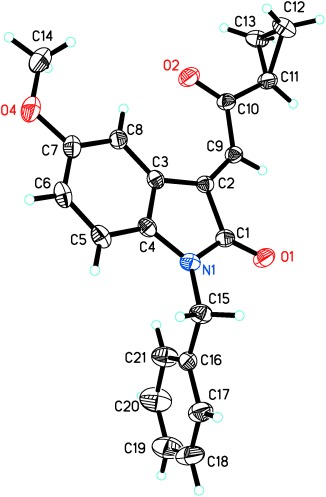
ORTEP drawing of 2 i.

Next, we utilized α,β-unsaturated ketone **2 a** (0.1 mmol) and ethyl 2,3-butadienoate **4 a** (1.5 equiv) as the substrates to investigate their cyclization behavior in the presence of nitrogen-containing Lewis bases. The results are summarized in Table 4. We found that an interesting dihydropyran derivative (**5 a**) was formed in 80 % yield using 1,4-diazabicyclo[2.2.2]octane (DABCO; 20 mol %) as the catalyst in chloroform at room temperature for 10 h (Table 4, Entry 1). Examination of solvent effects revealed that tetrahydrofuran was the solvent of choice giving **5 a** in 83 % yield, while in other organic solvents such as acetonitrile, diethyl ether, 1,4-dioxane or toluene, **5 a** was afforded in lower yields (Table 4, Entries 2–6). Using 4-*N*,*N*-dimethylpyridine (DMAP), 1,8-diazabicyclo[5.4.0]-7-undecene (DBU) or triethylamine instead of DABCO as the catalyst did not give **5 a** under otherwise identical conditions (Table 4, Entries 7–9). In the presence of K_2_CO_3_ or triphenylphosphane, **5 a** could not be obtained (Table 4, Entries 10 and 11). Increasing the employed amount of **4 a** to 2.0 equiv gave **5 a** in 86 % yield (Table 4, Entry 12).

**Table 4:** Optimization of the reaction conditions for [4+2] cycloaddition[a]

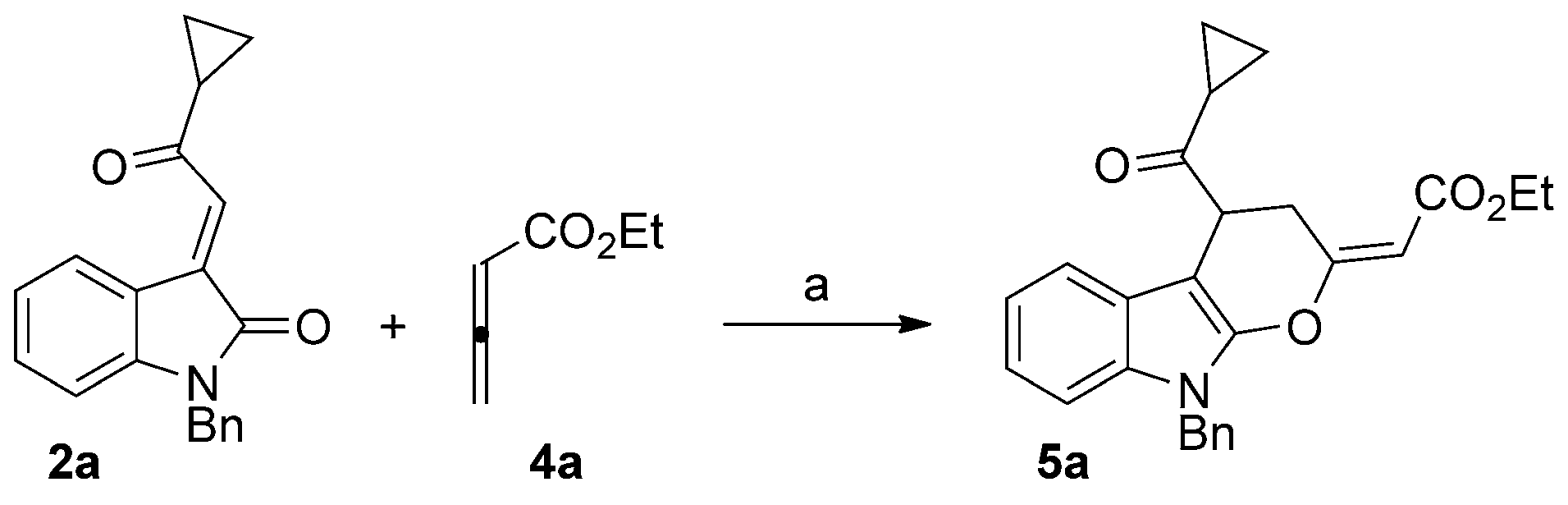				

Entry	Catalyst	Solvent	*t* [h]	Yield [%][b]
1	DABCO	CHCl_3_	10	80
2	DABCO	CH_3_CN	8	71
3	DABCO	THF	2	83
4	DABCO	Et_2_O	8	71
5	DABCO	1,4-Dioxane	10	49
6	DABCO	Toluene	8	77
7	DMAP	THF	3	complex
8	DBU	THF	3	complex
9	Et_3_N	THF	10	NR
10	K_2_CO_3_	THF	10	NR
11	PPh_3_	THF	10	0
12[c]	DABCO	THF	2	86

[a] *Reagents and conditions:* a) **2 a** (0.1 mmol), **4 a** (1.5 equiv), catalyst (20 mol %), solvent (2.0 mL), RT.

[b] Yield of isolated product.

[c] 2.0 equiv of **4 a** was added. Bn=benzyl; THF=tetrahydrofuran.

Having identified the optimal reaction conditions, we next set out to examine the scope and limitations of the [4+2] cycloaddition reaction catalyzed by DABCO. As shown in Table 5, as for *N*-Bn protected substrates **2 b**–**2 d** in which R^1^ was an alkyl group, polycyclic indoles **5 b**–**5 d** could be afforded in 70–83 % yields (Table 5, Entries 1–3). Regardless of whether electron-withdrawing or electron-donating groups at the 5-, 6- or 7-position of the benzene ring of *N*-Bn protected isatins **2 e**–**2 o** were employed, the corresponding products **5 e**–**5 o** could be formed in 63–85 % yield (Table 5, Entries 4–14). In the case of other α,β-unsaturated ketones **2 p**–**2 s** bearing different *N*-protecting groups, the reaction also proceeded smoothly to give the desired cycloadducts **5 p**–**5 s** in 74–89 % yields (Table 5, Entries 15–18). Employing α-allenic ester **4 b** (R^3^=Bn) instead of **4 a** gave corresponding polycyclic indoles **5 t** and **5 u** in 82 % and 86 % yields, respectively (Table 5, Entries 19 and 20). Further examination of **4 c** (R^3^=*t*Bu) revealed that dihydropyran derivative **5 v** could be obtained in 49 % yield at reflux temperature, and 43 % of **2 a** was recovered, indicating a broad substrate scope of this reaction (Table 5, Entry 21). The structure of compound **5 f** was confirmed by NMR spectroscopy and X-ray crystal structure analysis.[15] The ORTEP drawing of **5 f** is shown in Figure 2. The structures of products **5 b**–**5 v** were determined by NMR, MS, and HRMS (for details, see the Supporting Information).

**Table 5:** Substrate scope of the DABCO-catalyzed [4+2] cycloaddition[a]

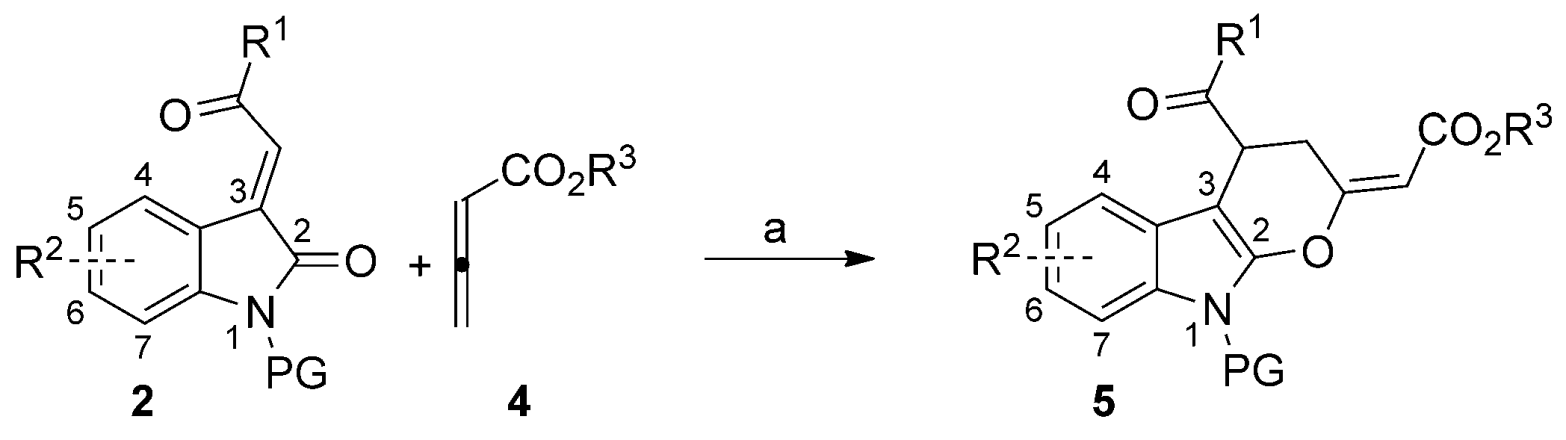								

Entry	Compd	R^1^	R^2^	PG	R^3^	*t* [h]	Product	Yield [%][b]
1	**2 b**	Cyclohexyl	H	Bn	Et (**4 a**)	1.5	**5 b**	83
2	**2 c**	Me	H	Bn	Et (**4 a**)	1.5	**5 c**	79
3	**2 d**	*n*Bu	H	Bn	Et (**4 a**)	1.0	**5 d**	70
4	**2 e**	Cyclopropyl	5-Br	Bn	Et (**4 a**)	0.5	**5 e**	83
5	**2 f**	Cyclopropyl	5-Cl	Bn	Et (**4 a**)	0.5	**5 f**	84
6	**2 g**	Cyclopropyl	5-F	Bn	Et (**4 a**)	0.4	**5 g**	82
7	**2 h**	Cyclopropyl	5-Me	Bn	Et (**4 a**)	0.5	**5 h**	84
8	**2 i**	Cyclopropyl	5-MeO	Bn	Et (**4 a**)	0.3	**5 i**	70
9	**2 j**	Cyclopropyl	6-Br	Bn	Et (**4 a**)	0.2	**5 j**	69
10	**2 k**	Cyclopropyl	6-Cl	Bn	Et (**4 a**)	0.4	**5 k**	78
11	**2 l**	Cyclopropyl	6-Me	Bn	Et (**4 a**)	2.0	**5 l**	85
12	**2 m**	Cyclopropyl	7-Br	Bn	Et (**4 a**)	0.2	**5 m**	63
13	**2 n**	Cyclopropyl	7-Cl	Bn	Et (**4 a**)	0.3	**5 n**	73
14	**2 o**	Cyclopropyl	7-F	Bn	Et (**4 a**)	0.2	**5 o**	72
15	**2 p**	Cyclopropyl	H	Allyl	Et (**4 a**)	2.0	**5 p**	74
16	**2 q**	Cyclopropyl	H	Anthracen-9-ylmethyl	Et (**4 a**)	4.0	**5 q**	80
17	**2 r**	Cyclopropyl	H	Me	Et (**4 a**)	2.0	**5 r**	80
18	**2 s**	Cyclopropyl	5-Br	CPh_3_	Et (**4 a**)	0.4	**5 s**	89
19	**2 e**	Cyclopropyl	5-Br	Bn	Bn (**4 b**)	0.3	**5 t**	82
20	**2 a**	Cyclopropyl	H	Bn	Bn (**4 b**)	7.0	**5 u**	86
21[c]	**2 a**	Cyclopropyl	H	Bn	*t*Bu (**4 c**)	10.0	**5 v**	49 (43)[d]

[a] *Reagents and conditions:* a) **2** (0.2 mmol), **4** (2.0 equiv), DABCO (20 mol %), tetrahydrofuran (2.0 mL), RT.

[b] Yield of isolated product.

[c] At reflux temperature.

[d] 43 % of **2 a** was recovered. PG=protecting group; Bn=benzyl.

**Figure 2:**
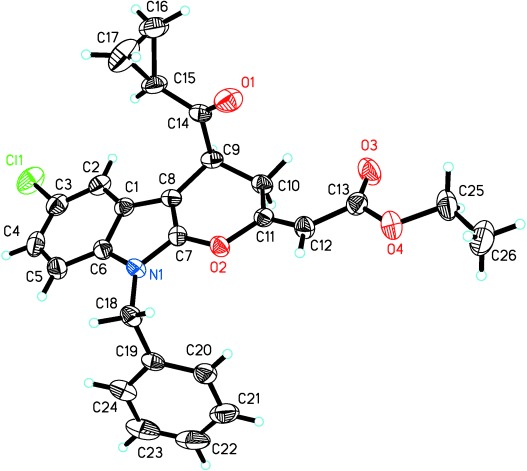
ORTEP drawing of 5 f.

On the other hand, a convenient one-pot synthesis of polycyclic indoles from propargyl benzyl ether **1** is also possible and is described in Scheme 3. As for substrates **1 a** (R^1^=cyclopropyl) and **1 b** (R^1^=cyclohexyl), polycyclic indoles **5 a** and **5 b** could be afforded in 52 % and 41 % yields, respectively. Whether electron-withdrawing (R^2^=5-Br) or electron-donating groups (R^2^=6-Me) present on the benzene ring, the reaction proceeded smoothly in both cases to give the desired cycloadducts **5 e** and **5 l** in 45–48 % yields.

**Scheme 3:**
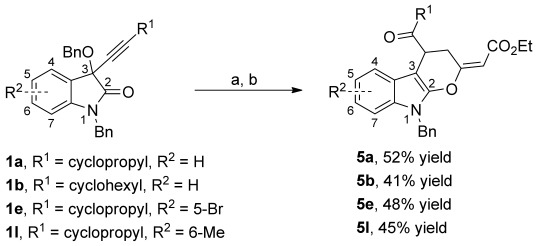
One-pot synthesis of polycyclic indoles. *Reagents and conditions:* a) [(Ph_3_P)AuCl]/AgOTf (5 mol %), H_2_O (1.0 equiv), CHCl_3_, RT, 3 h. b) 4a (2.0 mmol), DABCO (20 mol %), CHCl_3_, RT, 1 h.

To elucidate the rearrangement mechanism, an isotopic- labeling experiment has been performed (Scheme 4 A). Carrying out the reaction in the presence of H_2_^18^O (1.0 equiv) led to the formation of the corresponding product **2 a** in 32 % yield (60 % ^18^O) along with **3 a** in 27 % yield (40 % ^18^O; determined by ESI-MS analysis). Moreover, benzyl ether **3 a** could not be transformed to α,β-unsaturated ketone **2 a** under the standard conditions (Scheme 4 B).

**Scheme 4:**
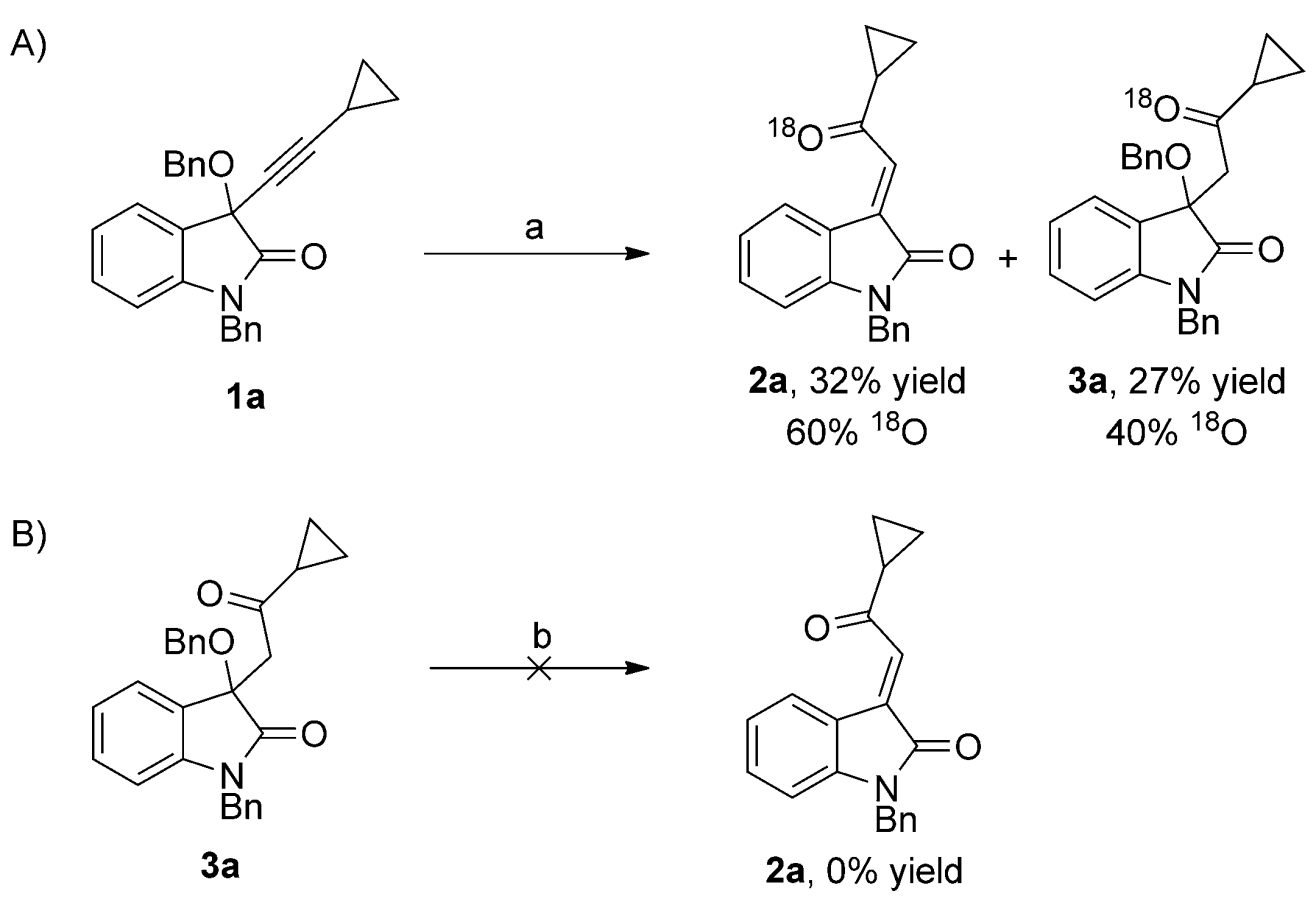
A) Isotopic-labeling experiment. *Reagents and conditions:* a) [(Ph_3_P)AuCl]/AgOTf (5 mol %), H_2_^18^O (1.0 equiv), CH_2_Cl_2_, RT, 2 h. B) Benzyl ether 3 a did not react to α,β-unsaturated ketone 2 a under the standard conditions. *Reagents and conditions:* b) [(Ph_3_P)AuCl]/AgOTf (5 mol %), CHCl_3_, RT, no reaction.

On the basis of above results, a plausible mechanisms for these reactions is outlined in Scheme 5. In cycle ***L***, coordination of gold(I) complex **A** to the alkyne forms intermediate **B**, which is attacked by water to form enol **D**. The tautomerization and hydrolysis of intermediate **D** produces benzyl ether **3 a**. Alternatively, nucleophilic attack of water on the alkyne moiety of intermediate **B** can also afford allenol **C** along with the release of HOBn, and which can further tautomerize to the corresponding conjugated enone **2 a** and regenerating the gold(I) complex **A**. In cycle ***R***, DABCO reacts with the allenic ester **4 a** to generate a zwitterionic intermediate **F**, which undergoes intermolecular Michael addition with enone **2 a** to produce intermediate **G**. Enolization of **G** forms oxo-anionic intermediate **H**, followed by an intramolecular nucleophilic attack to give 2,3-dihydropyran **I**. Subsequently, the facile single bond rotation affords the sterically favored intermediate **J**, and then the elimination takes place to give the polycyclic indole **5 a** along with the regeneration of the catalyst **E**.

**Scheme 5:**
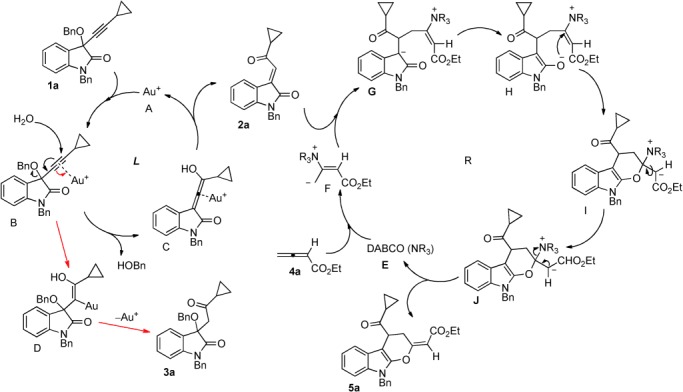
A plausible reaction mechanism for the rearrangement/[4+2] cycloaddition under sequential catalysis.

In conclusion, we have developed an efficient procedure for the sequential catalysis of rearrangement and [4+2] cycloaddition to construct the polycyclic indoles in good yields with high stereoselectivities from isatin derivatives and allenic esters. This transformation is rapid and practical. It can be performed under very mild conditions bearing various substituents at many positions. Further applications of this chemistry and more detailed mechanistic investigation are under way in our laboratory.

## Experimental Section

**General procedure for gold(I)-catalyzed rearrangement of pro** **pargyl benzyl ethers under the standard reaction conditions:** Under ambient atmosphere, propargyl benzyl ethers **1** (0.2 mmol) and H_2_O (1.0 equiv) were dissolved in CHCl_3_ (2.0 mL) in a Schlenk tube, and [(Ph_3_P)AuCl]/AgOTf (5 mol %) were added. The reaction mixture was stirred at RT until the reaction completed (determined using thin-layer chromatography). The solvent was removed in vacuo, and the residue was purified using flash column chromatography (SiO_2_) to give corresponding products **2** in moderate yields.

**General procedure for DABCO-catalyzed [4+2] cycloaddition of isatin-derived α,β-unsaturated ketones with α-allenic ester under standard reaction conditions:** Under argon atmosphere, α,β-unsaturated ketones **2** (0.2 mmol) and 1,4-diazabicyclo[2.2.2]octane (DABCO; 20 mol %) were dissolved in tetrahydrofuran (THF; 2.0 mL) in a Schlenk tube, α-allenic ester **4** was added. The reaction mixture was stirred at RT until the reaction completed (determined using thin-layer chromatography). The solvent was removed in vacuo, and the residue was purified by flash column chromatography (SiO_2_) to give corresponding products **5** in good yields.

Experimental procedures and spectral data for all new compounds are available in the Supporting Information.

## Supplementary material

Detailed facts of importance to specialist readers are published as ”Supporting Information”. Such documents are peer-reviewed, but not copy-edited or typeset. They are made available as submitted by the authors.
