# Supplementary material for: Highly Stereoselective Synthesis of Polycyclic Indoles through Rearrangement/[4+2] Cycloaddition under Sequential Catalysis
Source: ChemistryOpen. 2012 Sep 11;1(5):215–20. doi: 10.1002/open.201200028 (PMC3922592; doi:10.1002/open.201200028)
Supplement: Supplementary file 1 [file open0001-0215-SD1.pdf]

ChemistryOPEN

Supporting Information

© 2012 The Authors. Published by Wiley-VCH Verlag GmbH & Co. KGaA, Weinheim

Highly Stereoselective Synthesis of Polycyclic Indoles through Rearrangement/[4+2] Cycloaddition under Sequential Catalysis

Di-Han Zhang and Min Shi*[a]

open_201200028_sm_miscellaneous_information.pdf

Supporting Information

Contents

General remarks S2

General procedure for the reactions S2-S3

Spectroscopic data of compounds **1**, **2**, **3** and **5** S4-S117

GC for BnOH and reaction mixture of **1a** under the standard conditions S118-S120

General Remarks. ^{1}H and ^{13}C NMR spectra were recorded at 400 (or 300) and 100 (or 75) MHz, respectively. Mass and HRMS spectra were recorded by ESI or EI method. The employed solvents were dry up by standard methods when necessary. Commercially obtained reagents were used without further purification. All reactions were monitored by TLC with silica gel coated plates. Flash column chromatography was carried out using 300-400 mesh silica gel at increased pressure.

General procedure for gold(I)-catalyzed rearrangement of propargyl benzyl ethers under the standard reaction conditions. Under ambient atmosphere, propargyl benzyl ethers **1** (0.2 mmol) and H_2O (1.0 equiv) were dissolved in $CHCl_3$ (2.0 mL) in an Schlenk tube, [(PPh_3)AuCl]/AgOTf (5 mol %) were added. Then, the reaction mixture was stirred at room temperature until the reaction completed. The solvent was removed under reduced pressure and the residue was purified by a flash column chromatography (SiO_2) to give the corresponding products **2** in moderate yields.

General procedure for DABCO-catalyzed [4+2] cycloaddition of isatin derived α,β–unsaturated ketones with α-allenic ester under the standard reaction conditions. Under argon atmosphere, α,β–unsaturated ketones **2** (0.2 mmol) and DABCO (20 mol %) was dissolved in THF (2.0 mL) in an Schlenk tube, α-allenic ester **4** was added. Then, the reaction mixture was stirred at room temperature until the reaction completed. The solvent was removed under reduced pressure and the residue was purified by a flash column chromatography (SiO_2) to give the corresponding products **5** in good yields.

General procedure for one-pot rearrangement/[4+2] cycloaddition to polycyclic indoles under the standard reaction conditions. Under argon atmosphere, propargyl benzyl ethers **1** (0.2 mmol) and H_2O (1.0 equiv) were dissolved in $CHCl_3$ (2.0 mL) in an Schlenk tube, [(PPh_3)AuCl]/AgOTf (5 mol %) were added, the reaction mixture was stirred at room temperature for 3 hours. Then, DABCO (20 mol %) and α-allenic ester **4a** were added . The reaction mixture was stirred at room temperature until the reaction completed. The solvent was removed under reduced pressure and the residue was purified by a flash column

chromatography (SiO_2) to give the corresponding products **5** in moderate yields.

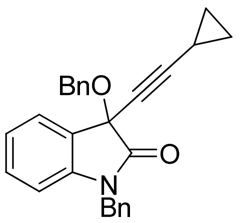

Compound 1a: A white solid. Mp: 127-129 °C. IR (neat) ν 3029, 2926, 1713, 1611, 1492, 1348, 1025, 759, 699 cm^{-1}. ^{1}H NMR ($CDCl_3$, 400 MHz, TMS) δ 0.71-0.80 (m, 4H), 1.26-1.34 (m, 1H), 4.82-4.92 (m, 4H), 6.65 (d, J = 7.6 Hz, 1H), 7.04 (dt, J = 0.8 Hz, J = 7.6 Hz, 1H), 7.18 (dt, J = 1.2 Hz, J = 8.0 Hz, 1H), 7.21-7.32 (m, 8H), 7.36 (d, J = 8.0 Hz, 2H), 7.48 (dd, J = 0.8 Hz, J = 7.6 Hz, 1H). ^{13}C NMR ($CDCl_3$, 100 MHz, TMS) δ -0.3, 8.5, 43.7, 67.4, 69.9, 74.1, 92.9, 109.5, 123.3, 124.7, 127.1, 127.5, 127.6, 128.1, 128.2, 128.4, 128.7, 130.0, 135.2, 137.8, 142.1, 172.2. MS (ESI) *m/z* 411.2 $(M+NH_4)^+$. HRMS (ESI) Calcd. for $C_{27}H_{23}NO_2Na$ $(M+Na)^+$: 416.1621, Found: 416.1632.

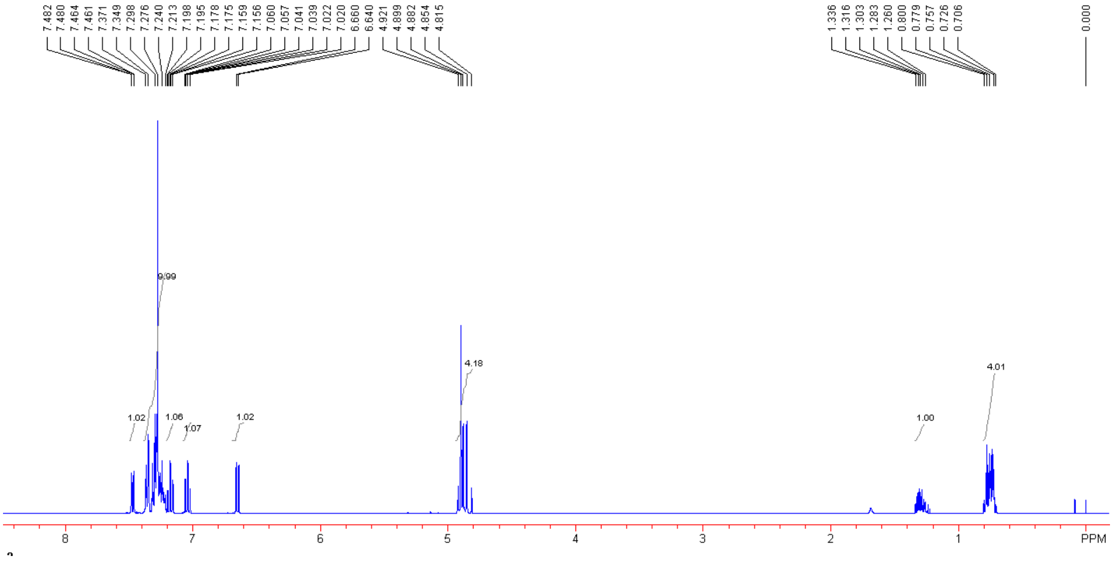

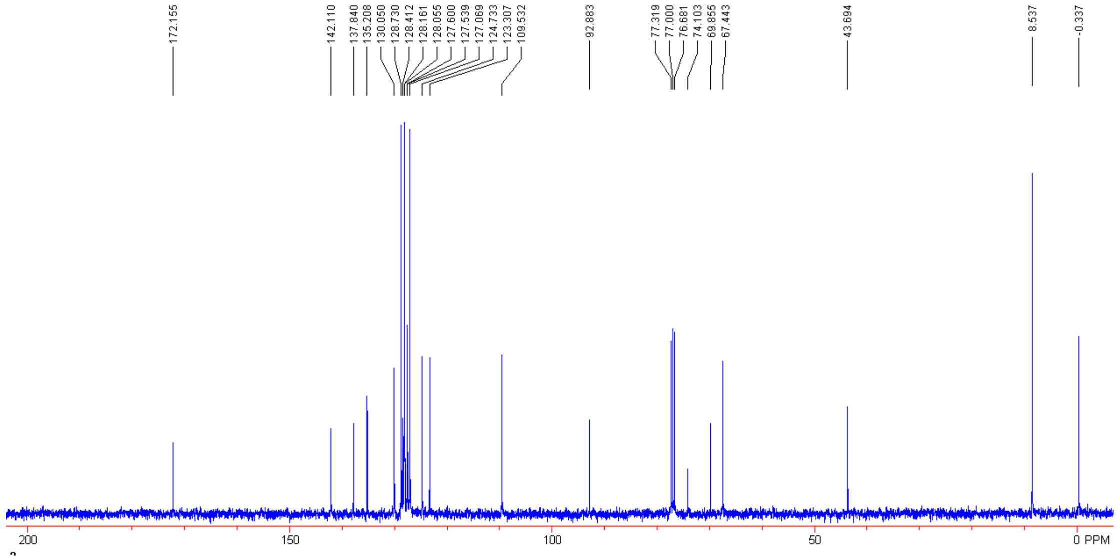
172.155
142.110
137.840
135.208
130.050
128.730
128.412
128.161
128.055
127.600
127.539
127.069
124.733
123.307
109.532
92.883
77.319
77.000
76.681
74.103
69.855
67.443
43.694
8.537
-0.337
200
150
100
50
0 PPM

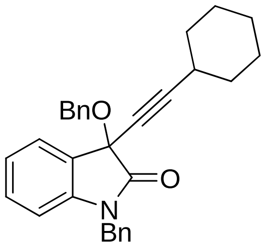

Compound 1b: A light yellow oil. IR (neat) ν 2928, 2853, 1724, 1612, 1347, 1177, 732, 696 cm^{-1}. ^{1}H NMR ($CDCl_3$, 400 MHz, TMS) δ 1.24-1.31 (m, 3H), 1.43-1.49 (m, 3H), 1.63-1.66 (m, 2H), 1.73-1.75 (m, 2H), 2.43-2.48 (m, 1H), 4.81 (s, 2H), 5.00 (s, 2H), 6.61 (d, *J* = 8.0 Hz, 1H), 7.00 (t, *J* = 7.6 Hz, 1H), 7.12 (t, *J* = 7.6 Hz, 1H), 7.18-7.29 (m, 8H), 7.37 (d, *J* = 7.6 Hz, 2H), 7.48 (d, *J* = 7.2 Hz, 1H). ^{13}C NMR ($CDCl_3$, 100 MHz, TMS) δ 24.4, 25.6, 28.8, 31.9, 43.4, 67.4, 73.9, 74.7, 93.7, 109.3, 123.1, 124.5, 126.9, 127.4, 127.9, 128.0, 128.5, 129.9, 135.1, 137.8, 141.9, 172.1. MS (ESI) *m/z* 453.1 $(M+NH_4)^+$. HRMS (ESI) Calcd. for $C_{30}H_{29}NO_2Na$ $(M+Na)^+$: 458.2090, Found: 458.2100.

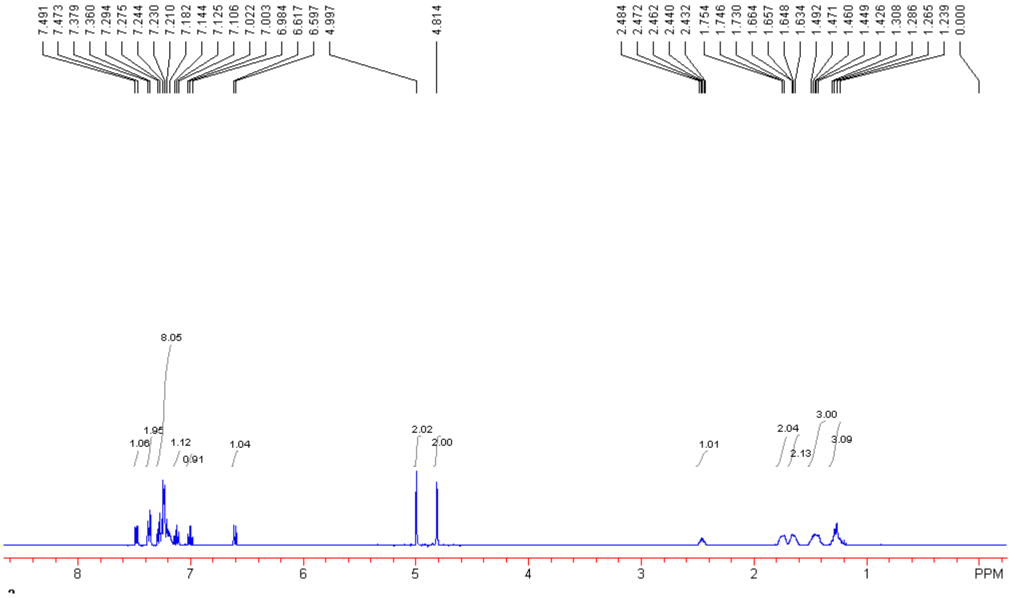

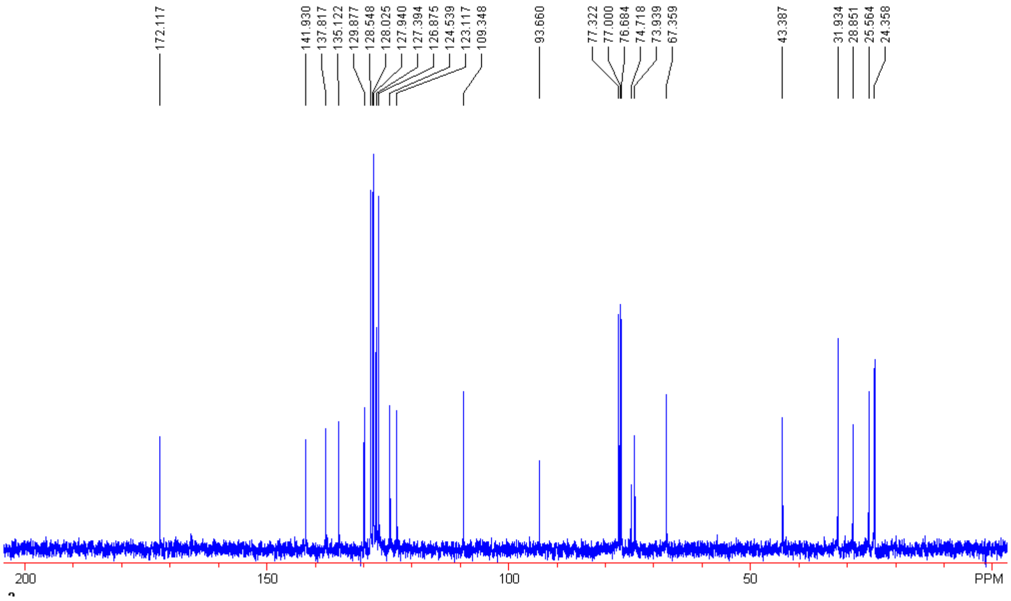

172.117
141.930
137.817
135.122
129.877
128.548
128.025
127.940
127.394
126.875
124.539
123.117
109.348
93.660
77.322
77.000
76.684
74.718
73.939
67.359
43.387
31.934
28.851
25.564
24.358
200
150
100
50
PPM

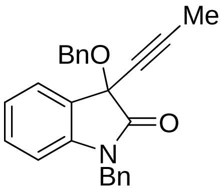

Compound 1c: A light yellow solid. Mp: 158-160 ^{o}C. IR (neat) ν 2920, 1720, 1611, 1346, 1104, 752 cm^{-1}. ^{1}H NMR ($CDCl_3$, 300 MHz, TMS) δ 1.89 (s, 3H), 4.86-4.89 (m, 4H), 6.66 (d, *J* = 8.1 Hz, 1H), 7.05 (t, *J* = 7.5 Hz, 1H), 7.16-7.37 (m, 11H), 7.50 (d, *J* = 7.5 Hz, 1H). ^{13}C NMR ($CDCl_3$, 75 MHz, TMS) δ 4.0, 43.7, 67.4, 73.9, 74.1, 85.4, 109.6, 123.3, 124.7, 127.0, 127.56, 127.58, 128.06, 128.14, 128.7, 130.1, 135.1, 137.6, 142.1, 172.2. MS (ESI) *m/z* 390.0 (M+Na)$^{+}$. HRMS (ESI) Calcd. for $C_{25}H_{21}NO_2Na$ (M+Na)$^{+}$: 390.1489, Found: 390.1476.

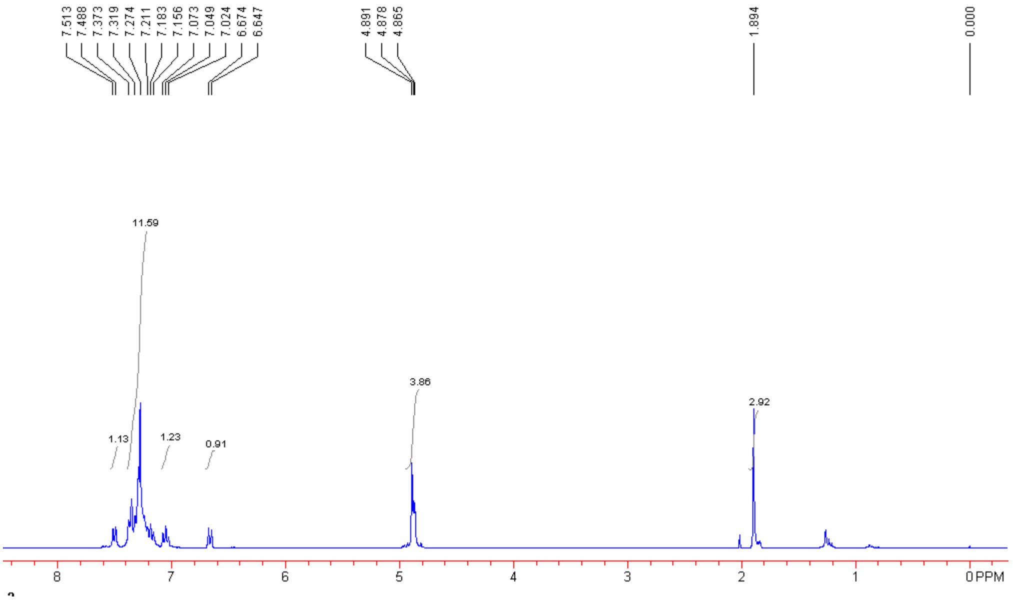

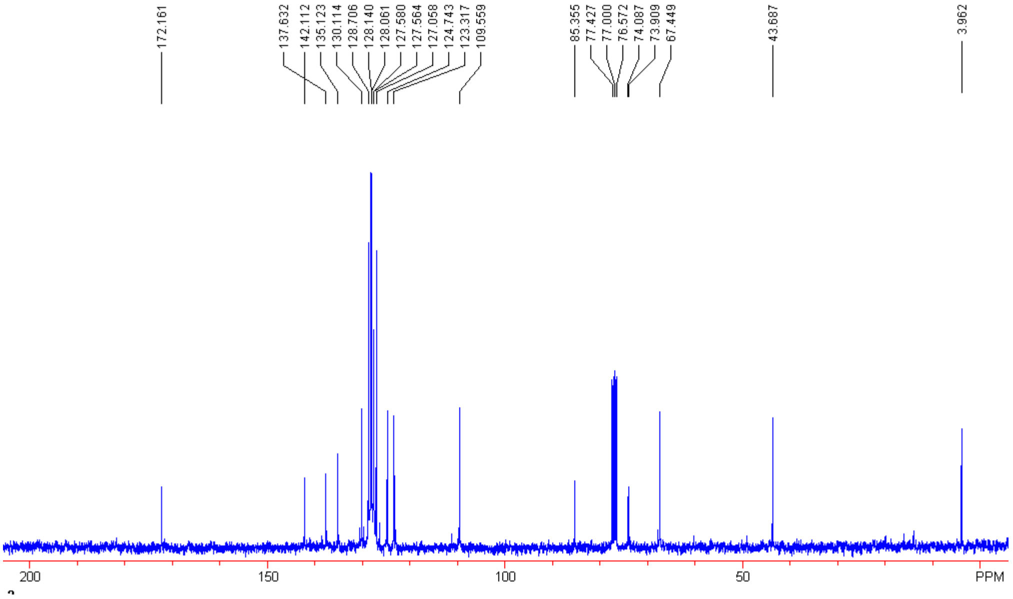

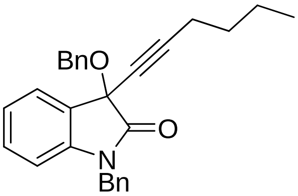

Compound 1d: A light yellow solid. Mp: 77-79 °C. IR (neat) ν 2958, 2927, 1714, 1610, 1349, 1181, 754 cm^{-1}. ^{1}H NMR ($CDCl_3$, 400 MHz, TMS) δ 0.87 (t, *J* = 7.6 Hz, 3H), 1.34-1.43 (m, 2H), 1.46-1.53 (m, 2H), 2.26 (t, *J* = 7.2 Hz, 2H), 4.85 (s, 2H), 4.95 (s, 2H), 6.64 (d, *J* = 8.0 Hz, 1H), 7.03 (t, *J* = 7.6 Hz, 1H), 7.14-7.31 (m, 9H), 7.37 (d, *J* = 8.0 Hz, 2H), 7.49 (d, *J* = 7.2 Hz, 1H). ^{13}C NMR ($CDCl_3$, 100 MHz, TMS) δ 13.4, 18.5, 21.8, 30.2, 43.6, 67.4, 74.0, 74.7, 89.8, 109.5, 123.2, 124.7, 127.0, 127.49, 127.52, 128.0, 128.1, 128.4, 128.6, 130.0, 135.2, 137.8, 142.0, 172.2. MS (ESI) *m/z* 427.1 $(M+NH_4)^+$. HRMS (ESI) Calcd. for $C_{28}H_{27}NO_2Na$ $(M+Na)^+$: 432.1934, Found: 432.1944.

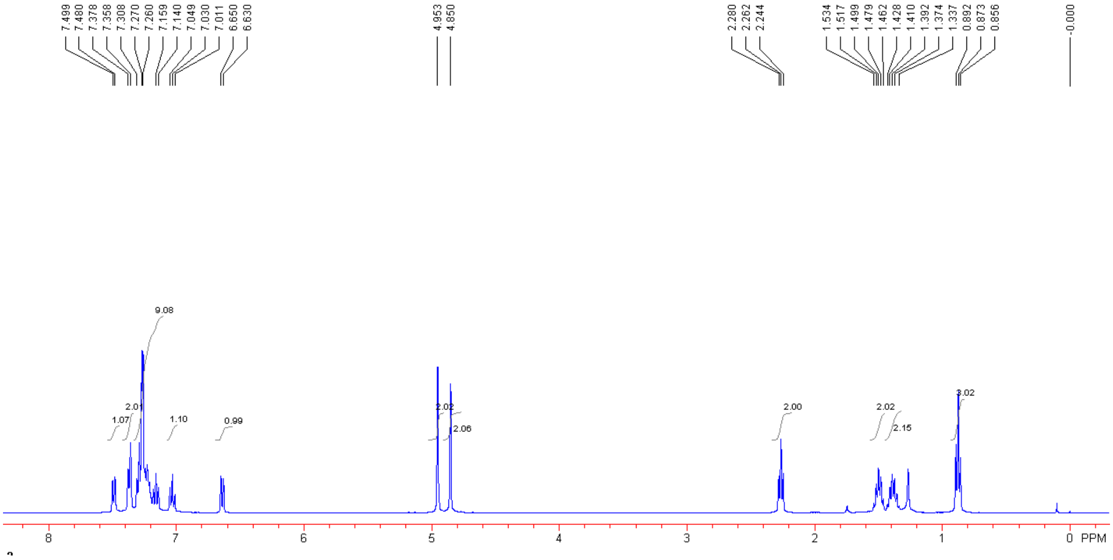

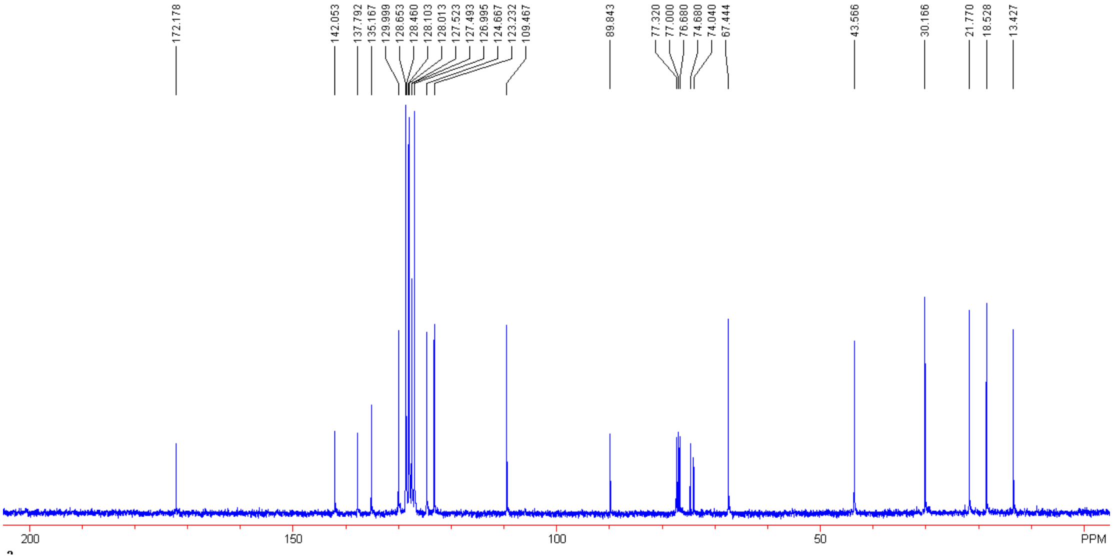

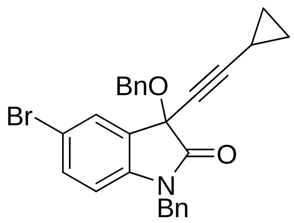

Compound 1e: A light yellow oil. IR (neat) ν 2920, 2851, 1723, 1606, 1480, 1173, 733 cm^{-1}. ^{1}H NMR ($CDCl_3$, 400 MHz, TMS) δ 0.71-0.74 (m, 4H), 1.24-1.30 (m, 1H), 4.73 (d, J = 15.6 Hz, 1H), 4.81 (d, J = 15.6 Hz, 1H), 4.95 (d, J = 11.2 Hz, 1H), 4.98 (d, J = 11.2 Hz, 1H), 6.47 (d, J = 8.4 Hz, 1H), 7.17-7.29 (m, 9H), 7.34 (d, J = 7.6 Hz, 2H), 7.57 (s, 1H). ^{13}C NMR ($CDCl_3$, 100 MHz, TMS) δ -0.6, 8.4, 43.4, 67.4, 69.0, 73.5, 93.5, 110.9, 115.6, 126.8, 127.48, 127.51, 127.6, 127.8, 128.0, 128.6, 130.1, 132.6, 134.5, 137.3, 140.8, 171.3. MS (ESI) m/z 489.0 $(M+NH_4)^+$. HRMS (ESI) Calcd. for $C_{27}H_{22}NO_2BrNa$ $(M+Na)^+$: 494.0726, Found: 494.0732.

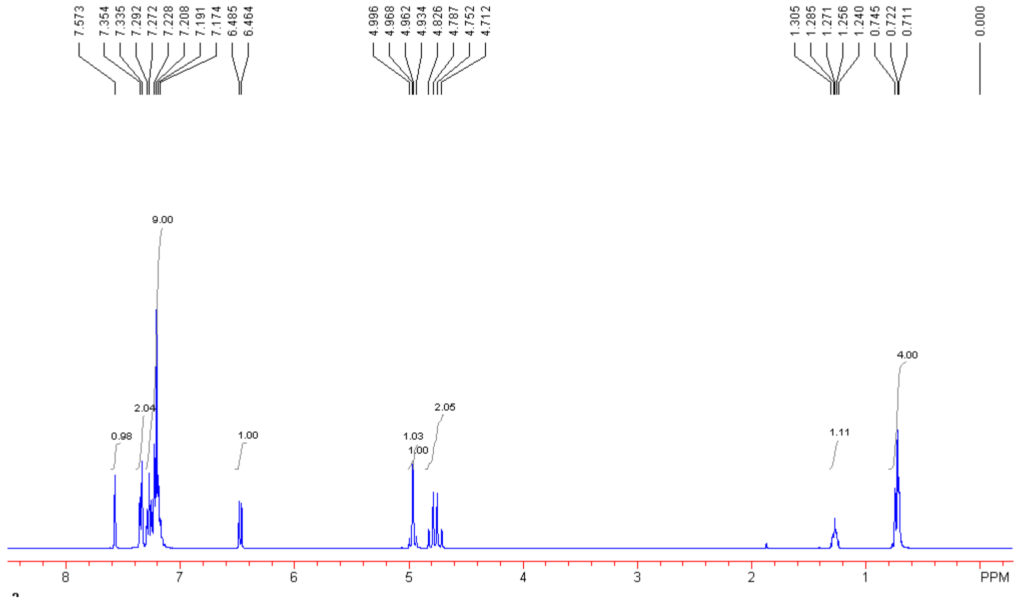

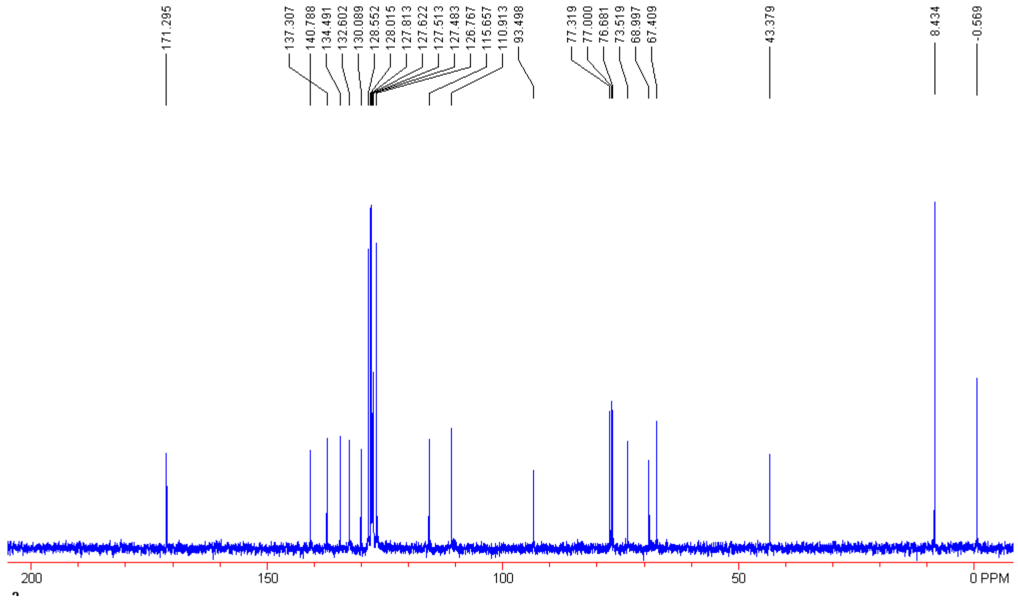
171.295
137.307
140.788
134.491
132.602
130.089
128.552
128.015
127.813
127.622
127.513
127.483
126.767
115.657
110.913
93.498
77.319
77.000
76.681
73.519
68.997
67.409
43.379
8.434
-0.569
200
150
100
50
0 PPM

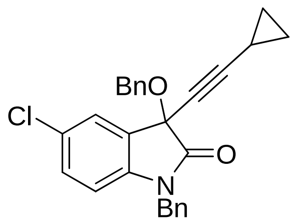

Compound 1f: A light yellow solid. Mp: 135-137 °C. IR (neat) ν 3032, 2937, 1717, 1482, 1173, 1019, 738 cm^{-1}. ^{1}H NMR ($CDCl_3$, 400 MHz, TMS) δ 0.70-0.79 (m, 4H), 1.26-1.32 (m, 1H), 4.76 (d, *J* = 15.6 Hz, 1H), 4.83 (d, *J* = 15.6 Hz, 1H), 4.94 (d, *J* = 11.2 Hz, 1H), 4.97 (d, *J* = 11.2 Hz, 1H), 6.53 (d, *J* = 8.4 Hz, 1H), 7.09 (dd, *J* = 2.0 Hz, *J* = 8.0 Hz, 1H), 7.21-7.30 (m, 8H), 7.35 (d, *J* = 6.8 Hz, 2H), 7.44 (d, *J* = 2.0 Hz, 1H). ^{13}C NMR ($CDCl_3$, 100 MHz, TMS) δ -0.5, 8.5, 43.5, 67.5, 69.0, 73.7, 93.6, 110.5, 125.0, 126.9, 127.56, 127.61, 127.9, 128.1, 128.5, 128.6, 129.8, 129.9, 134.6, 137.4, 140.4, 171.5. MS (ESI) *m/z* 450.0 $(M+Na)^+$. HRMS (ESI) Calcd. for $C_{27}H_{22}NO_2ClNa$ $(M+Na)^+$: 450.1231, Found: 450.1229.

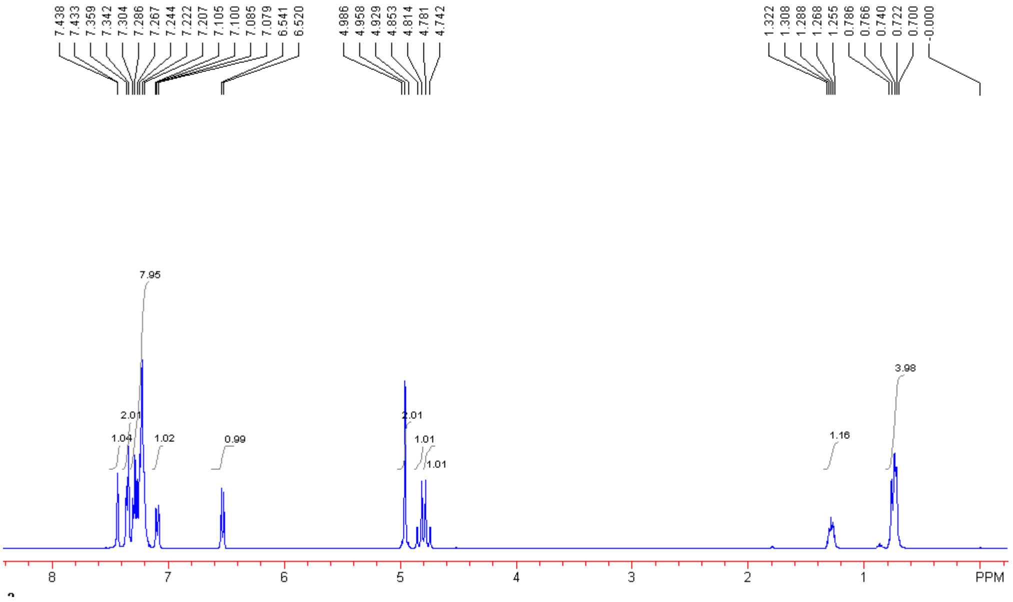

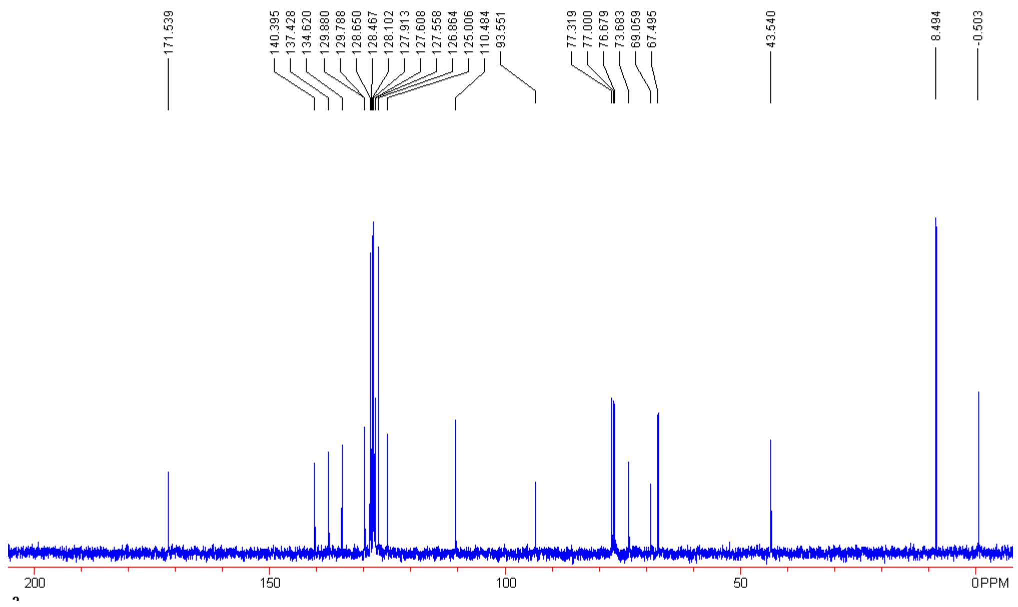
171.539
140.395
137.428
134.620
129.880
129.788
128.650
128.467
128.102
127.913
127.608
127.558
126.864
125.006
110.484
93.551
77.319
77.000
76.679
73.683
69.059
67.495
43.540
8.494
-0.503
200
150
100
50
0PPM

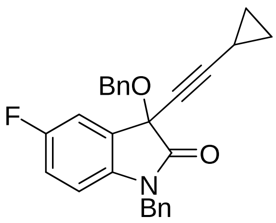

Compound 1g: A light yellow solid. Mp: 147-149 °C. IR (neat) ν 3033, 2937, 1710, 1492, 1172, 1019, 700 cm^{-1}. ^{1}H NMR ($CDCl_3$, 400 MHz, TMS) δ 0.69-0.78 (m, 4H), 1.25-1.32 (m, 1H), 4.78 (d, *J* = 15.6 Hz, 1H), 4.85 (d, *J* = 15.6 Hz, 1H), 4.95 (s, 2H), 6.52-6.55 (m, 1H), 6.84 (dd, *J* = 2.4 Hz, *J* = 8.8 Hz, 1H), 7.20-7.30 (m, 9H), 7.35 (d, *J* = 7.2 Hz, 2H). ^{13}C NMR ($CDCl_3$, 100 MHz, TMS) δ -0.5, 8.4, 43.6, 67.5, 69.2, 73.9, 93.4, 110.2 (d, *J* = 7.9 Hz), 112.6 (d, *J* = 25.0 Hz), 116.2 (d, *J* = 23.2 Hz), 126.9, 127.6 (d, *J* = 2.1 Hz), 127.9, 128.1, 128.6, 129.8 (d, *J* = 7.8 Hz), 134.8, 137.5, 137.8, 159.2 (d, *J* = 241.0 Hz), 171.8. ^{19}F NMR ($CDCl_3$, 376 MHz, CF_3COOH) δ -119.0 ~ -118.9 (m). MS (ESI) *m/z* 434.0 $(M+Na)^+$. HRMS (ESI) Calcd. for $C_{27}H_{22}NO_2FNa$ $(M+Na)^+$: 434.1527, Found: 434.1531.

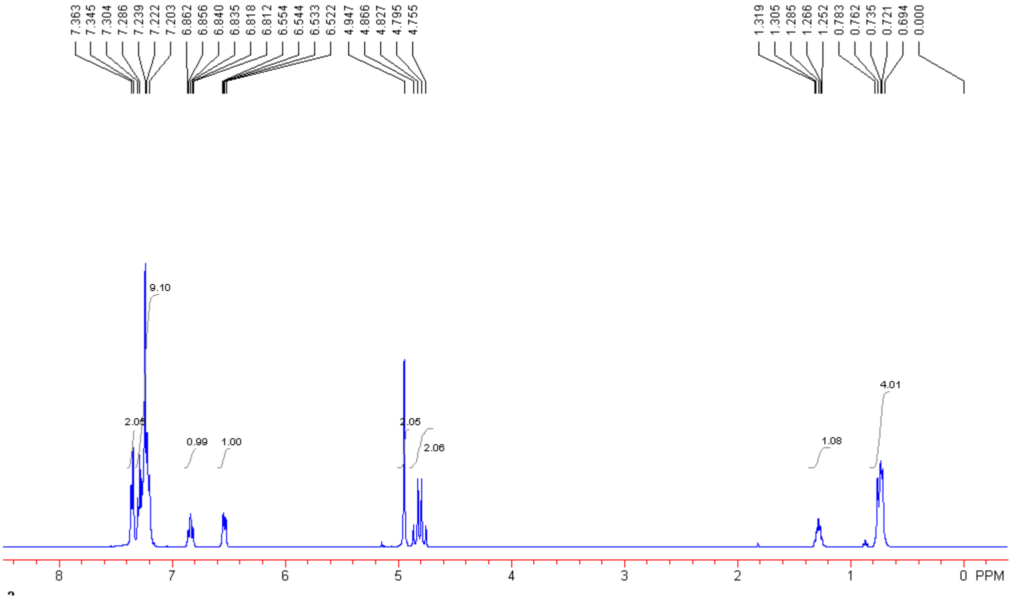

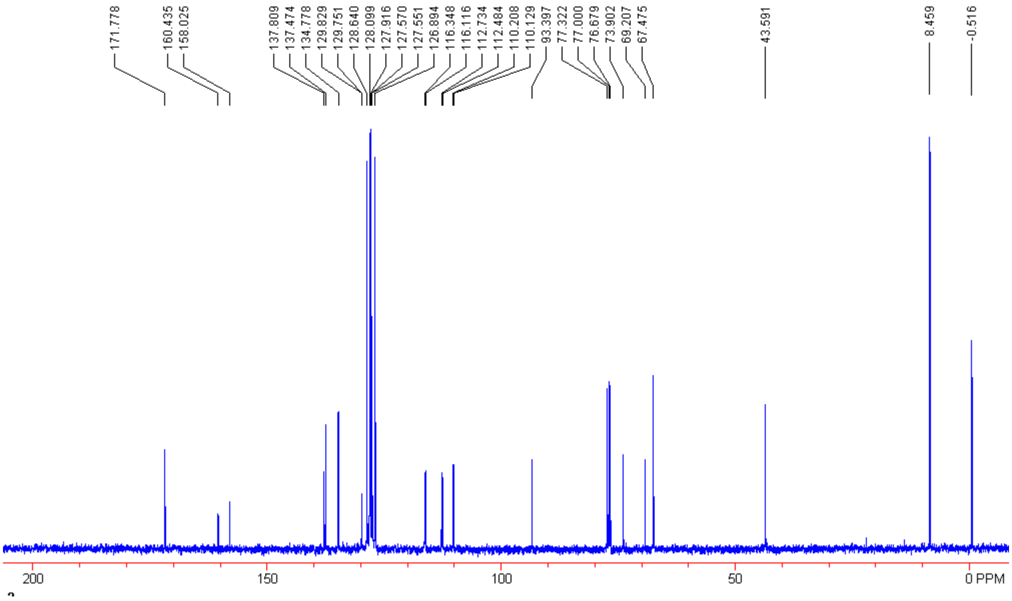
171.778
160.435
158.025
137.809
137.474
134.778
129.829
129.751
128.640
128.099
127.916
127.570
127.551
126.894
116.348
116.116
112.734
112.484
110.208
110.129
93.397
77.322
77.000
76.679
73.902
69.207
67.475
43.591
8.459
-0.516
200
150
100
50
0 PPM

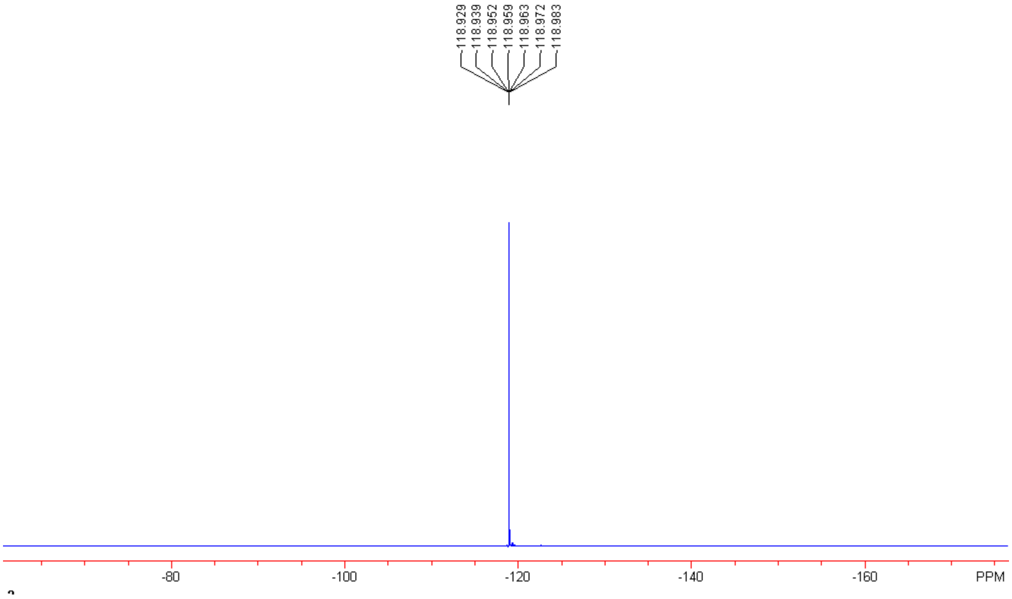
-118.929
-118.939
-118.952
-118.959
-118.963
-118.972
-118.983
-80
-100
-120
-140
-160
PPM

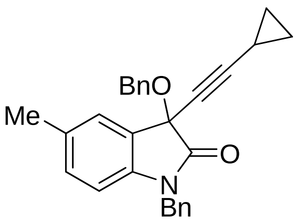

Compound 1h: A light yellow solid. Mp: 114-116 °C. IR (neat) ν 3032, 2929, 1731, 1604, 1336, 1017, 732 cm^{-1}. ^{1}H NMR ($CDCl_3$, 400 MHz, TMS) δ 0.71-0.75 (m, 4H), 1.24-1.30 (m, 1H), 2.23 (s, 3H), 4.75 (d, J = 15.6 Hz, 1H), 4.83 (d, J = 15.6 Hz, 1H), 4.93 (s, 2H), 6.50 (d, J = 8.0 Hz, 1H), 6.92 (d, J = 7.6 Hz, 1H), 7.17-7.28 (m, 9H), 7.36 (d, J = 6.8 Hz, 2H). ^{13}C NMR ($CDCl_3$, 100 MHz, TMS) δ -0.5, 8.4, 20.7, 43.4, 67.2, 69.9, 74.0, 92.6, 109.2, 125.2, 126.8, 127.3, 127.8, 128.0, 128.1, 128.5, 130.2, 132.7, 135.1, 137.7, 139.4, 171.9. MS (ESI) *m/z* 430.1 $(M+Na)^+$. HRMS (ESI) Calcd. for $C_{28}H_{25}NO_2Na$ $(M+Na)^+$: 430.1778, Found: 430.1766.

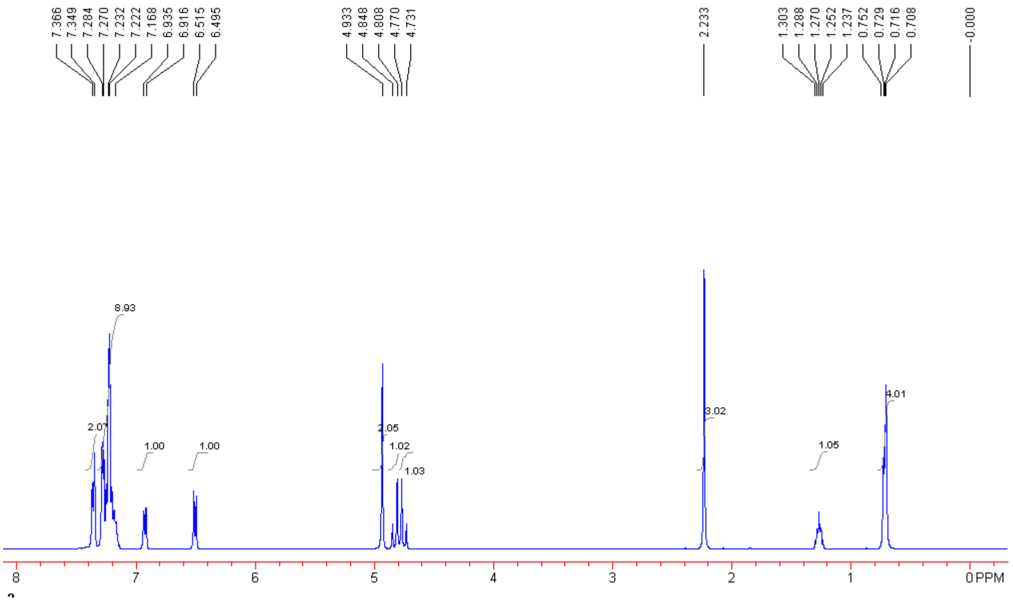

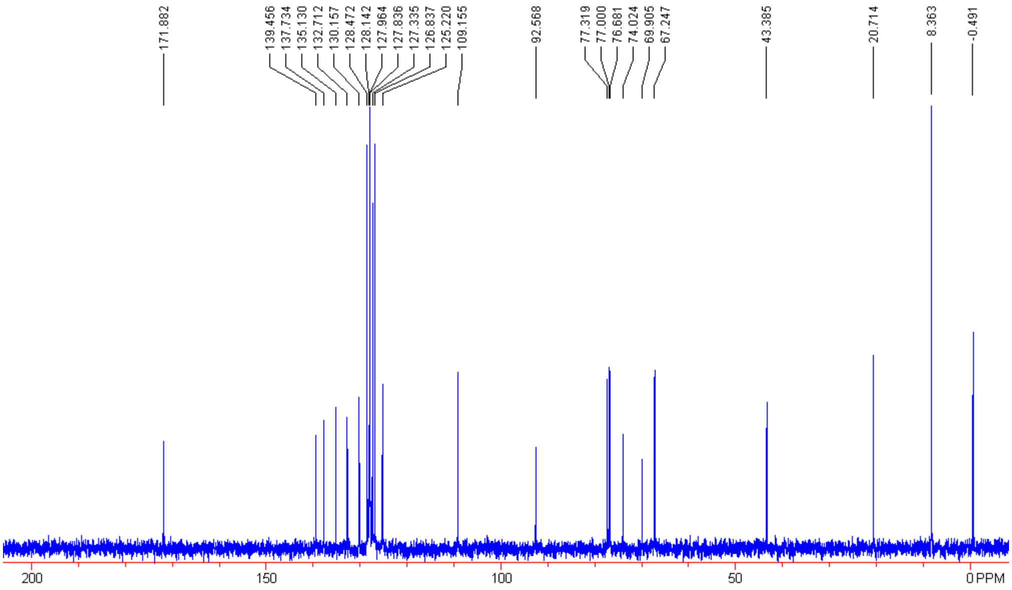
171.882
139.456
137.734
135.130
132.712
130.157
128.472
128.142
127.964
127.836
127.335
126.837
125.220
109.155
92.568
77.319
77.000
76.681
74.024
69.905
67.247
43.385
20.714
8.363
-0.491
200
150
100
50
0 PPM

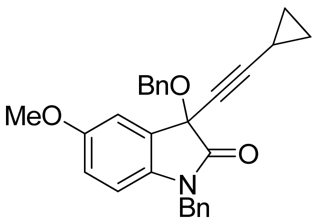

Compound 1i: A light yellow solid. Mp: 116-118 °C. IR (neat) ν 2928, 2833, 1700, 1493, 1180, 1015, 740 cm^{-1}. ^{1}H NMR ($CDCl_3$, 400 MHz, TMS) δ 0.73-0.78 (m, 4H), 1.28-1.32 (m, 1H), 3.71 (s, 3H), 4.78-4.92 (m, 4H), 6.54 (d, *J* = 8.4 Hz, 1H), 6.70 (dd, *J* = 2.4 Hz, *J* = 8.4 Hz, 1H), 7.08 (d, *J* = 2.4 Hz, 1H), 7.21-7.31 (m, 8H), 7.36 (d, *J* = 7.2 Hz, 2H). ^{13}C NMR ($CDCl_3$, 100 MHz, TMS) δ -0.4, 8.5, 43.7, 55.6, 67.4, 69.9, 74.4, 92.8, 110.0, 111.6, 114.6, 127.0, 127.5, 128.0, 128.1, 128.6, 129.4, 135.2, 135.3, 137.7, 156.3, 171.9. MS (ESI) *m/z* 446.0 $(M+Na)^+$. HRMS (ESI) Calcd. for $C_{28}H_{25}NO_3Na$ $(M+Na)^+$: 446.1727, Found: 446.1724.

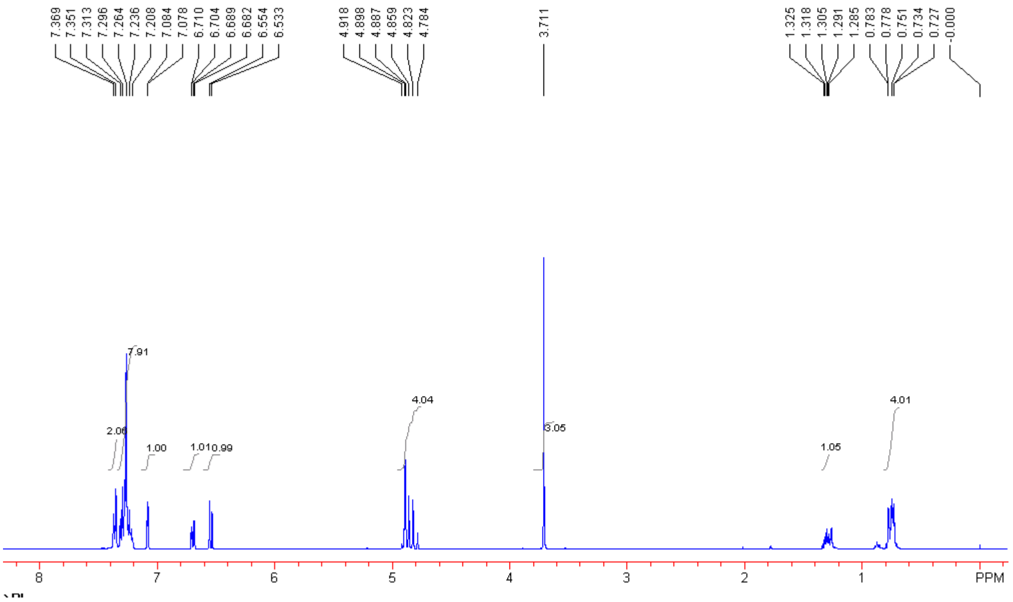

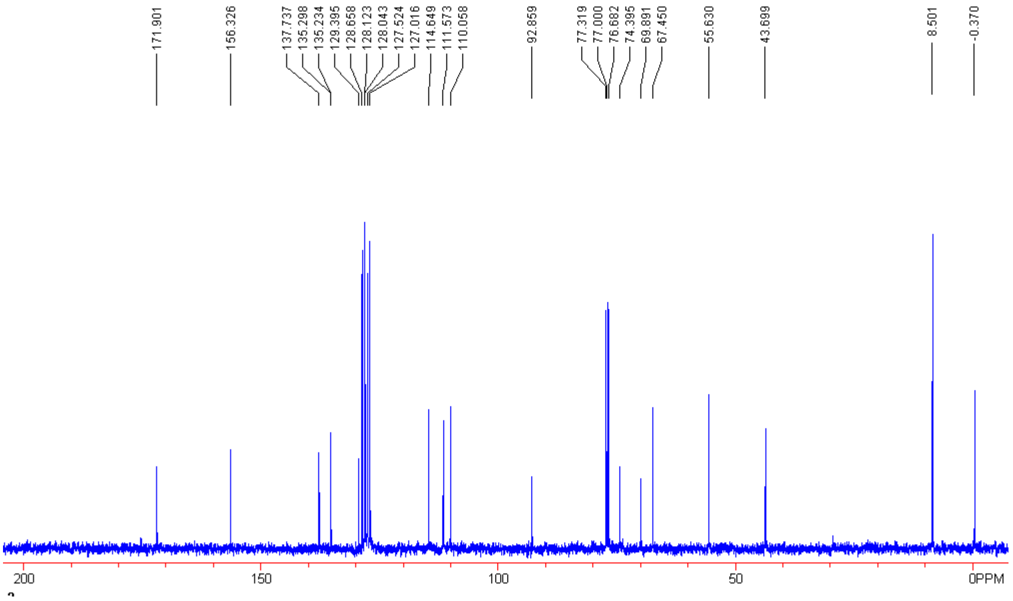
171.901
156.326
137.737
135.298
135.234
129.395
128.658
128.123
128.043
127.524
127.016
114.649
111.573
110.058
92.859
77.319
77.000
76.682
74.395
69.891
67.450
55.630
43.699
8.501
-0.370
200
150
100
50
0PPM

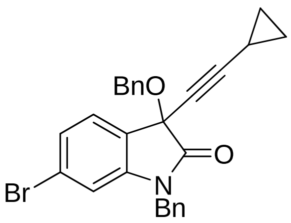

Compound 1j: A light yellow solid. Mp: 59-61 °C. IR (neat) ν 3031, 2923, 1726, 1603, 1485, 1058, 734 cm^{-1}. ^{1}H NMR ($CDCl_3$, 400 MHz, TMS) δ 0.70-0.72 (m, 4H), 1.22-1.29 (m, 1H), 4.72 (d, *J* = 15.6 Hz, 1H), 4.79 (d, *J* = 15.6 Hz, 1H), 4.92 (d, *J* = 11.2 Hz, 1H), 4.95 (d, *J* = 11.2 Hz, 1H), 6.80 (s, 1H), 7.12-7.35 (m, 12H). ^{13}C NMR ($CDCl_3$, 100 MHz, TMS) δ -0.6, 8.4, 43.4, 67.3, 69.1, 73.3, 93.1, 112.5, 123.5, 125.8, 125.9, 126.7, 127.1, 127.4, 127.5, 127.7, 128.0, 128.6, 134.4, 137.3, 143.1, 171.6. MS (ESI) *m/z* 494.0 $(M+Na)^+$. HRMS (ESI) Calcd. for $C_{27}H_{22}NO_2BrNa$ $(M+Na)^+$: 494.0726, Found: 494.0727.

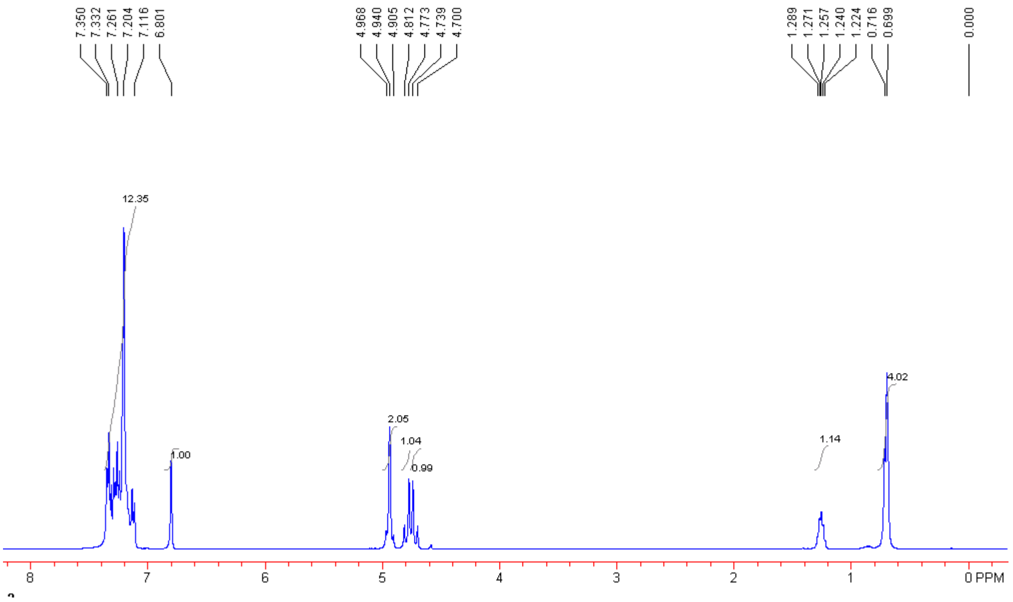

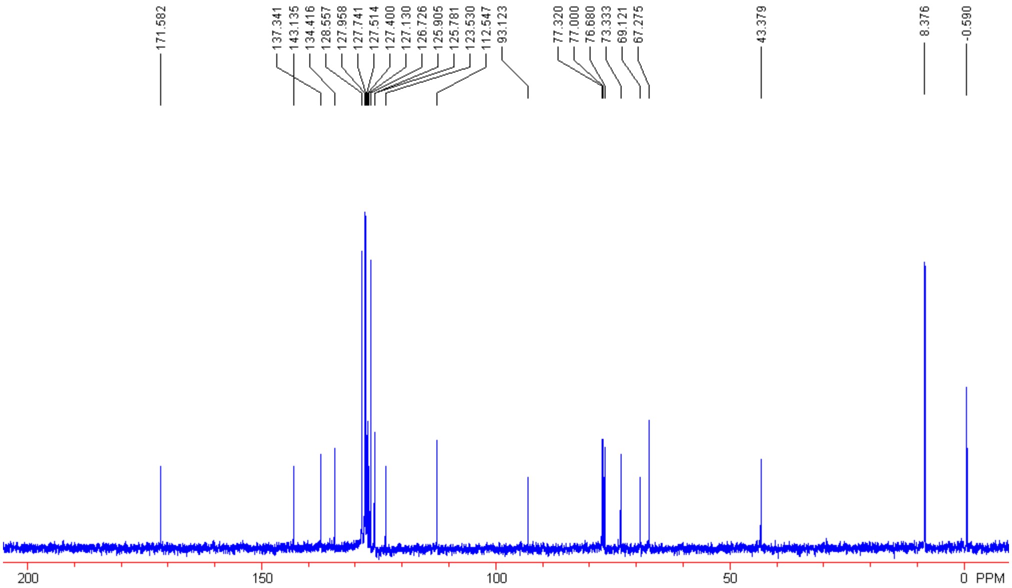
171.582
137.341
143.135
134.416
128.557
127.958
127.741
127.514
127.400
127.130
126.726
125.905
125.781
123.530
112.547
93.123
77.320
77.000
76.680
73.333
69.121
67.275
43.379
8.376
-0.590
200
150
100
50
0 PPM

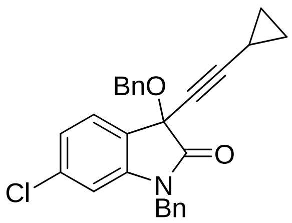

Compound 1k: A light yellow oil. IR (neat) ν 3031, 2927, 1727, 1608, 1488, 1070, 695 cm^{-1}. ^{1}H NMR ($CDCl_3$, 400 MHz, TMS) δ 0.71-0.82 (m, 4H), 1.28-1.34 (m, 1H), 4.81 (d, *J* = 16.0 Hz, 1H), 4.87 (d, *J* = 16.0 Hz, 1H), 4.91 (s, 2H), 6.66 (d, *J* = 1.6 Hz, 1H), 7.02 (dd, *J* = 2.0 Hz, *J* = 8.0 Hz, 1H), 7.24-7.38 (m, 11H). ^{13}C NMR ($CDCl_3$, 100 MHz, TMS) δ -0.4, 8.6, 43.8, 67.6, 69.3, 73.6, 93.4, 110.1, 123.2, 125.7, 126.9, 127.0, 127.7, 127.8, 128.1, 128.2, 128.9, 134.7, 135.8, 137.6, 143.3, 172.1. MS (ESI) *m/z* 445.1 $(M+NH_4)^+$. HRMS (ESI) Calcd. for $C_{27}H_{22}NO_2ClNa$ $(M+Na)^+$: 450.1231, Found: 450.1231.

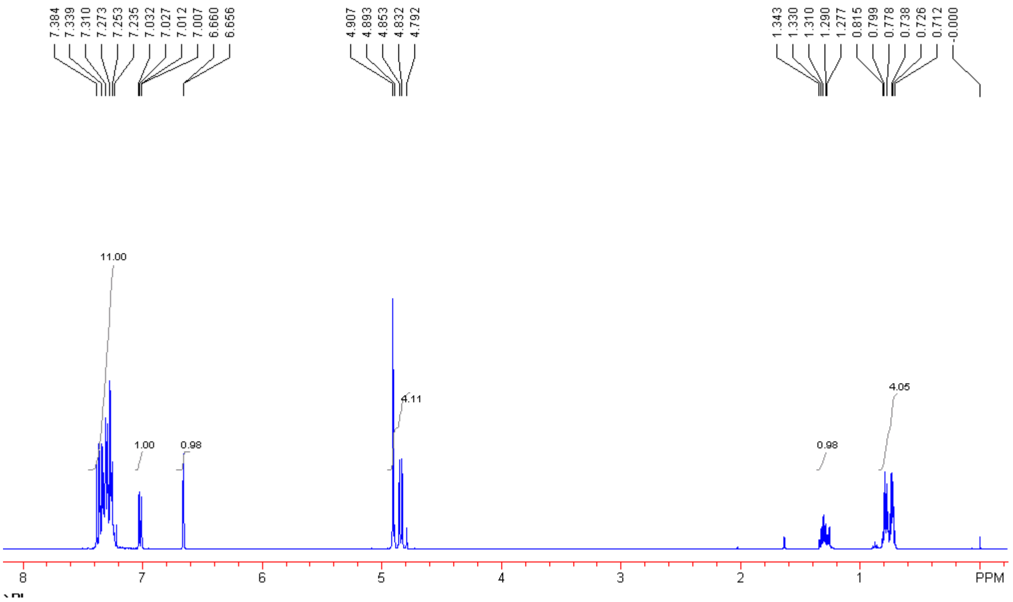

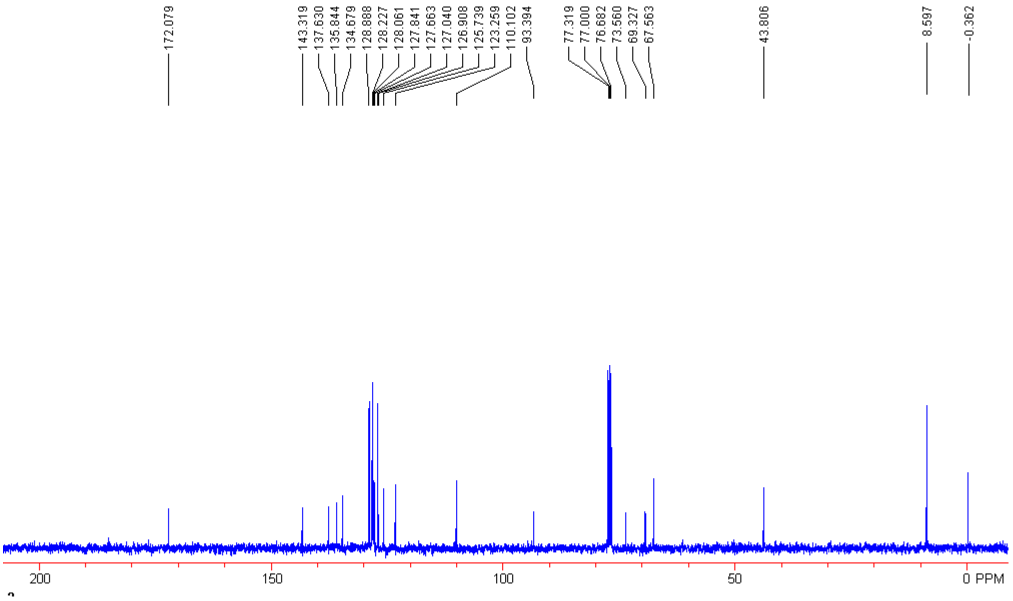
172.079
143.319
137.630
135.844
134.679
128.888
128.227
128.061
127.841
127.663
127.040
126.908
125.739
123.259
110.102
93.394
77.319
77.000
76.682
73.560
69.327
67.563
43.806
8.597
-0.362
200
150
100
50
0 PPM

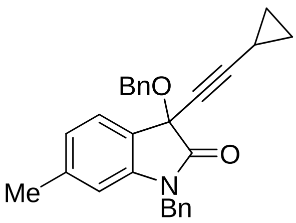

Compound 1l: A light yellow solid. Mp: 93-95 °C. IR (neat) ν 3033, 2882, 1720, 1621, 1454, 1023, 698 cm^{-1}. ^{1}H NMR ($CDCl_3$, 400 MHz, TMS) δ 0.69-0.71 (m, 4H), 1.22-1.29 (m, 1H), 2.20 (s, 3H), 4.75 (d, *J* = 16.0 Hz, 1H), 4.83 (d, *J* = 16.0 Hz, 1H), 4.88 (s, 2H), 6.47 (s, 1H), 6.82 (d, *J* = 8.4 Hz, 1H), 7.18-7.28 (m, 8H), 7.33-7.36 (m, 3H). ^{13}C NMR ($CDCl_3$, 100 MHz, TMS) δ -0.5, 8.3, 21.6, 43.3, 67.1, 70.0, 73.8, 92.4, 110.1, 123.7, 124.3, 125.3, 126.8, 127.3, 127.8, 127.9, 128.5, 135.2, 137.7, 140.4, 142.0, 172.2. MS (ESI) *m/z* 430.1 (M+Na)$^{+}$. HRMS (ESI) Calcd. for $C_{28}H_{25}NO_2Na$ (M+Na)$^{+}$: 430.1778, Found: 430.1775.

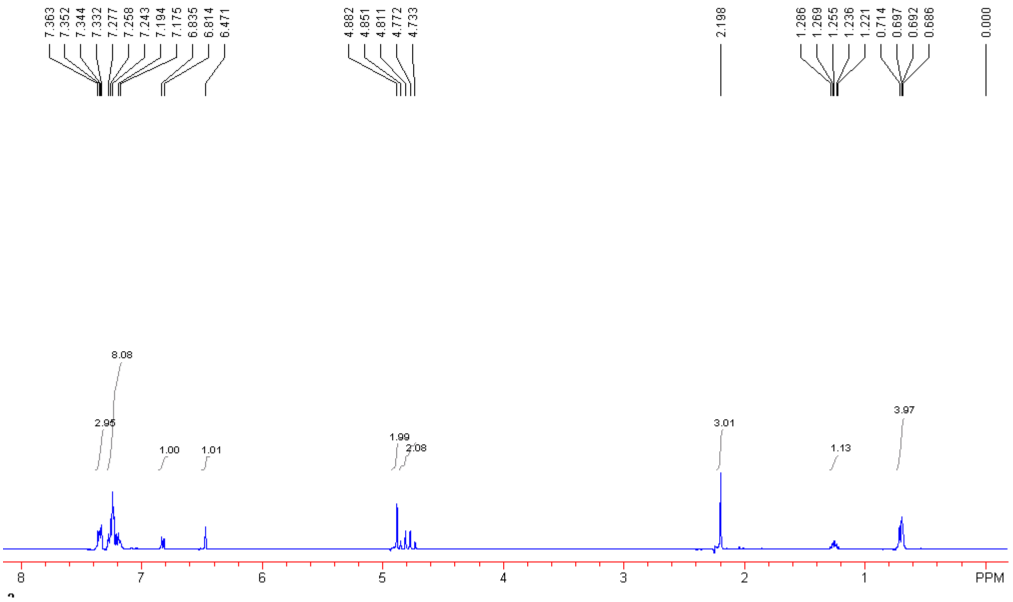

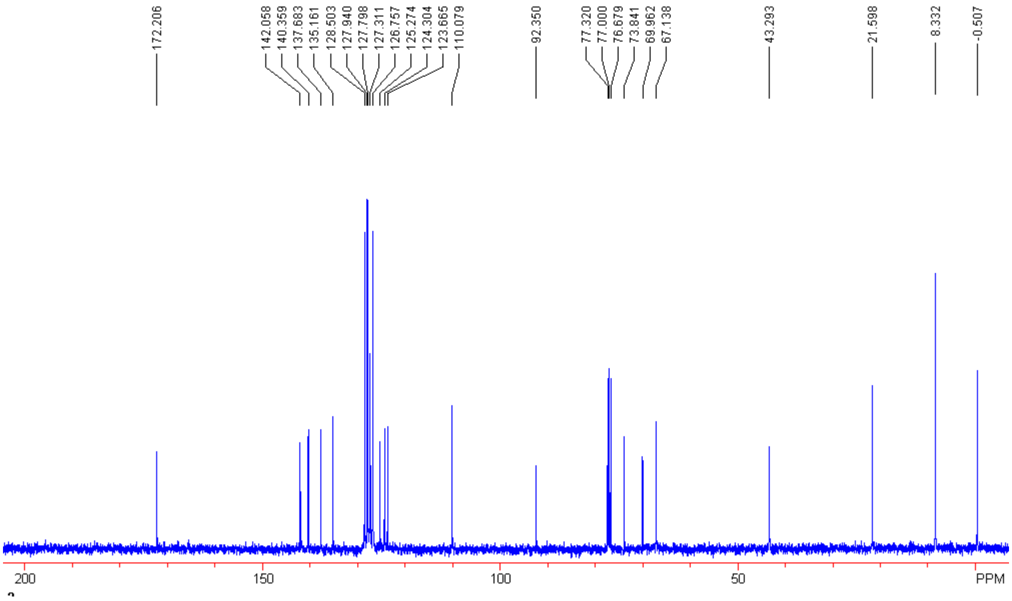

172.206
142.058
140.359
137.683
135.161
128.503
127.940
127.798
127.311
126.757
125.274
124.304
123.665
110.079
92.350
77.320
77.000
76.679
73.841
69.962
67.138
43.293
21.598
8.332
-0.507
200
150
100
50
PPM

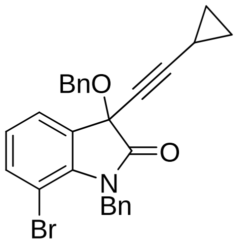

Compound 1m: A light yellow oil. IR (neat) ν 3030, 2918, 1728, 1449, 1119, 1023, 694 cm^{-1}. ^{1}H NMR (CDCl$_3$, 400 MHz, TMS) δ 0.71-0.81 (m, 4H), 1.28-1.34 (m, 1H), 4.94 (s, 2H), 5.37 (s, 2H), 6.92 (t, *J* = 8.0 Hz, 1H), 7.20-7.35 (m, 11H), 7.44 (d, *J* = 7.2 Hz, 1H). ^{13}C NMR (CDCl$_3$, 100 MHz, TMS) δ -0.4, 8.6, 44.4, 67.6, 69.4, 73.2, 93.6, 102.6, 124.1, 124.7, 126.1, 127.0, 127.6, 128.1, 128.2, 128.5, 131.7, 135.9, 136.8, 137.6, 139.6, 172.8. MS (ESI) *m/z* 489.1 (M+NH$_4$)$^+$. HRMS (ESI) Calcd. for $C_{27}H_{22}NO_2BrNa$ (M+Na)$^+$: 494.0726, Found: 494.0738.

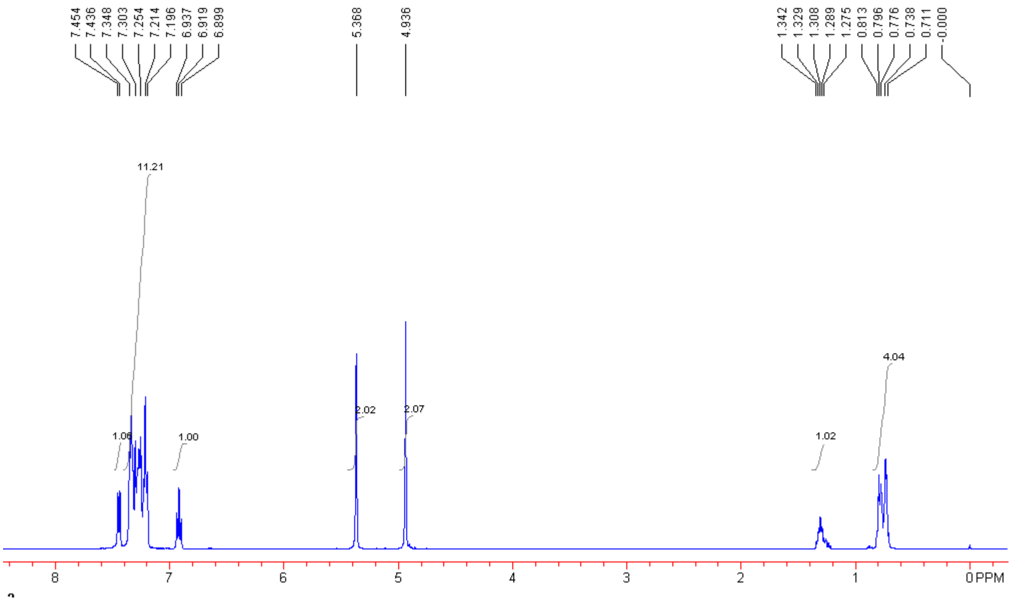

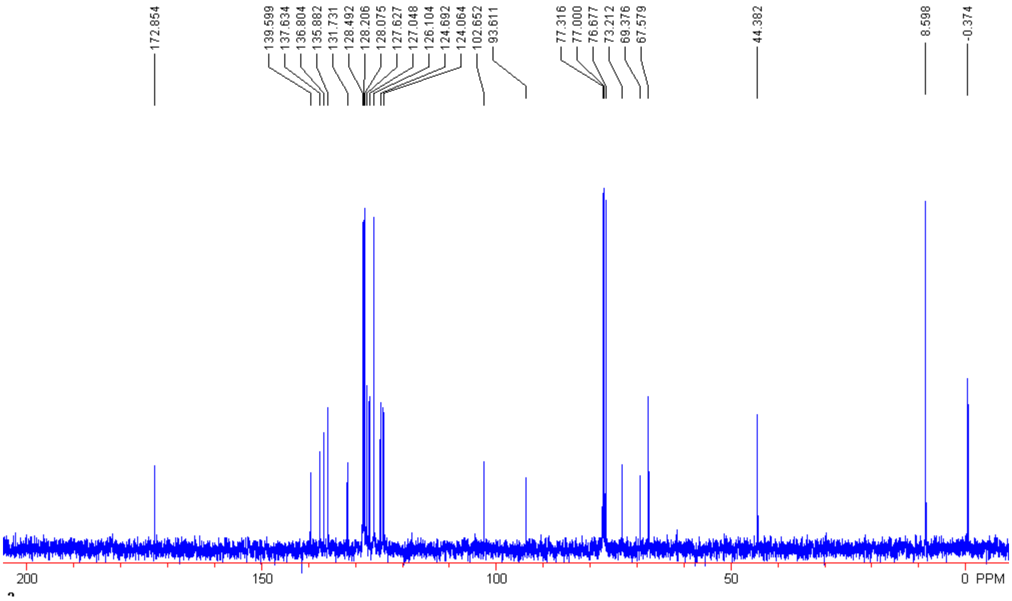
172.854
139.599
137.634
136.804
135.882
131.731
128.492
128.206
128.075
127.627
127.048
126.104
124.692
124.064
102.652
93.611
77.316
77.000
76.677
73.212
69.376
67.579
44.382
8.598
-0.374
200
150
100
50
0 PPM

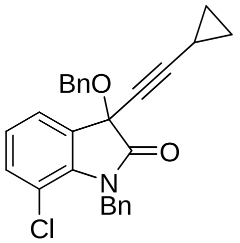

Compound 1n: A light yellow oil. IR (neat) ν 3031, 2930, 1728, 1452, 1129, 1026, 726 cm^{-1}. ^{1}H NMR ($CDCl_3$, 400 MHz, TMS) δ 0.70-0.80 (m, 4H), 1.28-1.33 (m, 1H), 4.94 (s, 2H), 5.30 (s, 2H), 6.96 (dd, J = 7.2 Hz, J = 8.4 Hz, 1H), 7.13 (dd, J = 1.2 Hz, J = 8.0 Hz, 1H), 7.18-7.35 (m, 10H), 7.39 (dd, J = 1.2 Hz, J = 7.2 Hz, 1H). ^{13}C NMR ($CDCl_3$, 100 MHz, TMS) δ -0.4, 8.6, 44.7, 67.5, 69.4, 73.3, 93.6, 115.6, 123.4, 124.3, 126.2, 127.1, 127.6, 128.0, 128.2, 128.5, 131.3, 132.5, 136.9, 137.6, 138.1, 172.6. MS (ESI) m/z 445.0 $(M+NH_4)^+$. HRMS (ESI) Calcd. for $C_{27}H_{22}NO_2ClNa$ $(M+Na)^+$: 450.1231, Found: 450.1232.

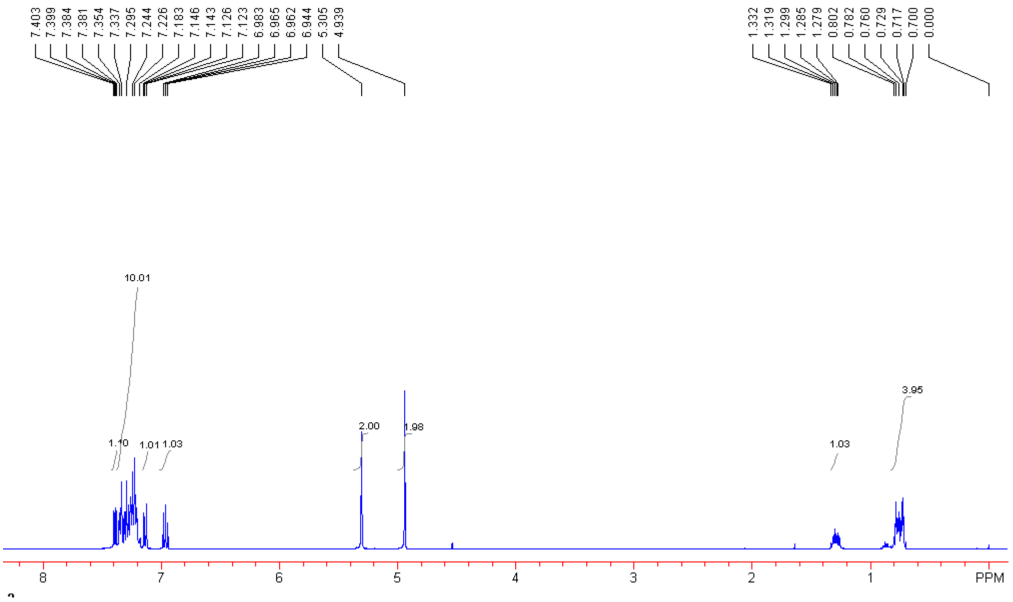

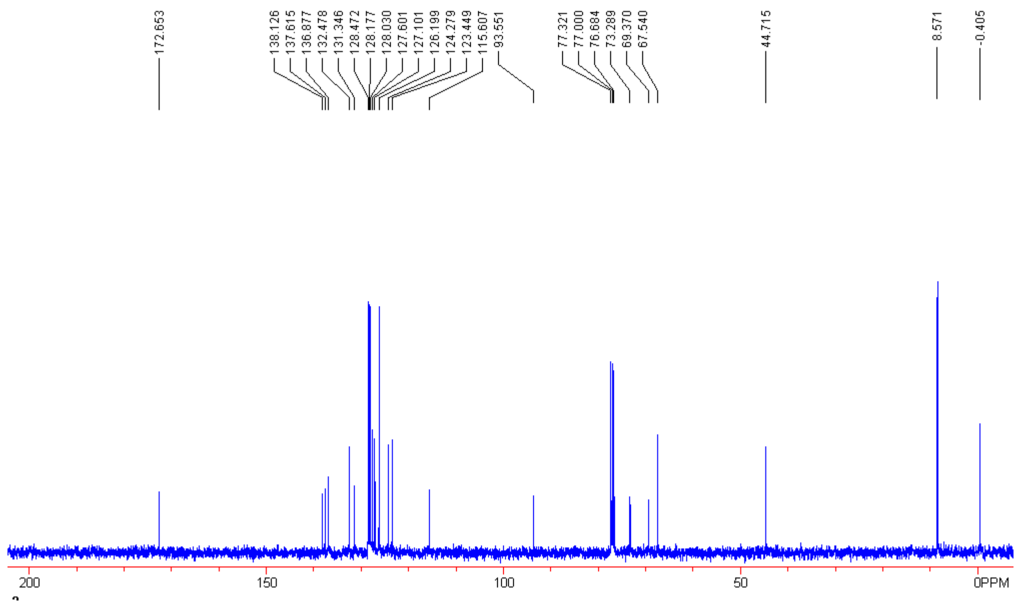
172.653
138.126
137.615
136.877
132.478
131.346
128.472
128.177
128.030
127.601
127.101
126.199
124.279
123.449
115.607
93.551
77.321
77.000
76.684
73.289
69.370
67.540
44.715
8.571
-0.405
200
150
100
50
0PPM

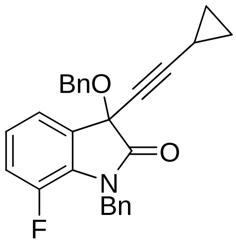

Compound 1o: A light yellow oil. IR (neat) ν 3032, 2931, 1726, 1488, 1341, 1168, 732 cm^{-1}. ^{1}H NMR ($CDCl_3$, 400 MHz, TMS) δ 0.70-0.78 (m, 4H), 1.26-1.30 (m, 1H), 4.90 (s, 2H), 4.87 (d, *J* = 15.2 Hz, 1H), 5.03 (d, *J* = 15.2 Hz, 1H), 6.90-6.97 (m, 2H), 7.19-7.34 (m, 11H). ^{13}C NMR ($CDCl_3$, 100 MHz, TMS) δ -0.4, 8.5, 45.2 (*J* = 5.1 Hz), 67.4, 69.4, 73.8 (*J* = 5.1 Hz), 93.4, 118.1 (*J* = 19.5 Hz), 120.6 (*J* = 3.1 Hz), 124.0 (*J* = 6.3 Hz), 127.1, 127.47, 127.55, 128.0, 128.1, 128.4, 128.7 (*J* = 8.7 Hz), 131.1 (*J* = 2.6 Hz), 136.4, 137.6, 147.2 (*J* = 244.2 Hz), 171.8. ^{19}F NMR ($CDCl_3$, 376 MHz, CF_3COOH) δ -133.11 ~ -133.07 (m). MS (ESI) *m/z* 434.0 $(M+Na)^+$. HRMS (ESI) Calcd. for $C_{27}H_{22}NO_2FNa$ $(M+Na)^+$: 434.1527, Found: 434.1529.

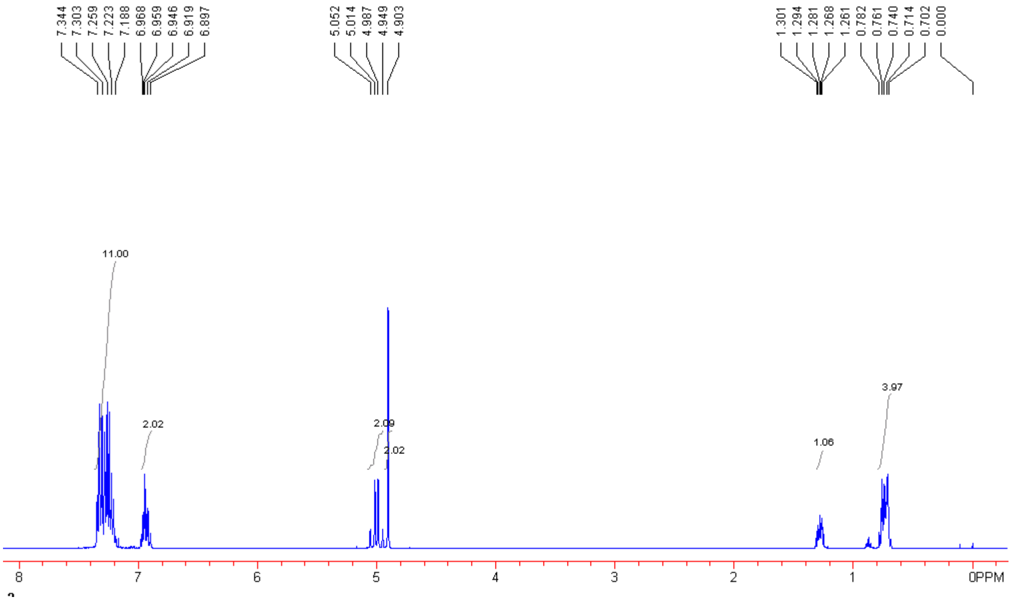

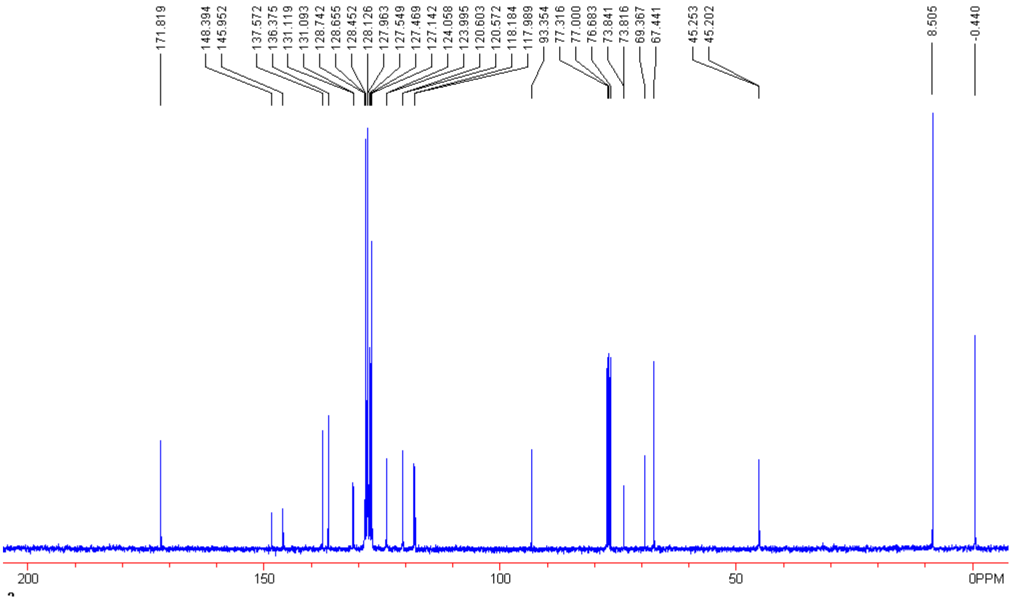
171.819
148.394
145.952
137.572
136.375
131.119
131.093
128.742
128.655
128.452
128.126
127.963
127.549
127.469
127.142
124.058
123.995
120.603
120.572
118.184
117.989
93.354
77.316
77.000
76.683
73.841
73.816
69.367
67.441
45.253
45.202
8.505
-0.440
200
150
100
50
0PPM

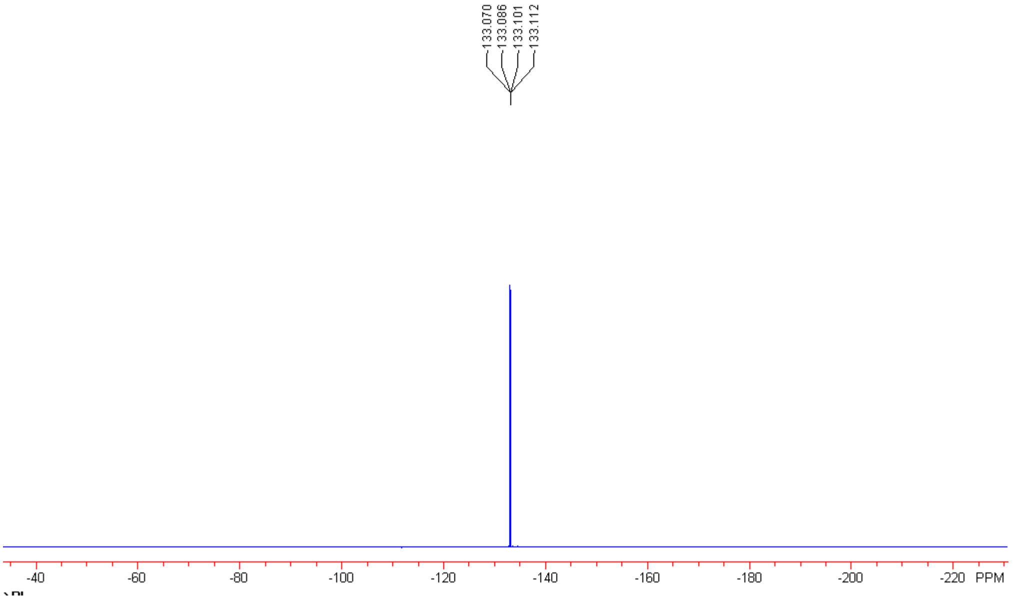
133.070
133.086
133.101
133.112
-40
-60
-80
-100
-120
-140
-160
-180
-200
-220
PPM

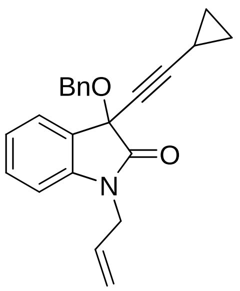

Compound 1p: A light yellow solid. Mp: 91-93 °C. IR (neat) ν 3012, 2929, 1715, 1471, 1371, 1021, 753 cm^{-1}. ^{1}H NMR ($CDCl_3$, 400 MHz, TMS) δ 0.68-0.77 (m, 4H), 1.22-1.29 (m, 1H), 4.23-4.26 (m, 2H), 4.86 (s, 2H), 5.16-5.22 (m, 2H), 5.73-5.82 (m, 1H), 6.74 (d, J = 7.6 Hz, 1H), 7.05 (t, J = 7.6 Hz, 1H), 7.20-7.34 (m, 6H), 7.46 (d, J = 7.6 Hz, 1H). ^{13}C NMR ($CDCl_3$, 100 MHz, TMS) δ -0.6, 8.3, 42.0, 67.1, 69.7, 73.8, 92.4, 109.2, 117.3, 123.0, 124.4, 127.3, 127.8, 127.9, 128.0, 129.9, 130.6, 137.5, 141.9, 171.4. MS (ESI) *m/z* 366.1 $(M+Na)^+$. HRMS (ESI) Calcd. for $C_{23}H_{21}NO_2Na$ $(M+Na)^+$: 366.1464, Found: 366.1452.

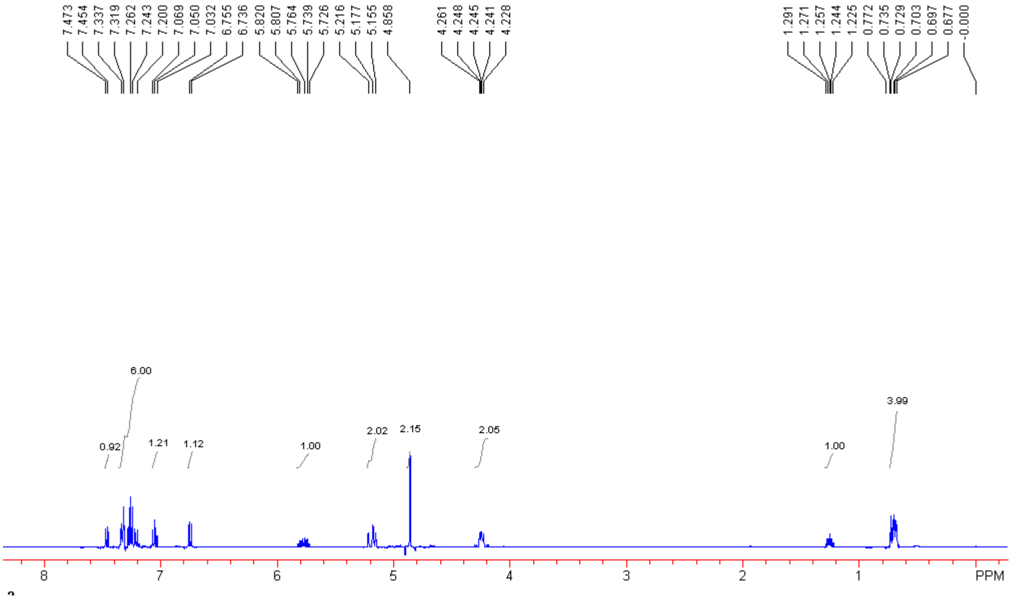

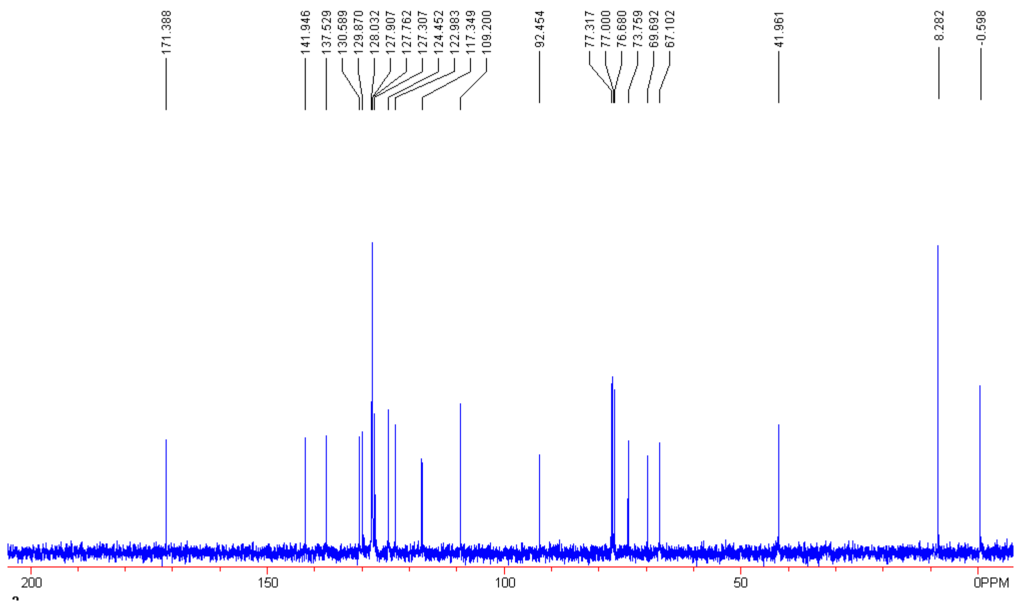
171.388
141.946
137.529
130.589
129.870
128.032
127.907
127.762
127.307
124.452
122.983
117.349
109.200
92.454
77.317
77.000
76.680
73.759
69.692
67.102
41.961
8.282
-0.598
200
150
100
50
0PPM

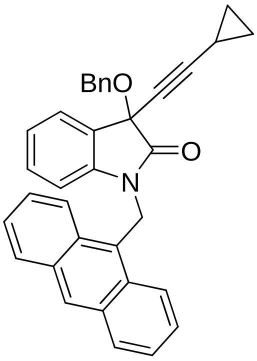

Compound 1q: A light yellow solid. Mp: 79-81 °C. IR (neat) ν 3031, 2932, 1730, 1451, 1163, 1121, 736 cm^{-1}. ^{1}H NMR ($CDCl_3$, 400 MHz, TMS) δ 0.70-0.72 (m, 3H), 0.84-0.87 (m, 1H), 1.28-1.30 (m, 1H), 4.85 (d, J = 10.8 Hz, 1H), 4.90 (d, J = 10.8 Hz, 1H), 5.79 (s, 2H), 6.16 (d, J = 8.0 Hz, 1H), 6.66 (t, J = 8.0 Hz, 1H), 6.75 (t, J = 7.6 Hz, 1H), 7.23-7.40 (m, 8H), 7.45-7.49 (m, 2H), 7.91 (d, J = 8.4 Hz, 2H), 8.34 (d, J = 8.0 Hz, 3H). ^{13}C NMR ($CDCl_3$, 100 MHz, TMS) δ -0.4, 8.4, 37.5, 67.5, 70.2, 74.0, 92.4, 110.4, 122.8, 123.4, 124.3, 124.9, 125.3, 126.9, 127.5, 127.9, 128.1, 128.8, 129.4, 129.8, 130.6, 131.1, 137.8, 142.1, 172.1. MS (ESI) m/z 516.1 $(M+Na)^{+}$. HRMS (ESI) Calcd. for $C_{35}H_{27}NO_2Na$ $(M+Na)^{+}$: 516.1934, Found: 516.1936.

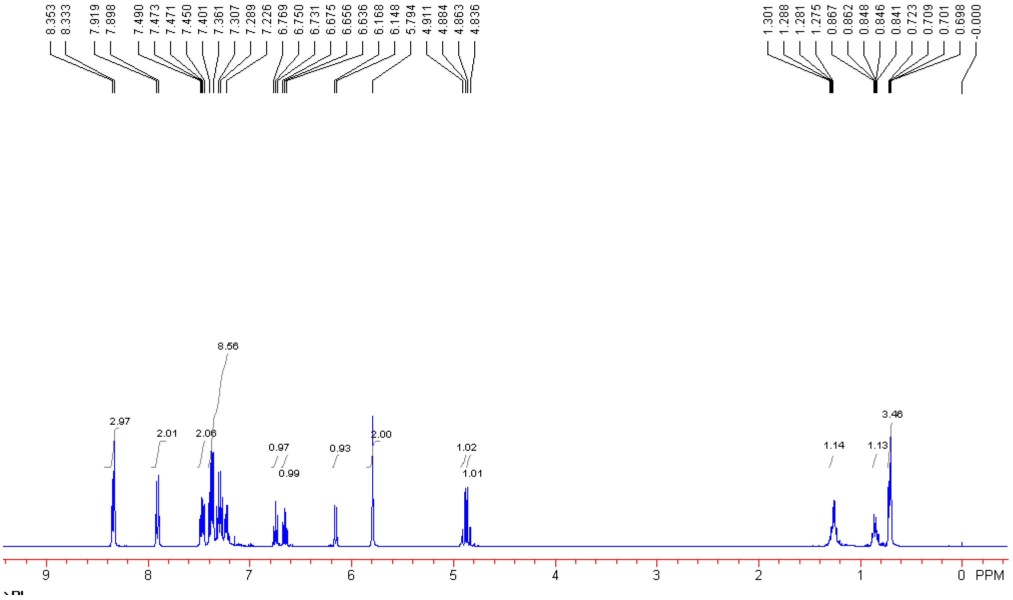

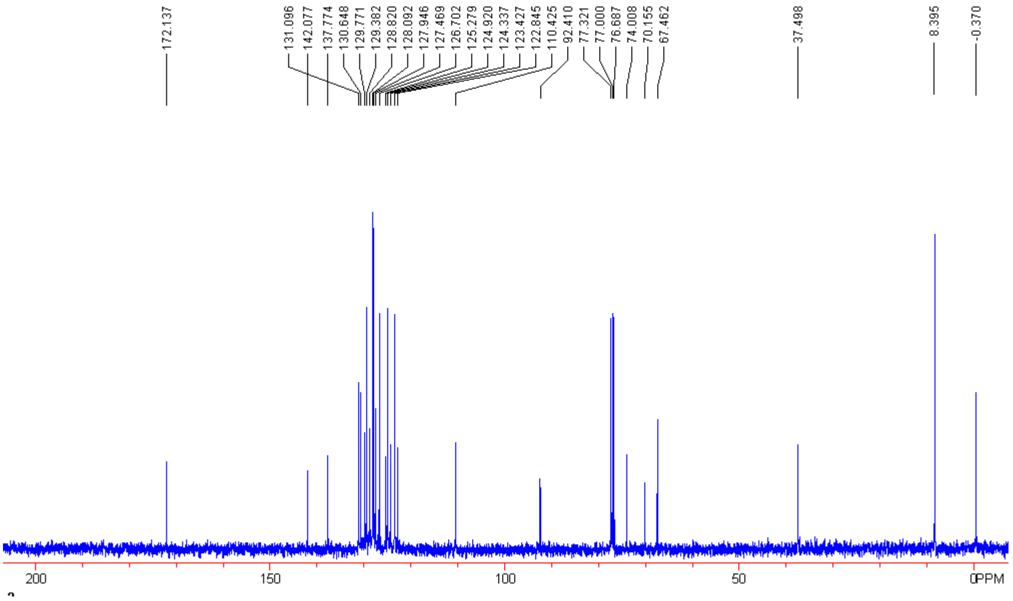
172.137
131.096
142.077
137.774
130.648
129.771
129.382
128.820
128.092
127.946
127.469
126.702
125.279
124.920
124.337
123.427
122.845
110.425
92.410
77.321
77.000
76.687
74.008
70.155
67.462
37.498
8.395
-0.370
200
150
100
50
0PPM

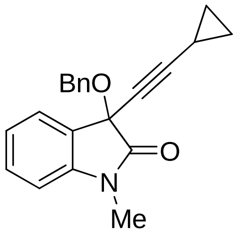

Compound 1r: A light yellow oil. IR (neat) ν 2925, 2855, 1721, 1610, 1469, 1344, 1020, 751 cm^{-1}. ^{1}H NMR ($CDCl_3$, 400 MHz, TMS) δ 0.70-0.77 (m, 4H), 1.28-1.31 (m, 1H), 3.15 (s, 3H), 4.84 (s, 2H), 6.77 (d, J = 8.0 Hz, 1H), 7.08 (dt, J = 0.8 Hz, J = 7.6 Hz, 1H), 7.20-7.34 (m, 6H), 7.46 (dd, J = 1.2 Hz, J = 7.6 Hz, 1H). ^{13}C NMR ($CDCl_3$, 100 MHz, TMS) δ -0.4, 8.4, 26.2, 67.3, 69.8, 74.0, 92.6, 108.5, 123.2, 124.6, 127.5, 128.0, 128.1, 128.2, 130.1, 137.7, 143.0, 171.9. MS (ESI) m/z 335.1 $(M+NH_4)^+$. HRMS (ESI) Calcd. for $C_{21}H_{19}NO_2Na$ $(M+Na)^+$: 340.1308, Found: 340.1306.

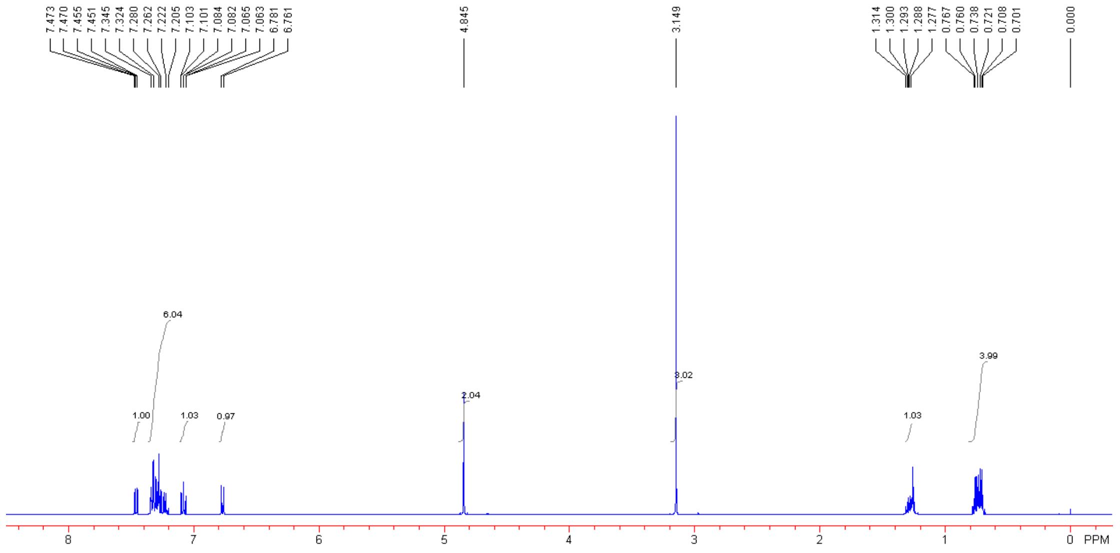

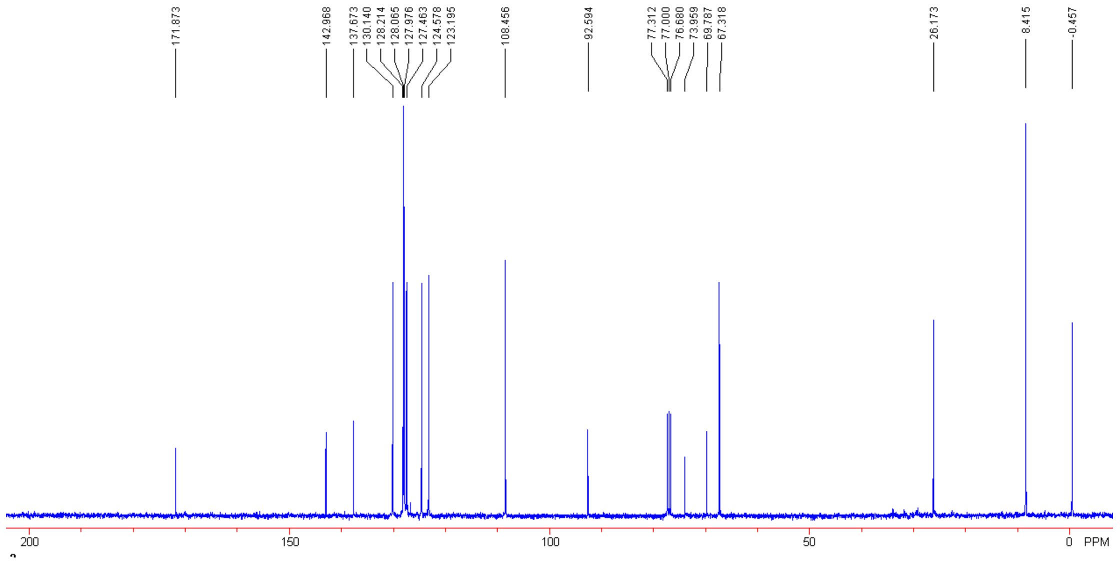

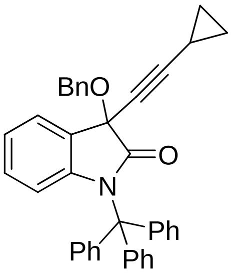

Compound 1s: A light yellow solid. Mp: 82-84 °C. IR (neat) ν 3010, 2922, 1737, 1468, 1028, 746 cm^{-1}. ^{1}H NMR ($CDCl_3$, 400 MHz, TMS) δ 0.73-0.79 (m, 4H), 1.28-1.32 (m, 1H), 4.76 (s, 2H), 6.09 (d, J = 8.4 Hz, 1H), 6.98 (d, J = 8.8 Hz, 1H), 7.17-7.29 (m, 15H), 7.43 (d, J = 7.6 Hz, 6H), 7.51 (s, 1H). ^{13}C NMR ($CDCl_3$, 100 MHz, TMS) δ -0.3, 8.7, 67.8, 69.4, 73.7, 74.2, 93.5, 115.6, 117.2, 127.0, 127.1, 127.5, 127.7, 127.8, 127.9, 128.2, 128.9, 130.7, 131.3, 137.8, 141.3, 141.7, 172.8.

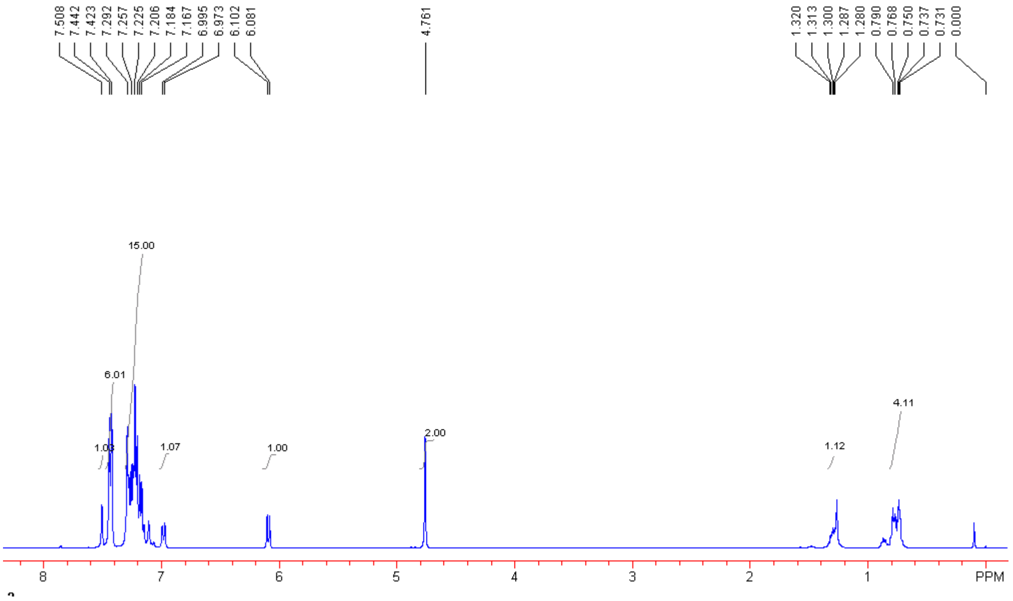

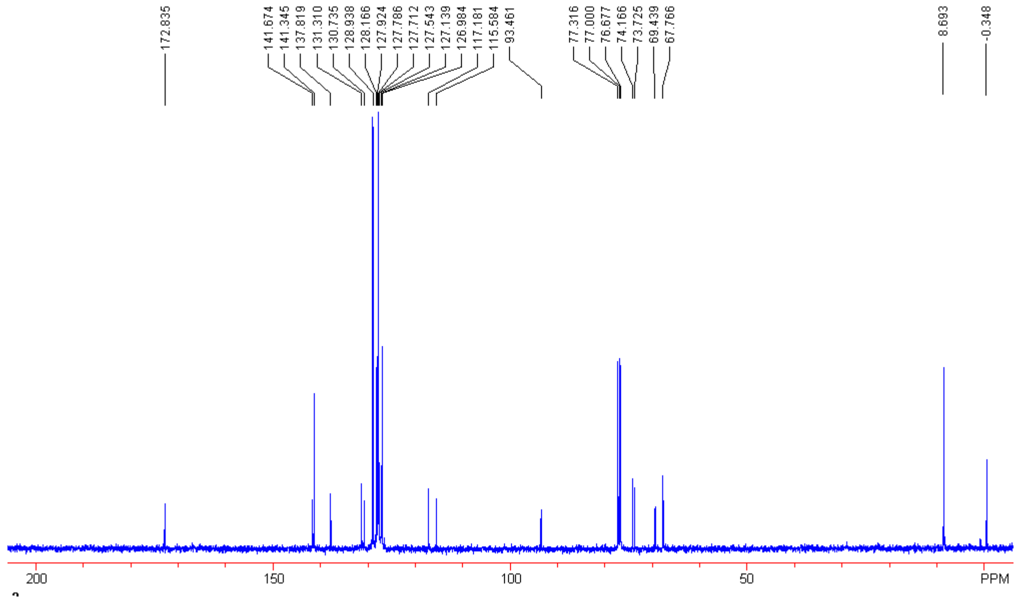
172.835
141.674
141.345
137.819
131.310
130.735
128.938
128.166
127.924
127.786
127.712
127.543
127.139
126.984
117.181
115.584
93.461
77.316
77.000
76.677
74.166
73.725
69.439
67.766
8.693
-0.348
200
150
100
50
PPM

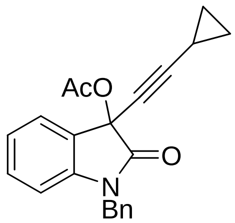

Compound 1t: A white solid. Mp: 117-119 °C. IR (neat) ν 3013, 2928, 1733, 1612, 1369, 1233, 952, 756 cm^{-1}. ^{1}H NMR ($CDCl_3$, 400 MHz, TMS) δ 0.71-0.77 (m, 4H), 1.24-1.28 (m, 1H), 2.07 (s, 3H), 4.84 (d, *J* = 16.0 Hz, 1H), 4.99 (d, *J* = 16.0 Hz, 1H), 6.61 (d, *J* = 8.0 Hz, 1H), 7.00 (dt, *J* = 1.2 Hz, *J* = 7.6 Hz, 1H), 7.14 (dt, *J* = 1.2 Hz, *J* = 7.6 Hz, 1H), 7.21-7.38 (m, 6H). ^{13}C NMR ($CDCl_3$, 100 MHz, TMS) δ -0.5, 8.39, 8.43, 20.5, 44.1, 68.6, 73.3, 92.5, 109.7, 123.0, 127.0, 127.4, 128.5, 129.9, 135.0, 142.2, 168.4, 170.7. MS (ESI) *m/z* 368.0 $(M+Na)^+$. HRMS (ESI) Calcd. for $C_{22}H_{19}NO_3Na$ $(M+Na)^+$: 368.1257, Found: 368.1254.

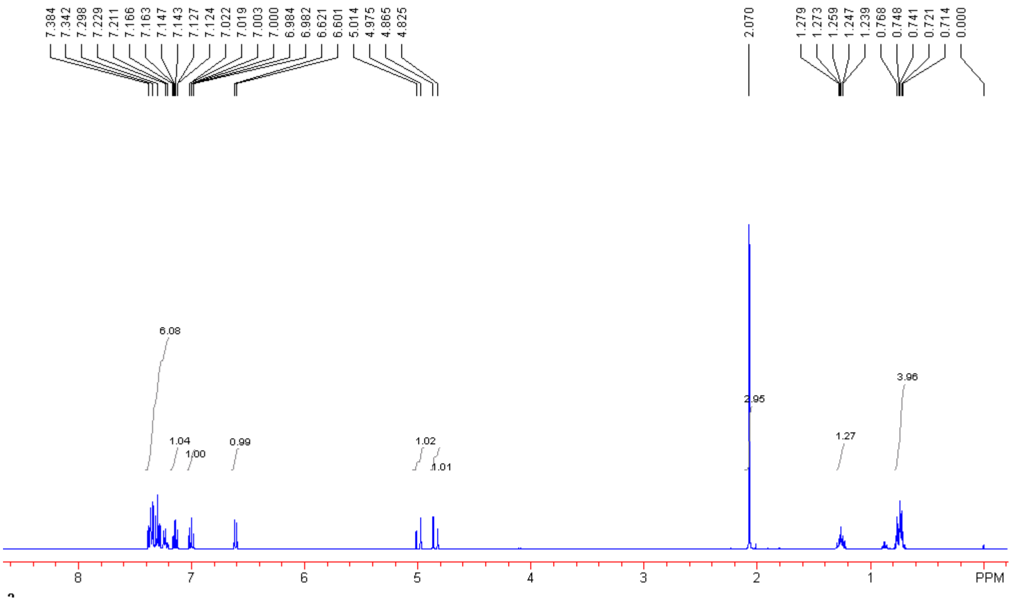

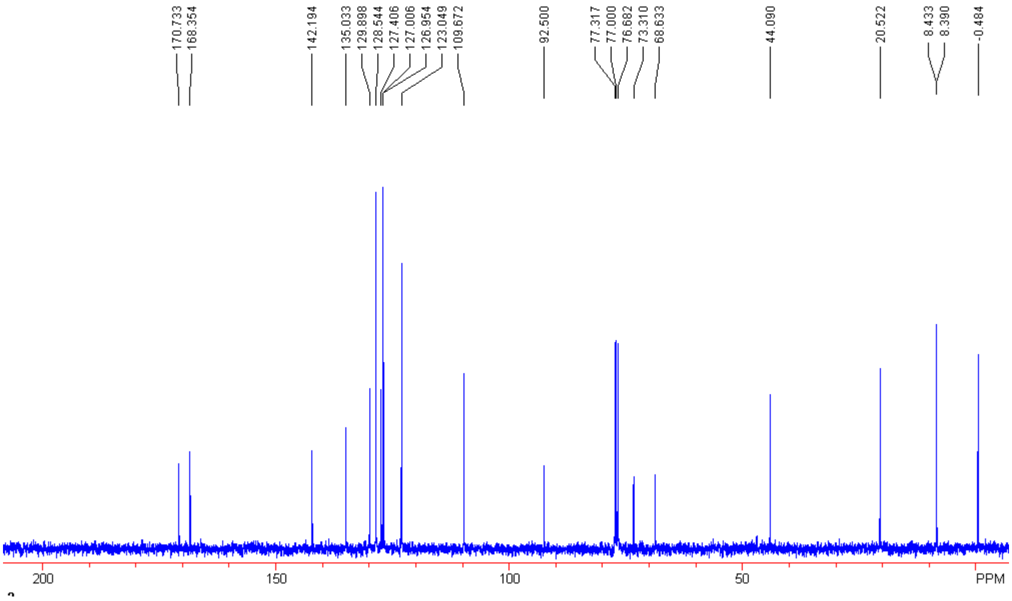

170.733
168.354
142.194
135.033
129.898
128.544
127.406
127.006
126.954
123.049
109.672
92.500
77.317
77.000
76.682
73.310
68.633
44.090
20.522
8.433
8.390
-0.484
200
150
100
50
PPM

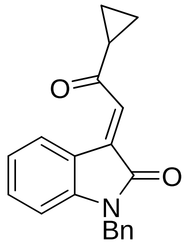

Compound 2a: Yield: 16 mg. 67%. A red solid. Mp: 89-91 °C. IR (neat) ν 3025, 2924, 1715, 1667, 1466, 1354, 1117, 718 cm^{-1}. ^{1}H NMR ($CDCl_3$, 400 MHz, TMS) δ 1.07-1.12 (m, 2H), 1.24-1.28 (m, 2H), 2.29-2.35 (m, 1H), 4.94 (s, 2H), 6.68 (d, *J* = 8.0 Hz, 1H), 6.99 (dt, *J* = 0.8 Hz, *J* = 8.0 Hz, 1H), 7.22-7.33 (m, 6H), 7.40 (s, 1H), 8.49 (d, *J* = 7.6 Hz, 1H). ^{13}C NMR ($CDCl_3$, 75 MHz, TMS) δ 12.8, 23.4, 43.8, 109.1, 120.3, 122.8, 127.2, 127.7, 128.1, 128.4, 128.8, 132.6, 134.3, 135.4, 145.1, 168.4, 200.9. MS (ESI) *m/z* 304.1 $(M+H)^+$. HRMS (ESI) Calcd. for $C_{20}H_{18}NO_2$ $(M+H)^+$: 304.1332, Found: 304.1333.

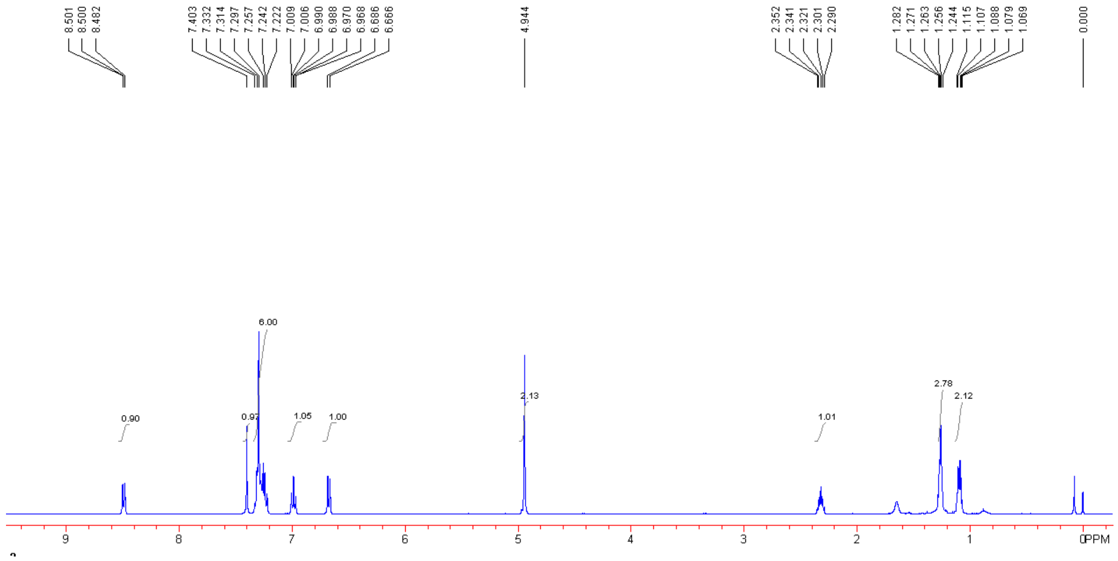

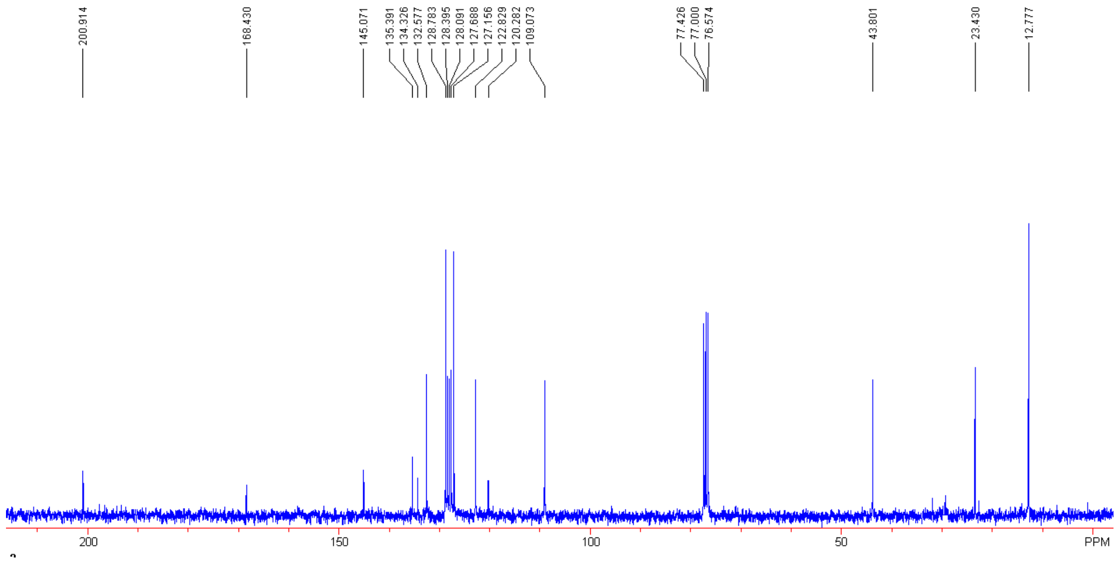

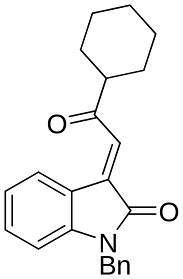

Compound 2b: Yield: 119 mg. 50%. A red solid. Mp: 117-119 °C. IR (neat) ν 2927, 1714, 1678, 1356, 1188, 751 cm^{-1}. ^1H NMR ($CDCl_3$, 400 MHz, TMS) δ 1.25-1.46 (m, 5H), 1.69-1.72 (m, 1H), 1.81-1.85 (m, 2H), 1.98-2.01 (m, 2H), 2.63-2.70 (m, 1H), 4.92 (s, 2H), 6.67 (d, J = 8.0 Hz, 1H), 6.99 (t, J = 8.0 Hz, 1H), 7.21-7.29 (m, 6H), 7.33 (s, 1H), 8.51 (d, J = 7.2 Hz, 1H). ^{13}C NMR ($CDCl_3$, 100 MHz, TMS) δ 25.4, 25.7, 28.1, 43.7, 52.0, 109.0, 120.2, 122.7, 127.1, 127.60, 127.65, 127.9, 128.7, 132.5, 135.35, 135.38, 144.9, 168.2, 204.1. HRMS (ESI) Calcd. for $C_{23}H_{23}NO_2Na$ $(M+Na)^+$: 368.1621, Found: 368.1617.

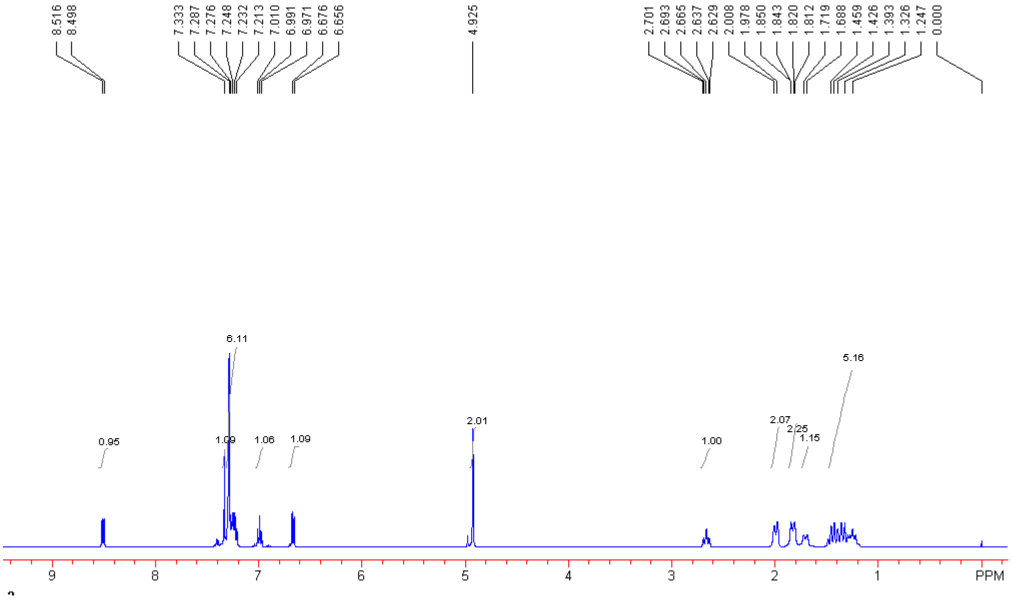

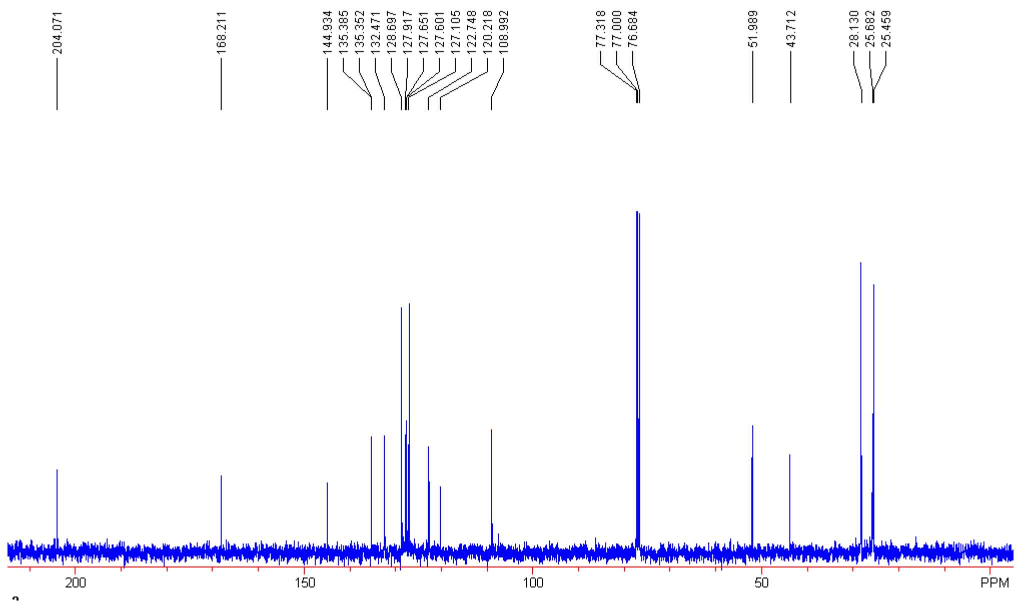
204.071
168.211
144.934
135.385
135.352
132.471
128.697
127.917
127.651
127.601
127.105
122.748
120.218
108.992
77.318
77.000
76.684
51.989
43.712
28.130
25.682
25.459
200
150
100
50
PPM

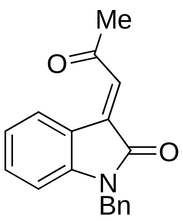

Compound 2c: Yield: 30 mg. 45%. A red oil. IR (neat) ν 2922, 1710, 1638, 1467, 1022, 750 cm^{-1}. ^{1}H NMR ($CDCl_3$, 300 MHz, TMS) δ 2.50 (s, 3H), 4.94 (s, 2H), 6.68 (d, *J* = 7.8 Hz, 1H), 7.01 (t, *J* = 7.8 Hz, 1H), 7.24-7.32 (m, 7H), 8.52 (d, *J* = 7.2 Hz, 1H). ^{13}C NMR ($CDCl_3$, 75 MHz, TMS) δ 32.3, 43.8, 109.1, 120.2, 122.9, 127.2, 127.7, 128.0, 128.2, 128.8, 132.8, 135.1, 135.3, 145.2, 168.3, 198.5. MS (ESI) *m/z* 300.1 $(M+Na)^+$. HRMS (ESI) Calcd. for $C_{18}H_{15}NO_2Na$ $(M+Na)^+$: 300.0995, Found: 300.0996.

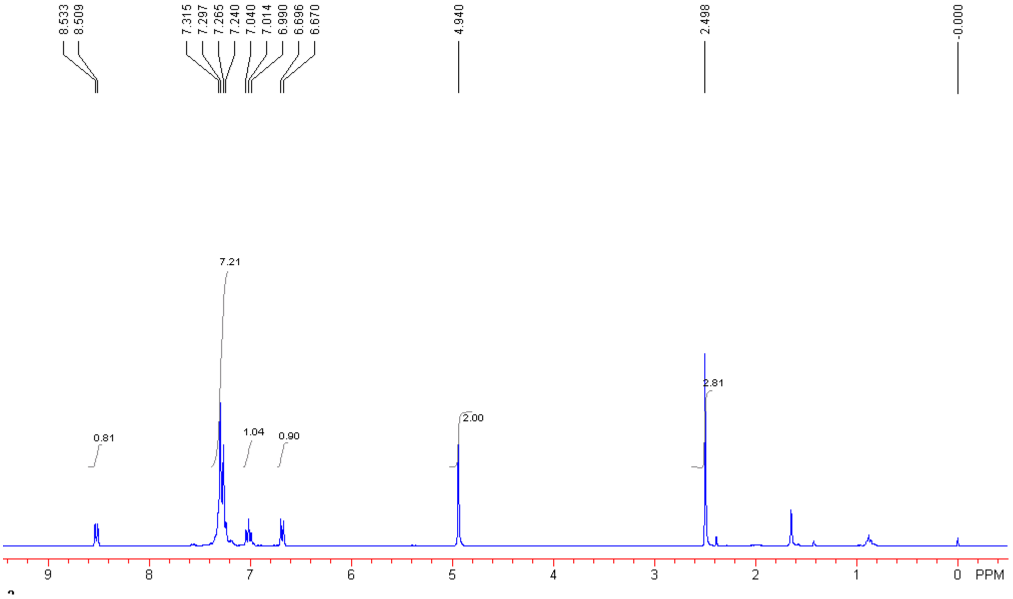

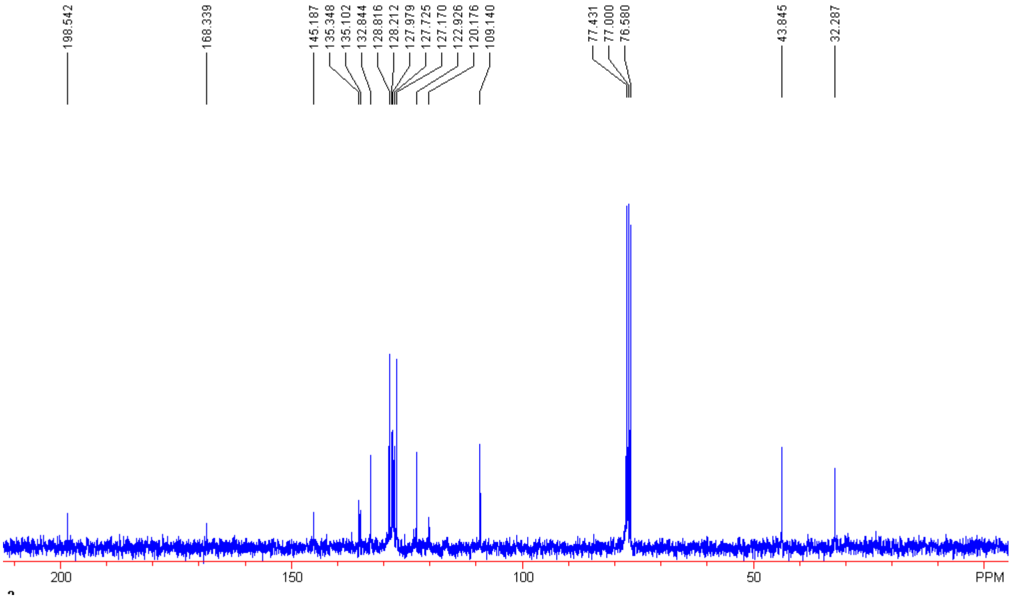

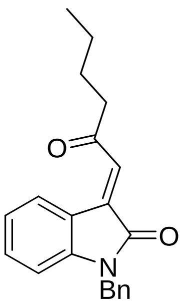

Compound 2d: Yield: 72 mg. 46%. A yellow solid. Mp: 68-70 ^{o}C. IR (neat) ν 2926, 2856, 1708, 1682, 1464, 1342, 782 cm^{-1}. ^{1}H NMR ($CDCl_3$, 400 MHz, TMS) δ 0.94 (t, *J* = 7.6 Hz, 3H), 1.34-1.43 (m, 2H), 1.66-1.74 (m, 2H), 2.74 (t, *J* = 7.6 Hz, 2H), 4.92 (s, 2H), 6.66 (d, *J* = 7.6 Hz, 1H), 6.99 (t, *J* = 7.2 Hz, 1H), 7.21-7.30 (m, 7H), 8.54 (d, *J* = 7.6 Hz, 1H). ^{13}C NMR ($CDCl_3$, 100 MHz, TMS) δ 13.8, 22.2, 25.9, 43.7, 44.7, 109.0, 120.1, 122.8, 127.1, 127.6, 127.9, 128.0, 128.7, 132.6, 134.9, 135.3, 145.0, 168.2, 201.2. MS (ESI) *m/z* 320.2 (M+H)$^{+}$. HRMS (ESI) Calcd. for $C_{21}H_{22}NO_2$ (M+H)$^{+}$: 320.1645, Found: 320.1638.

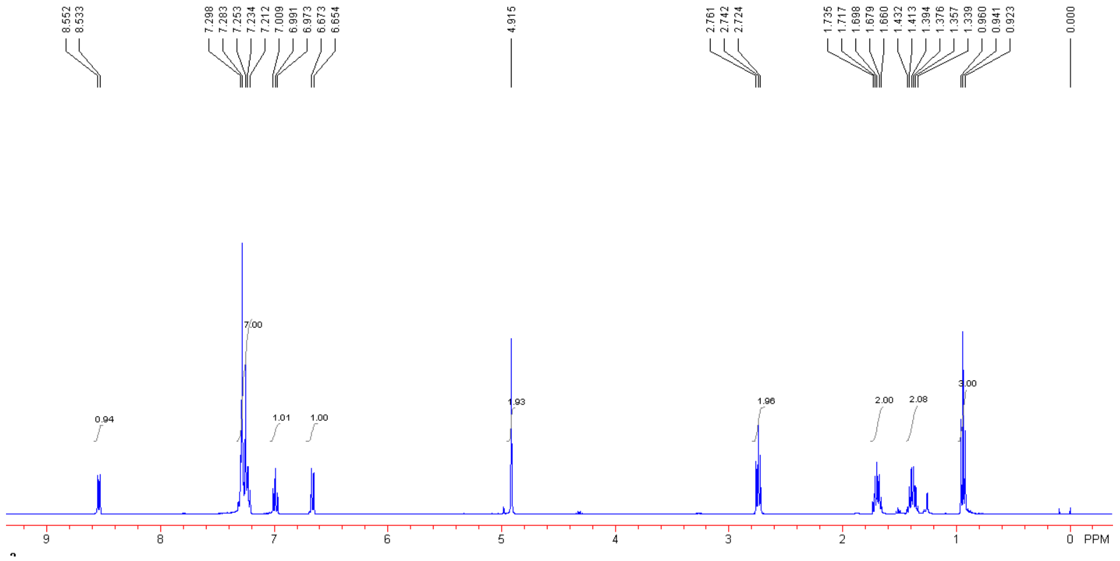

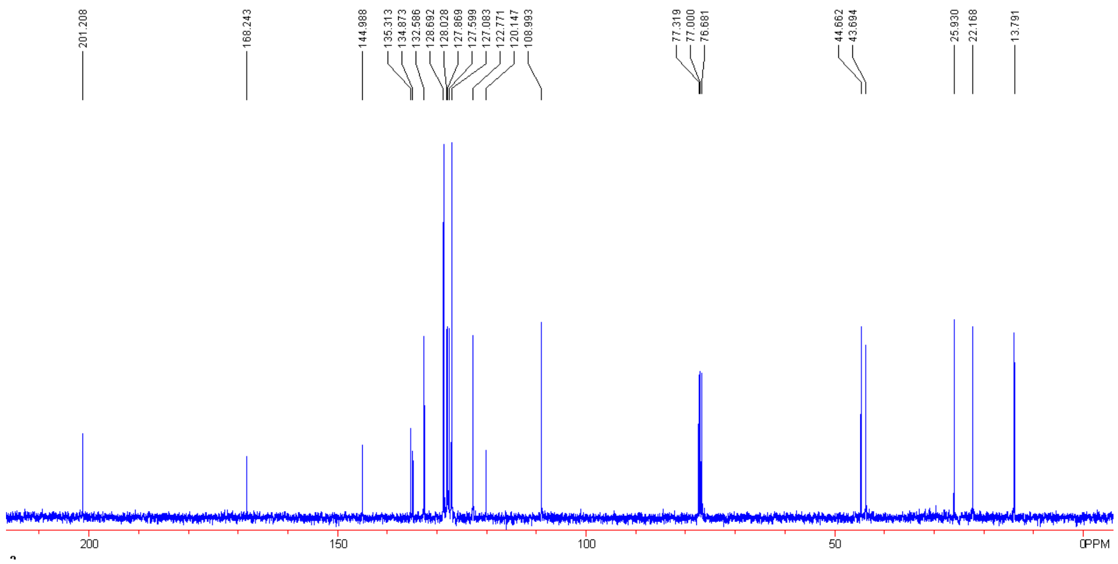

201.208
168.243
144.988
135.313
134.873
132.586
128.692
128.028
127.869
127.599
127.083
122.771
120.147
108.993
77.319
77.000
76.681
44.662
43.694
25.930
22.168
13.791
200
150
100
50
0PPM

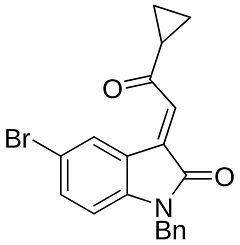

Compound 2e: Yield: 101 mg. 60%. A red solid. Mp: 135-137 °C. IR (neat) ν 2994, 2929, 1712, 1671, 1276, 1181, 750 cm^{-1}. ^{1}H NMR ($CDCl_3$, 400 MHz, TMS) δ 1.08-1.13 (m, 2H), 1.26-1.28 (m, 2H), 2.28-2.34 (m, 1H), 4.90 (s, 2H), 6.52 (d, J = 8.0 Hz, 1H), 7.26-7.33 (m, 6H), 7.40 (s, 1H), 8.67 (s, 1H). ^{13}C NMR ($CDCl_3$, 100 MHz, TMS) δ 13.0, 23.5, 43.7, 110.4, 115.4, 121.7, 127.0, 127.7, 128.8, 129.4, 130.8, 133.2, 134.86, 134.92, 143.8, 167.7, 200.4. MS (ESI) *m/z* 382.0 $(M+H)^+$. HRMS (ESI) Calcd. for $C_{20}H_{17}NO_2Br$ $(M+H)^+$: 382.0437, Found: 382.0423.

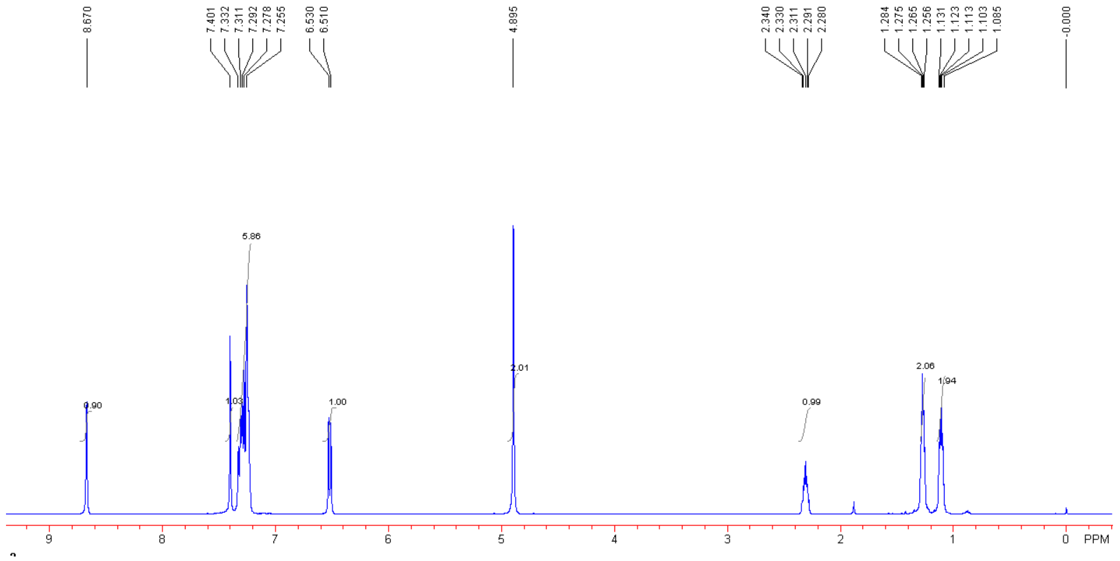

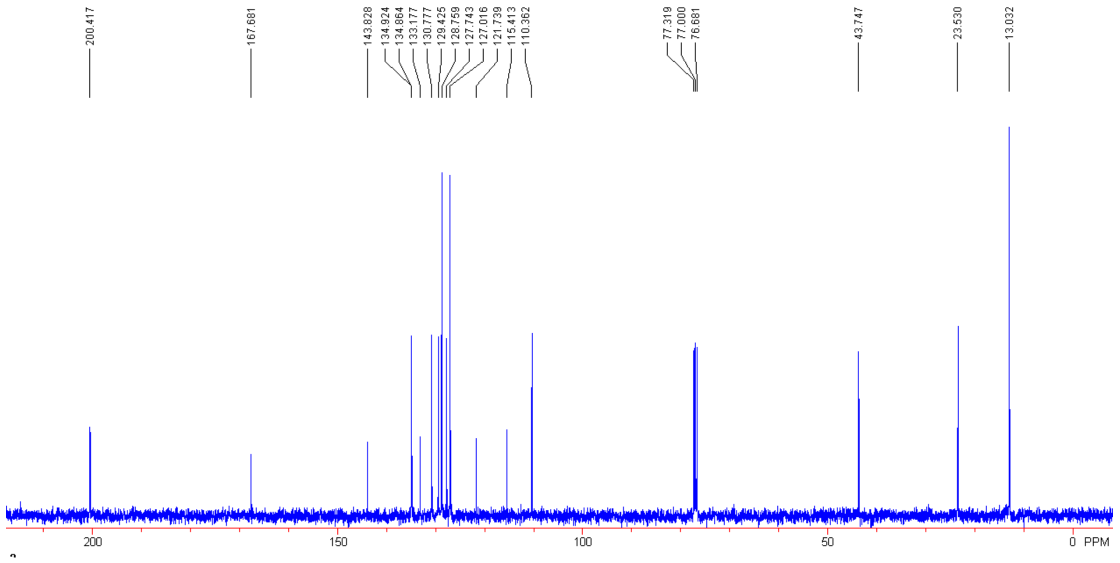

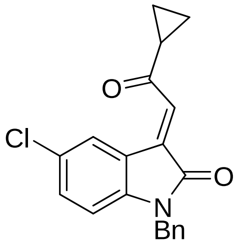

Compound 2f: Yield: 92 mg. 57%. A yellow solid. Mp: 120-122 °C. IR (neat) ν 2924, 2853, 1714, 1672, 1436, 1330, 1112, 805 cm^{-1}. ^{1}H NMR ($CDCl_3$, 400 MHz, TMS) δ 1.09-1.14 (m, 2H), 1.26-1.30 (m, 2H), 2.29-2.35 (m, 1H), 4.91 (s, 2H), 6.57 (d, *J* = 8.4 Hz, 1H), 7.19 (dd, *J* = 2.0 Hz, *J* = 8.4 Hz, 1H), 7.24-7.30 (m, 5H), 7.42 (s, 1H), 8.54 (d, *J* = 2.0 Hz, 1H). ^{13}C NMR ($CDCl_3$, 100 MHz, TMS) δ 13.0, 23.5, 43.8, 109.9, 121.4, 127.1, 127.8, 128.1, 128.2, 128.8, 129.5, 132.1, 133.4, 134.9, 143.4, 167.9, 200.5. MS (ESI) *m/z* 338.0 (M+H)$^+$. HRMS (ESI) Calcd. for $C_{20}H_{17}ClNO_2$ (M+H)$^+$: 338.0942, Found: 338.0941.

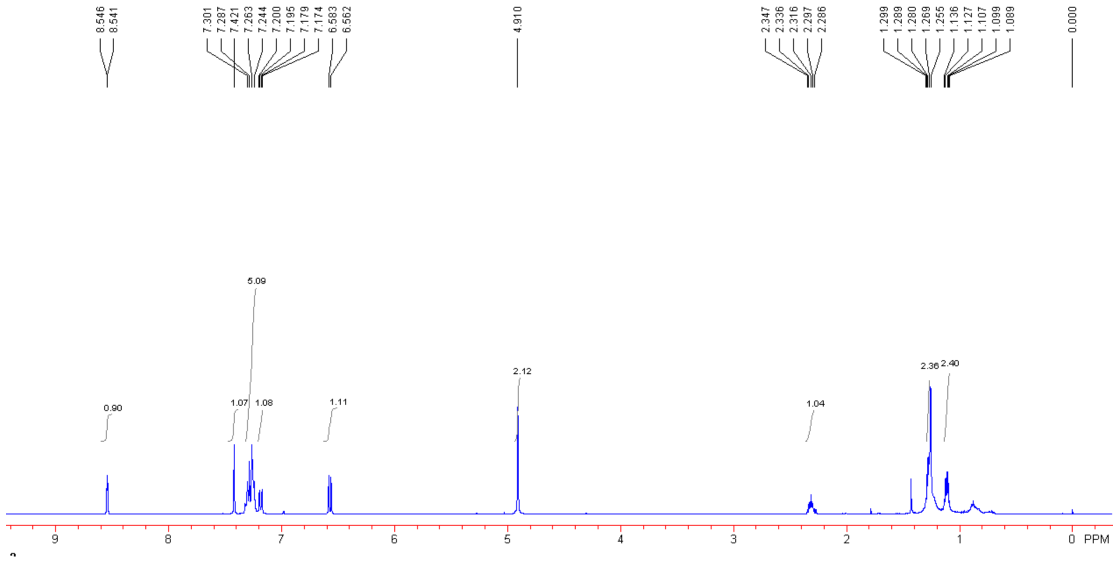

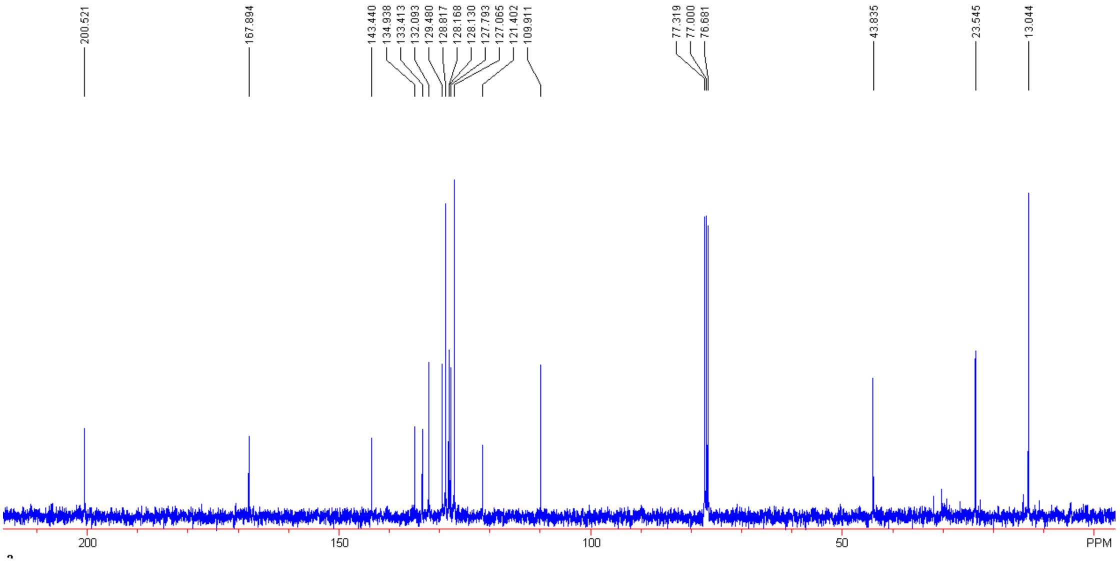

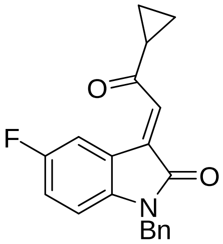

Compound 2g: Yield: 103 mg. 48%. A red solid. Mp: 126-128 °C. IR (neat) ν 3013, 2920, 1710, 1673, 1471, 1339, 1118, 695 cm^{-1}. ^{1}H NMR ($CDCl_3$, 400 MHz, TMS) δ 1.08-1.12 (m, 2H), 1.25-1.28 (m, 2H), 2.28-2.34 (m, 1H), 4.90 (s, 2H), 6.56 (dd, J = 4.0 Hz, J = 8.8 Hz, 1H), 6.92 (dd, J = 8.8 Hz, J = 11.2 Hz, 1H), 7.24-7.32 (m, 5H), 7.42 (s, 1H), 8.32 (dd, J = 2.4 Hz, J = 8.8 Hz, 1H). ^{13}C NMR ($CDCl_3$, 100 MHz, TMS) δ 12.9, 23.4, 43.8, 109.4 (d, J = 8.2 Hz), 115.6 (d, J = 26.8 Hz), 118.7 (d, J = 24.2 Hz), 121.1 (d, J = 9.7 Hz), 127.0, 127.7, 128.7, 129.3, 133.9 (d, J = 2.6 Hz), 135.0, 141.1, 158.7 (d, J = 238.4 Hz), 168.0, 200.5. ^{19}F NMR ($CDCl_3$, 376 MHz, CF_3COOH) δ -119.93 ~ -119.87 (m). MS (ESI) *m/z* 322.1 $(M+H)^+$. HRMS (ESI) Calcd. for $C_{20}H_{17}FNO_2$ $(M+H)^+$: 322.1238, Found: 322.1241.

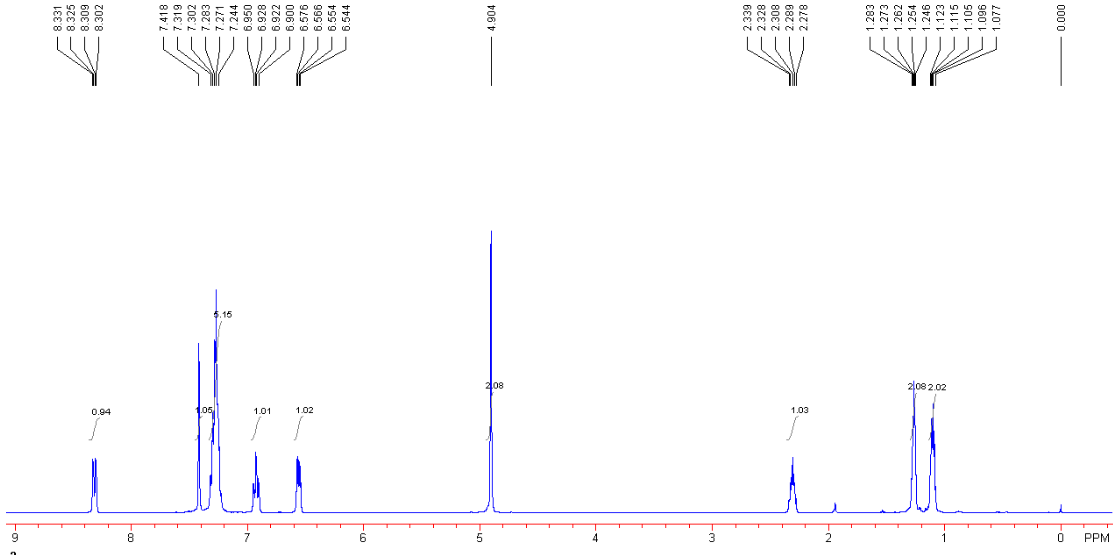

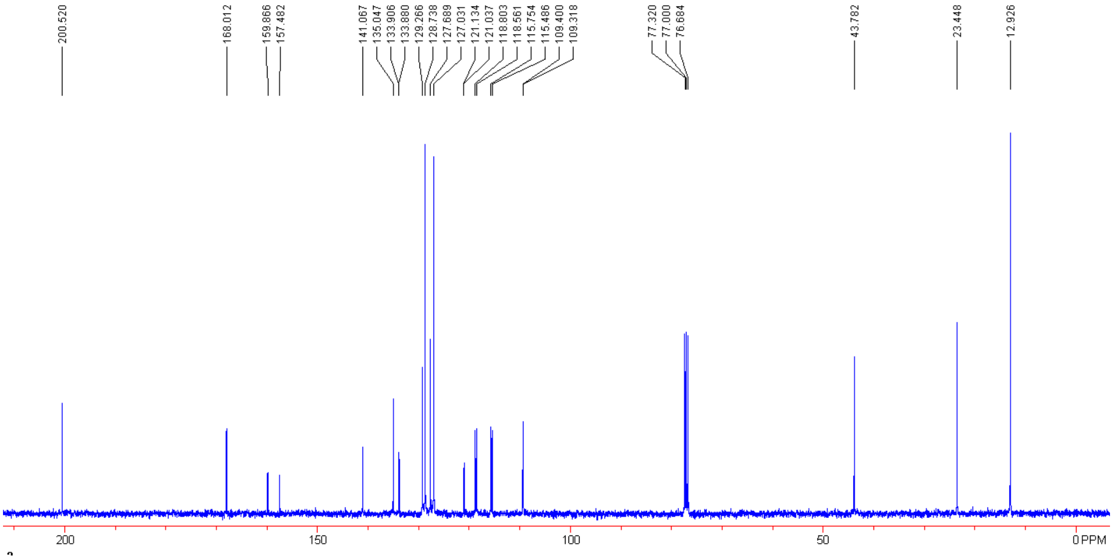
200.520
168.012
159.866
157.482
141.067
135.047
133.906
133.880
129.266
128.738
127.689
127.031
121.134
121.037
118.803
118.561
115.754
115.486
109.400
109.318
77.320
77.000
76.684
43.782
23.448
12.926
200
150
100
50
0 PPM

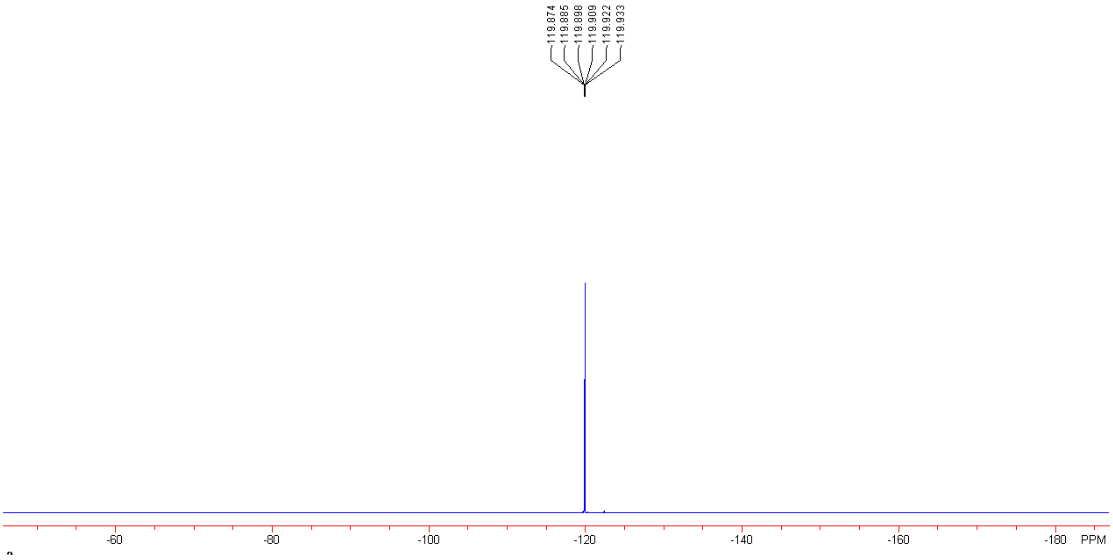
119.874
119.885
119.898
119.909
119.922
119.933
-60
-80
-100
-120
-140
-160
-180
PPM

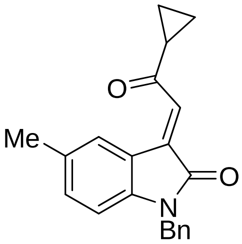

Compound 2h: Yield: 121 mg. 58%. A red solid. Mp: 111-113 °C. IR (neat) ν 2925, 1713, 1673, 1378, 1186, 749 cm^{-1}. ^{1}H NMR ($CDCl_3$, 400 MHz, TMS) δ 1.06-1.10 (m, 2H), 1.24-1.28 (m, 2H), 2.28 (s, 3H), 2.28-2.34 (m, 1H), 4.90 (s, 2H), 6.54 (d, J = 8.0 Hz, 1H), 7.03 (d, J = 8.0 Hz, 1H), 7.23-7.31 (m, 5H), 7.38 (s, 1H), 8.34 (s, 1H). ^{13}C NMR ($CDCl_3$, 100 MHz, TMS) δ 12.6, 21.0, 23.4, 43.7, 108.7, 120.2, 127.0, 127.5, 127.9, 128.61, 128.65, 132.2, 132.9, 134.6, 135.4, 142.8, 168.3, 200.8. MS (ESI) m/z 318.1 $(M+H)^+$. HRMS (ESI) Calcd. for $C_{21}H_{20}NO_2$ $(M+H)^+$: 318.1489, Found: 318.1487.

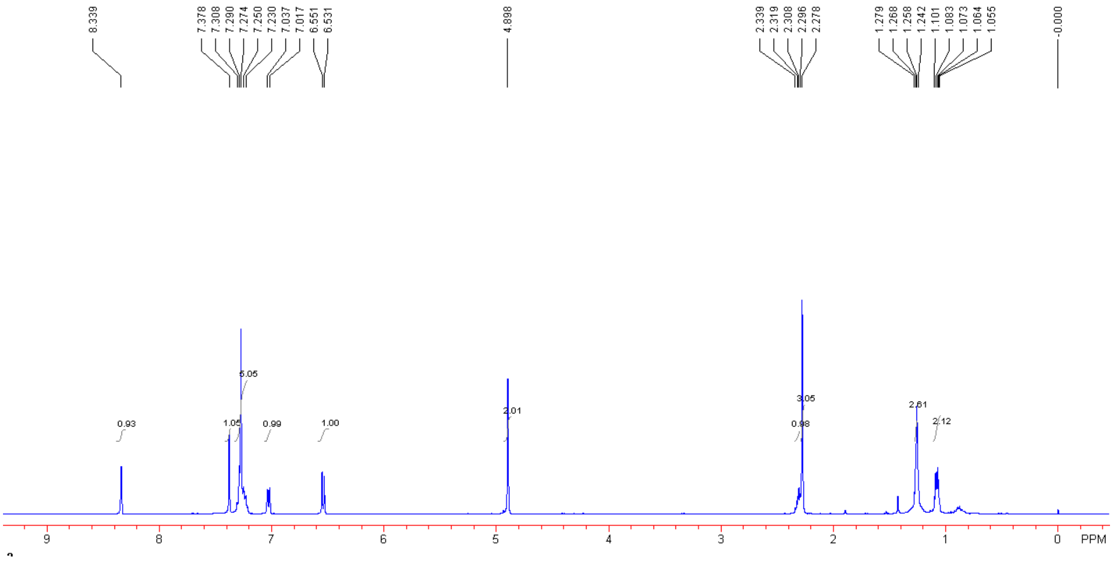

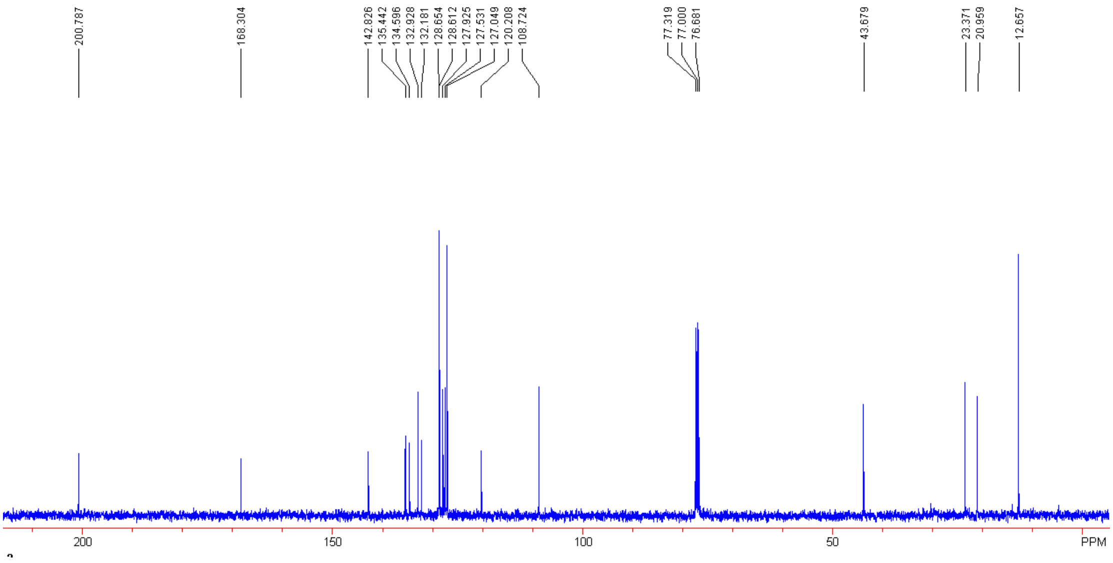

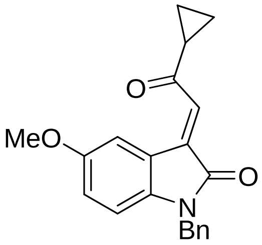

Compound 2i: Yield: 154 mg. 61%. A red solid. Mp: 140-142 °C. IR (neat) ν 3060, 2952, 1705, 1664, 1479, 1118, 822 cm^{-1}. ^{1}H NMR ($CDCl_3$, 400 MHz, TMS) δ 1.07-1.11 (m, 2H), 1.24-1.28 (m, 2H), 2.29-2.35 (m, 1H), 3.78 (s, 3H), 4.90 (s, 2H), 6.55 (d, J = 8.8 Hz, 1H), 6.80 (dd, J = 2.8 Hz, J = 8.8 Hz, 1H), 7.25-7.32 (m, 5H), 7.40 (s, 1H), 8.22 (d, J = 2.8 Hz, 1H). ^{13}C NMR ($CDCl_3$, 100 MHz, TMS) δ 12.7, 23.5, 43.8, 55.8, 109.5, 113.6, 118.6, 120.9, 127.1, 127.6, 128.4, 128.7, 134.8, 135.5, 138.9, 155.6, 168.2, 200.8. MS (ESI) m/z 334.1 $(M+H)^+$. HRMS (ESI) Calcd. for $C_{21}H_{20}NO_3$ $(M+H)^+$: 334.1438, Found: 334.1427.

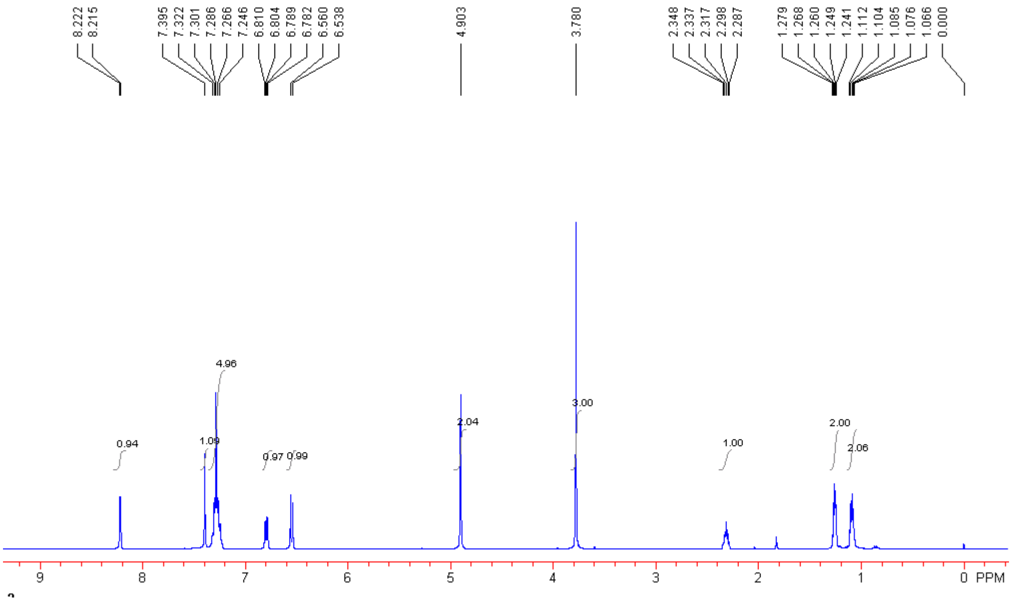

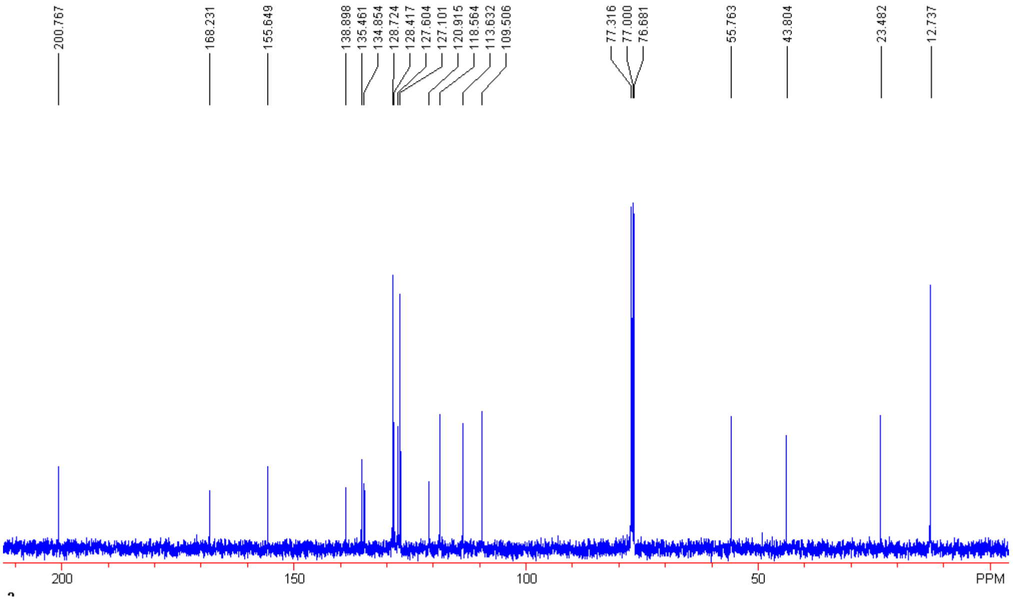
200.767
168.231
155.649
138.898
135.461
134.854
128.724
128.417
127.604
127.101
120.915
118.564
113.632
109.506
77.316
77.000
76.681
55.763
43.804
23.482
12.737
200
150
100
50
PPM

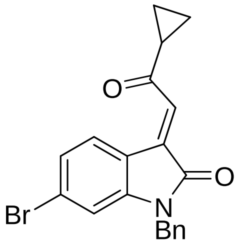

Compound 2j: Yield: 92 mg. 58%. A yellow solid. Mp: 126-128 °C. IR (neat) ν 3005, 2940, 1713, 1663, 1379, 1178, 746 cm^{-1}. ^{1}H NMR ($CDCl_3$, 400 MHz, TMS) δ 1.07-1.11 (m, 2H), 1.24-1.25 (m, 2H), 2.27-2.33 (m, 1H), 4.89 (s, 2H), 6.82 (s, 1H), 7.10 (d, *J* = 8.0 Hz, 1H), 7.26-7.34 (m, 5H), 7.40 (s, 1H), 8.40 (d, *J* = 8.4 Hz, 1H). ^{13}C NMR ($CDCl_3$, 100 MHz, TMS) δ 12.9, 23.5, 43.8, 112.3, 119.1, 125.7, 126.7, 127.0, 127.8, 128.6, 128.8, 129.3, 133.3, 134.8, 146.1, 168.2, 200.6. MS (ESI) *m/z* 382.0 $(M+H)^+$. HRMS (ESI) Calcd. for $C_{20}H_{17}NO_2Br$ $(M+H)^+$: 382.0437, Found: 382.0446.

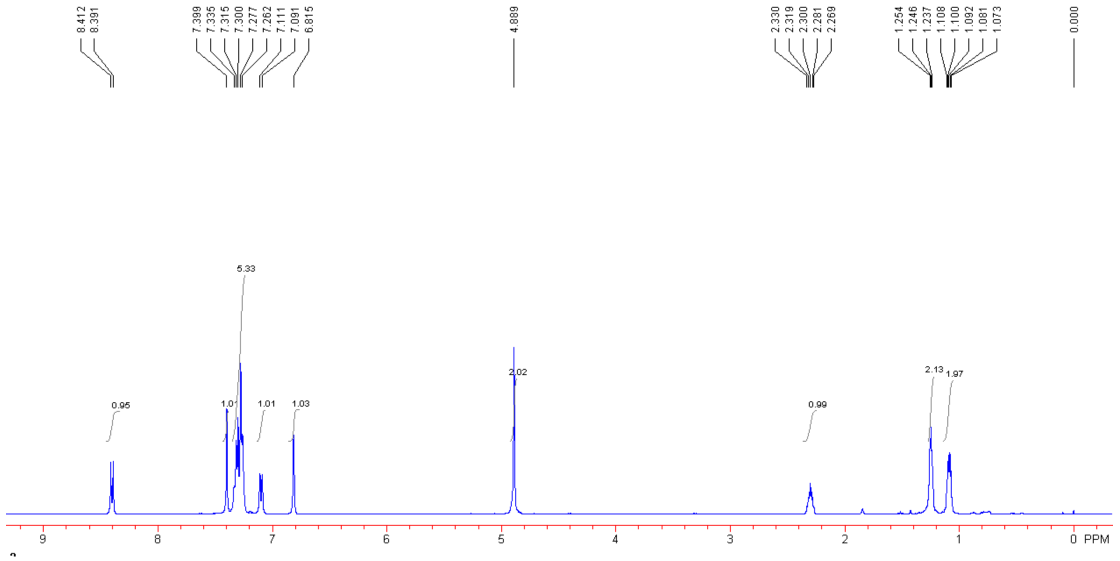

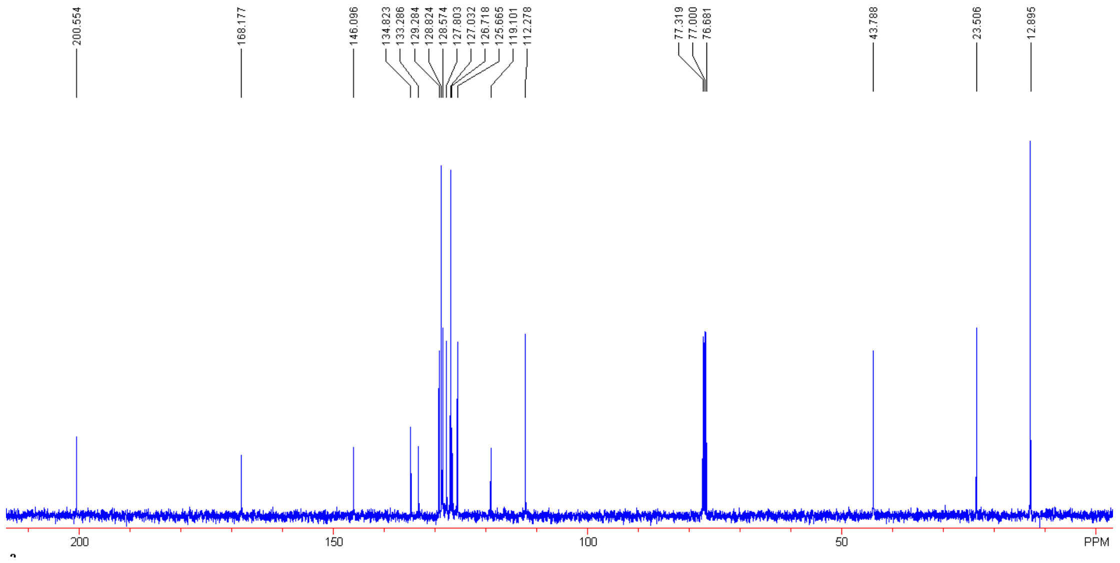

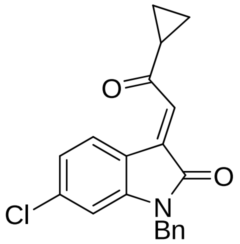

Compound 2k: Yield: 69 mg. 53%. A yellow solid. Mp: 135-137 °C. IR (neat) ν 2990, 1711, 1663, 1379, 1178, 742 cm^{-1}. ^{1}H NMR (CDCl$_3$, 400 MHz, TMS) δ 1.07-1.11 (m, 2H), 1.23-1.27 (m, 2H), 2.27-2.34 (m, 1H), 4.90 (s, 2H), 6.66 (d, *J* = 2.0 Hz, 1H), 6.94 (dd, *J* = 2.0 Hz, *J* = 8.4 Hz, 1H), 7.26-7.34 (m, 5H), 7.38 (s, 1H), 8.48 (d, *J* = 8.4 Hz, 1H). ^{13}C NMR (CDCl$_3$, 100 MHz, TMS) δ 12.9, 23.5, 43.8, 109.5, 118.7, 122.7, 127.1, 127.8, 128.4, 128.8, 129.2, 133.2, 134.8, 138.3, 146.2, 168.4, 200.6. MS (ESI) *m/z* 338.0 (M+H)$^+$. HRMS (ESI) Calcd. for $C_{20}H_{17}NO_2Cl$ (M+H)$^+$: 338.0942, Found: 338.0930.

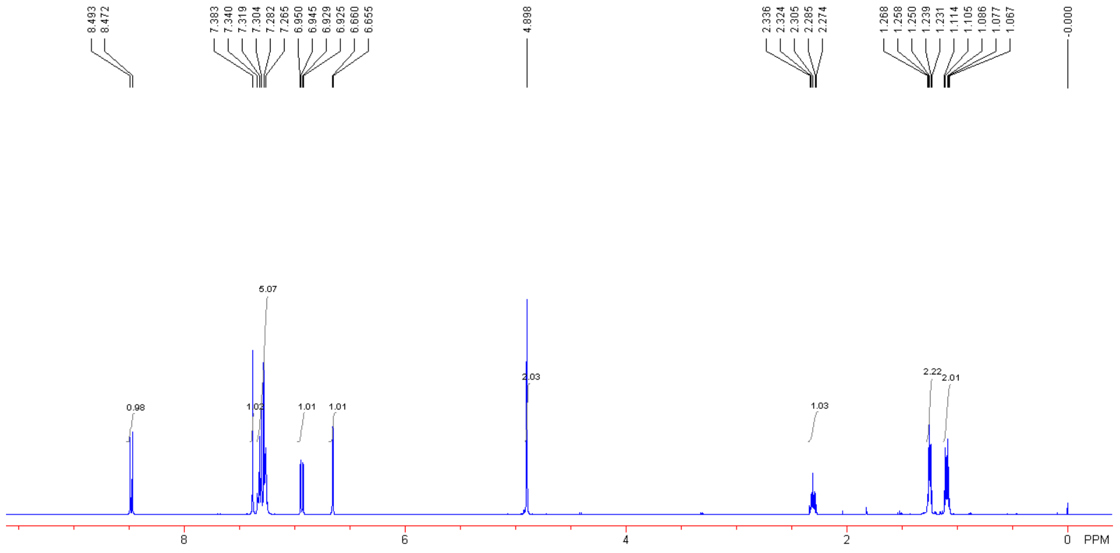

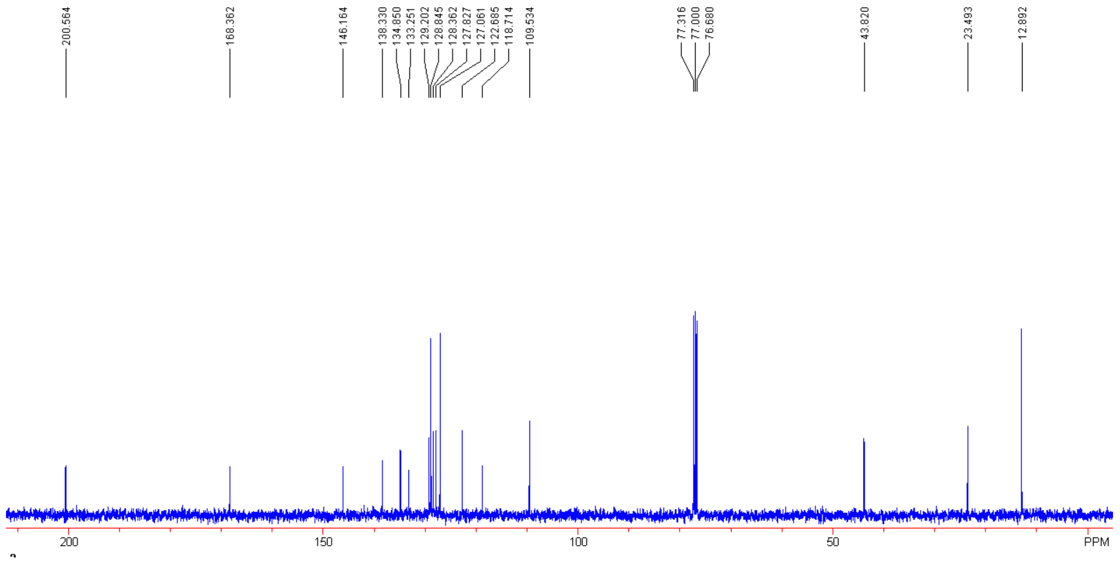

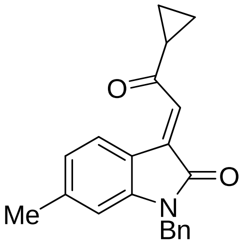

Compound 2l: Yield: 126 mg. 59%. A red solid. Mp: 151-153 °C. IR (neat) ν 3029, 2918, 1713, 1665, 1378, 1115, 693 cm^{-1}. ^{1}H NMR ($CDCl_3$, 400 MHz, TMS) δ 1.02-1.06 (m, 2H), 1.21-1.25 (m, 2H), 2.26 (s, 3H), 2.26-2.31 (m, 1H), 4.89 (s, 2H), 6.48 (s, 1H), 6.77 (d, J = 7.6 Hz, 1H), 7.23-7.29 (m, 5H), 7.32 (s, 1H), 8.42 (d, J = 7.6 Hz, 1H). ^{13}C NMR ($CDCl_3$, 100 MHz, TMS) δ 12.5, 22.2, 23.3, 43.5, 109.7, 117.8, 123.3, 126.7, 126.9, 127.5, 128.0, 128.6, 134.3, 135.5, 143.7, 145.4, 168.7, 200.6. MS (ESI) m/z 340.1 $(M+Na)^{+}$. HRMS (ESI) Calcd. for $C_{21}H_{19}NNaO_2$ $(M+Na)^{+}$: 340.1308, Found: 340.1308.

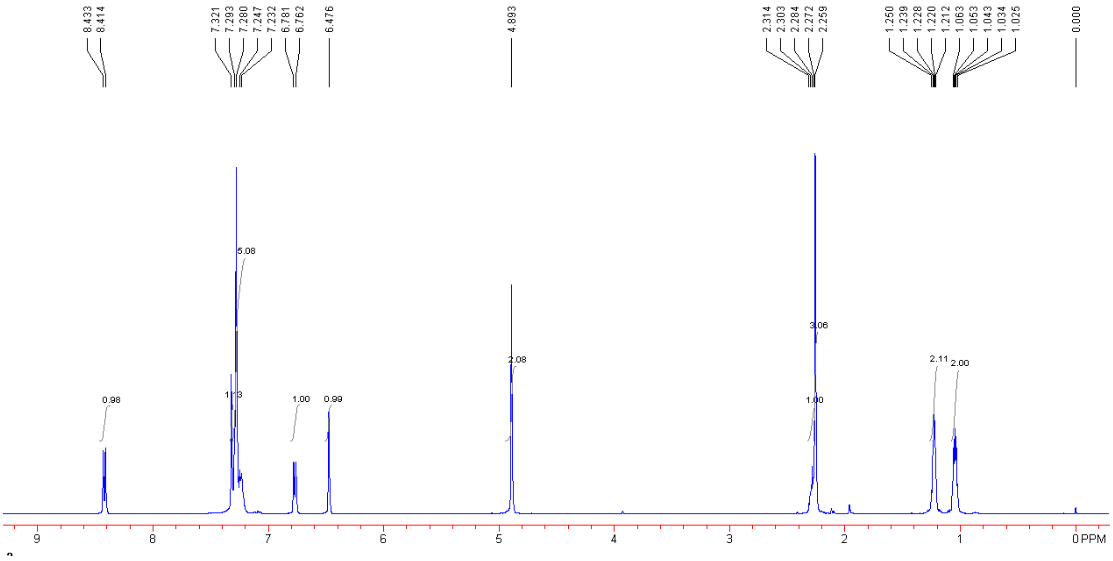

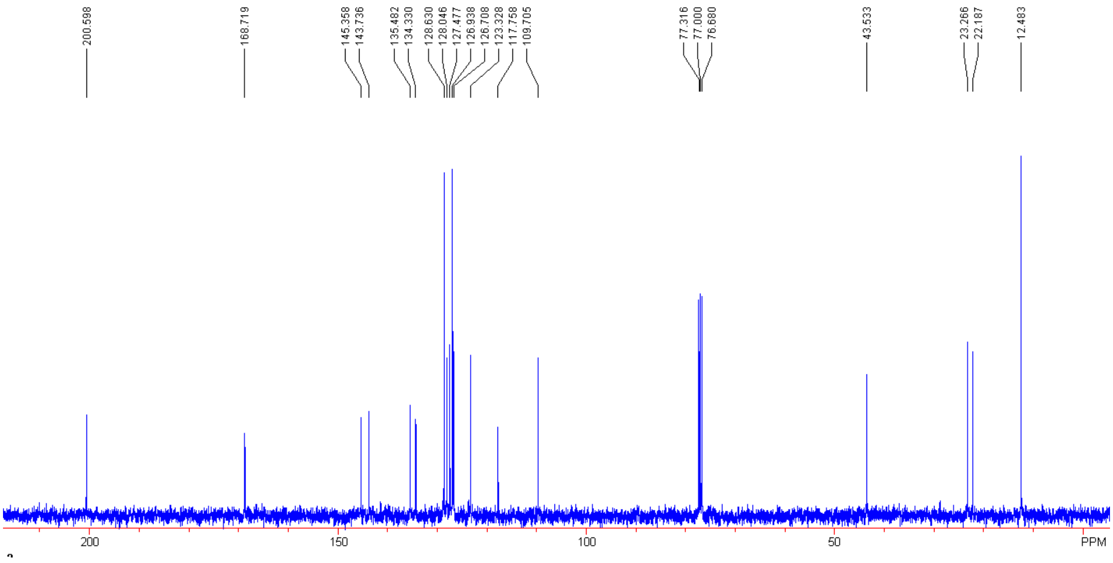

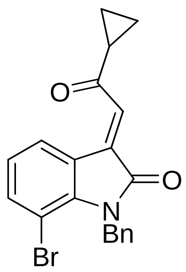

Compound 2m: Yield: 107 mg. 47%. A yellow solid. Mp: 181-183 °C. IR (neat) ν 2996, 1708, 1666, 1346, 1177, 741 cm^{-1}. ^{1}H NMR ($CDCl_3$, 400 MHz, TMS) δ 1.07-1.12 (m, 2H), 1.24-1.28 (m, 2H), 2.28-2.34 (m, 1H), 5.45 (s, 2H), 6.86 (t, *J* = 8.0 Hz, 1H), 7.19-7.31 (m, 5H), 7.39 (d, *J* = 8.0 Hz, 1H), 7.43 (s, 1H), 8.55 (d, *J* = 7.6 Hz, 1H). ^{13}C NMR ($CDCl_3$, 100 MHz, TMS) δ 13.0, 23.6, 44.8, 102.4, 123.4, 124.0, 126.2, 127.0, 127.1, 128.5, 129.6, 132.6, 137.0, 138.2, 142.2, 169.2, 200.8. MS (ESI) *m/z* 382.0 (M+H)$^+$. HRMS (ESI) Calcd. for $C_{20}H_{17}NO_2Br$ (M+H)$^+$: 382.0437, Found: 382.0421.

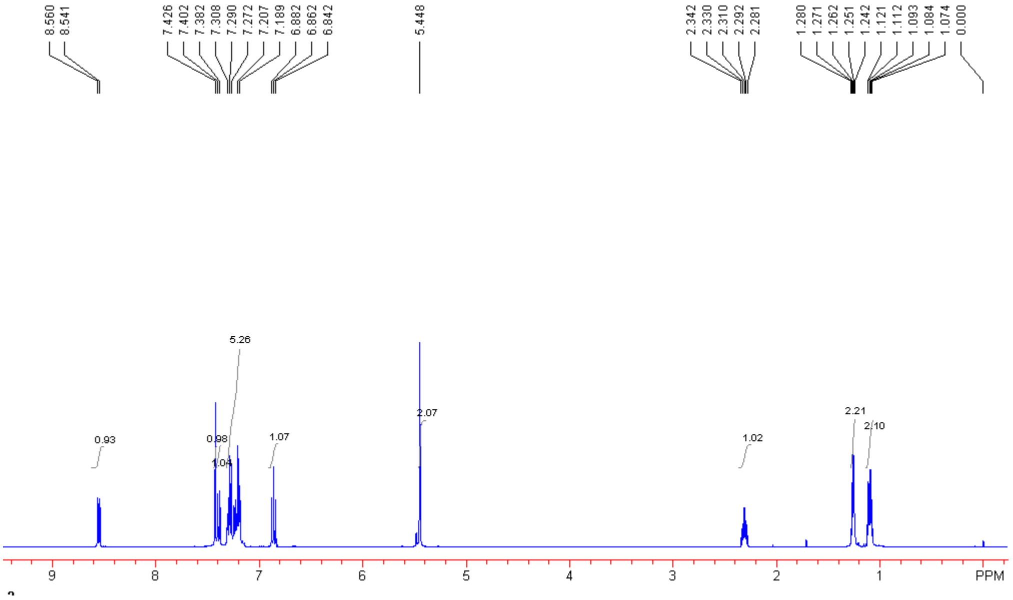

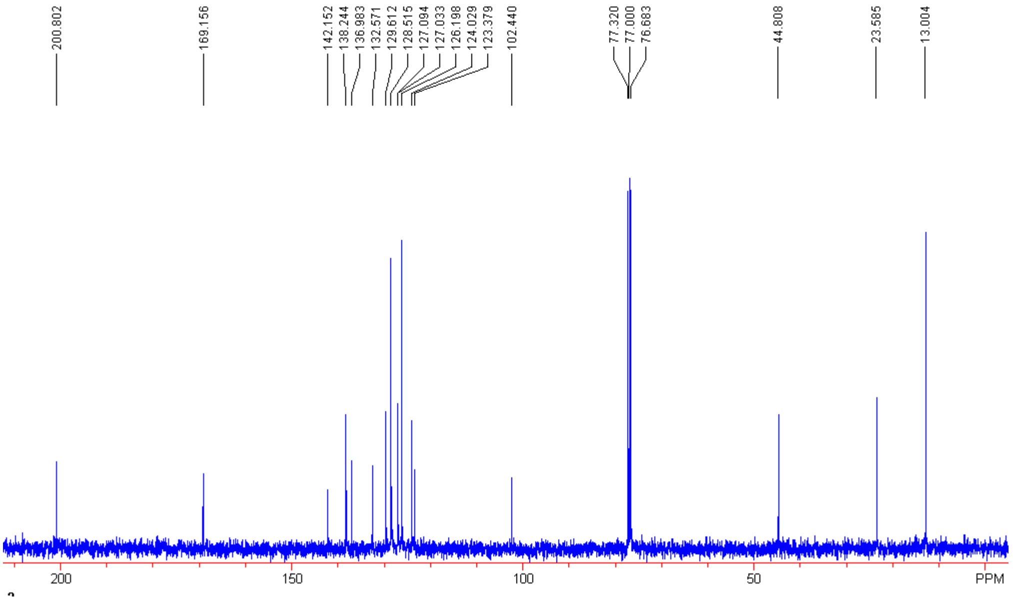

200.802
169.156
142.152
138.244
136.983
132.571
129.612
128.515
127.094
127.033
126.198
124.029
123.379
102.440
77.320
77.000
76.683
44.808
23.585
13.004
200
150
100
50
PPM

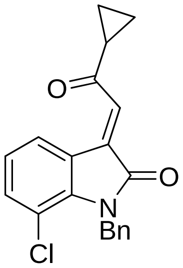

Compound 2n: Yield: 80 mg. 44%. A yellow solid. Mp: 186-188 °C. IR (neat) ν 3100, 2928, 1709, 1667, 1348, 1135, 741 cm^{-1}. ^{1}H NMR ($CDCl_3$, 400 MHz, TMS) δ 1.09-1.13 (m, 2H), 1.26-1.29 (m, 2H), 2.29-2.35 (m, 1H), 5.41 (s, 2H), 6.94 (t, *J* = 8.0 Hz, 1H), 7.21-7.32 (m, 6H), 7.44 (s, 1H), 8.49 (d, *J* = 7.6 Hz, 1H). ^{13}C NMR ($CDCl_3$, 100 MHz, TMS) δ 13.0, 23.6, 45.2, 115.5, 123.1, 123.7, 126.4, 126.5, 127.2, 128.6, 129.8, 132.7, 134.9, 137.1, 140.8, 169.0, 200.9. MS (ESI) *m/z* 338.1 $(M+H)^+$. HRMS (ESI) Calcd. for $C_{20}H_{17}NO_2Cl$ $(M+H)^+$: 338.0942, Found: 338.0951.

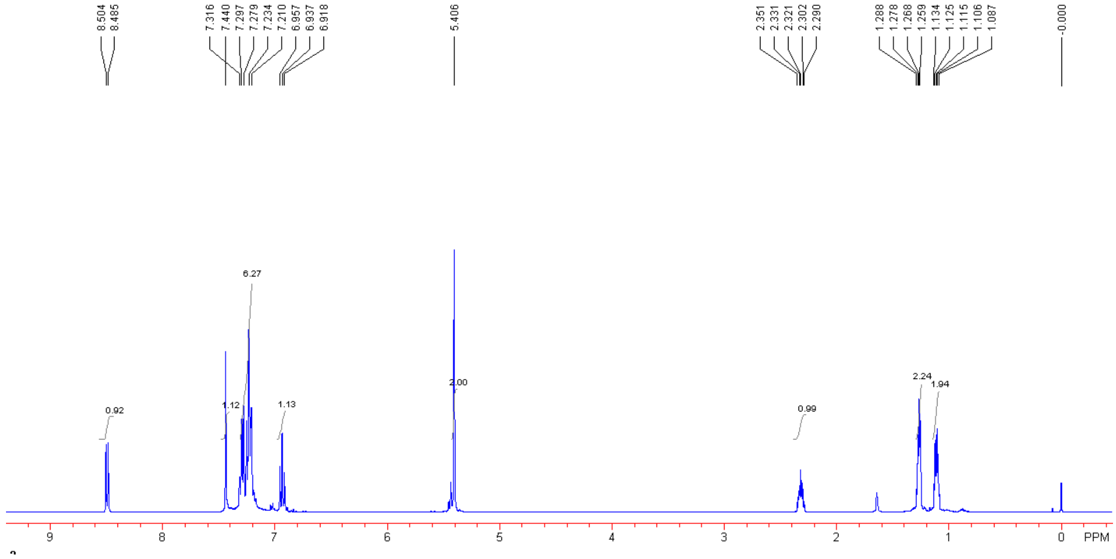

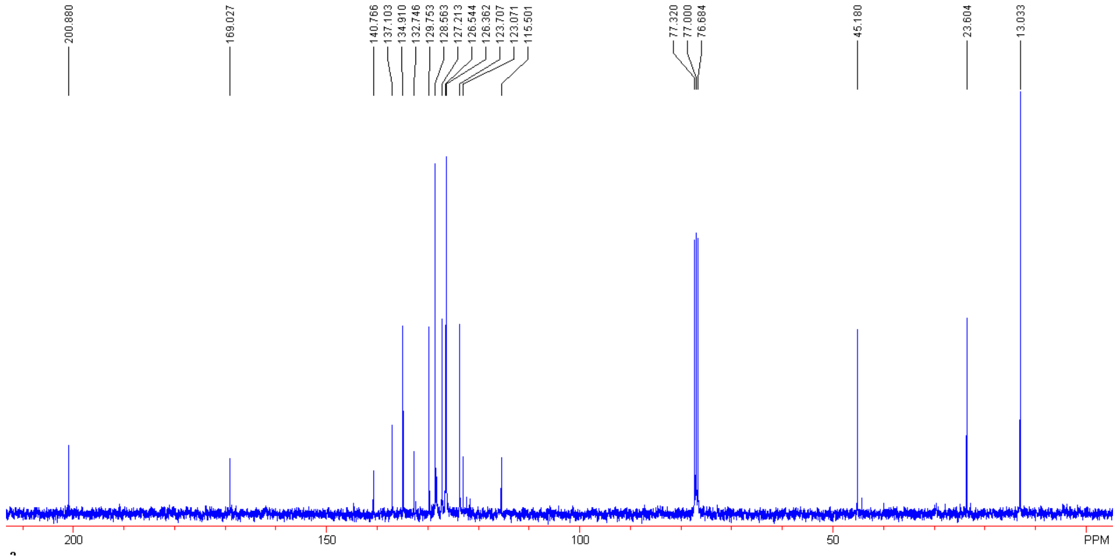
200.880
169.027
140.766
137.103
134.910
132.746
129.753
128.563
127.213
126.544
126.362
123.707
123.071
115.501
77.320
77.000
76.684
45.180
23.604
13.033
200
150
100
50
PPM

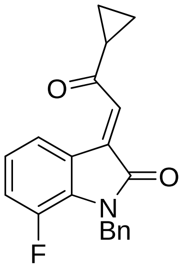

Compound 2o: Yield: 34 mg. 45%. A red solid. Mp: 128-130 °C. IR (neat) ν 2926, 1718, 1676, 1376, 1181, 796 cm^{-1}. ^{1}H NMR ($CDCl_3$, 400 MHz, TMS) δ 1.07-1.12 (m, 2H), 1.24-1.28 (m, 2H), 2.28-2.34 (m, 1H), 5.09 (s, 2H), 6.90-6.95 (m, 1H), 7.03 (dd, J = 8.0 Hz, J = 11.2 Hz, 1H), 7.25-7.34 (m, 5H), 7.42 (s, 1H), 8.30 (d, J = 7.6 Hz, 1H). ^{13}C NMR ($CDCl_3$, 100 MHz, TMS) δ 12.9, 23.5, 45.5 (d, J = 5.2 Hz), 120.6 (d, J = 19.3 Hz), 123.0 (d, J = 3.7 Hz), 123.3 (d, J = 6.3 Hz), 124.0 (d, J = 2.9 Hz), 127.4, 127.6, 128.6, 129.7, 131.4 (d, J = 8.5 Hz), 133.4 (d, J = 3.4 Hz), 136.6, 147.2 (d, J = 242.4 Hz), 168.1, 200.7. ^{19}F NMR ($CDCl_3$, 376 MHz, CF_3COOH) δ -134.02 ~ -133.98 (m). MS (ESI) m/z 322.1 $(M+H)^+$. HRMS (ESI) Calcd. for $C_{20}H_{17}NO_2F$ $(M+H)^+$: 322.1238, Found: 322.1230.

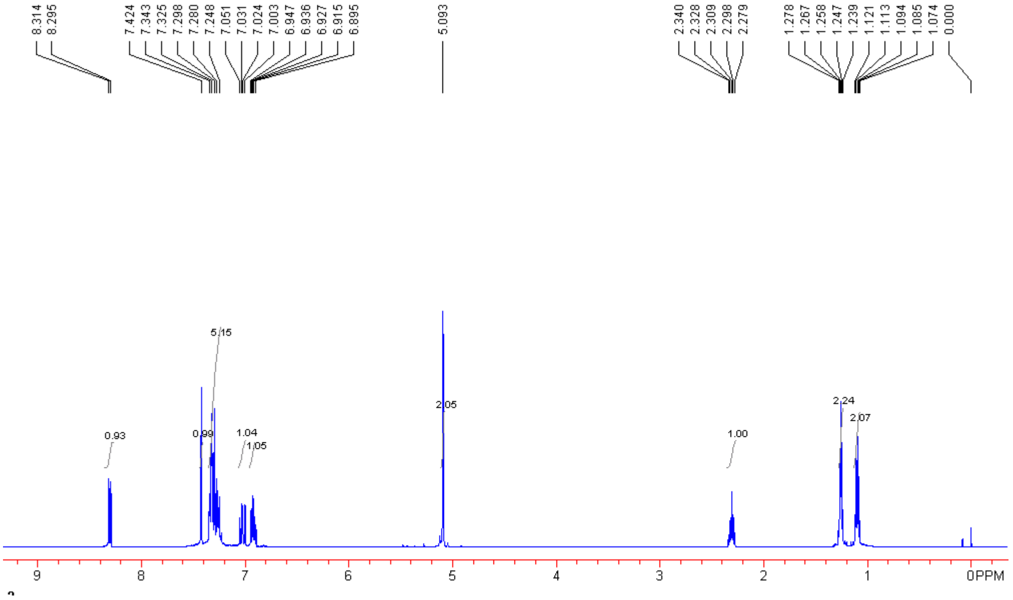

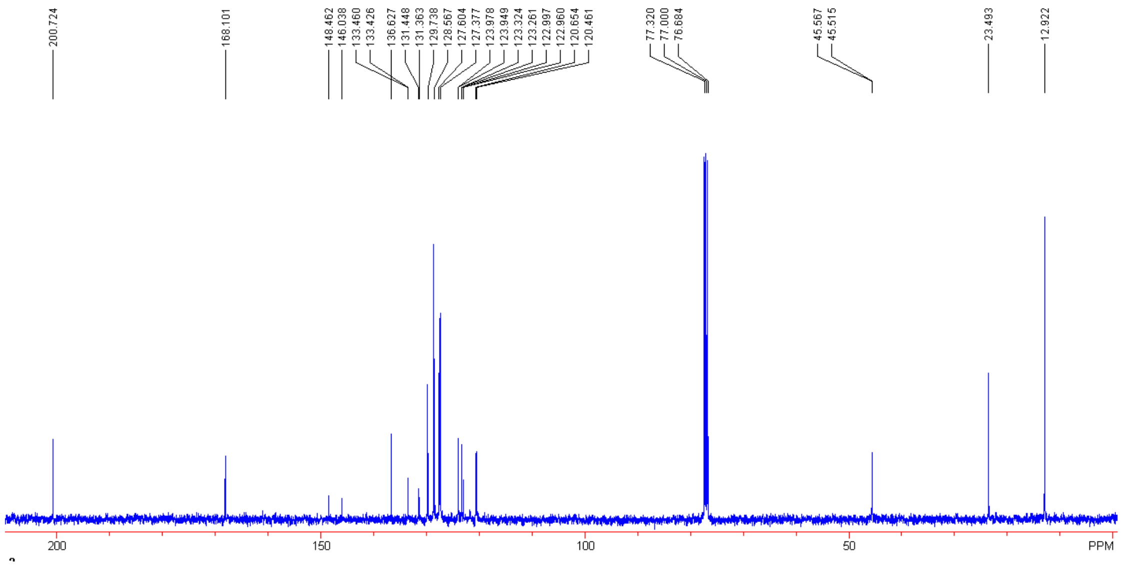
200.724
168.101
148.462
146.038
133.460
133.426
136.627
131.448
131.363
129.738
128.567
127.604
127.377
123.978
123.949
123.324
123.261
122.997
122.960
120.654
120.461
77.320
77.000
76.684
45.567
45.515
23.493
12.922
200
150
100
50
PPM

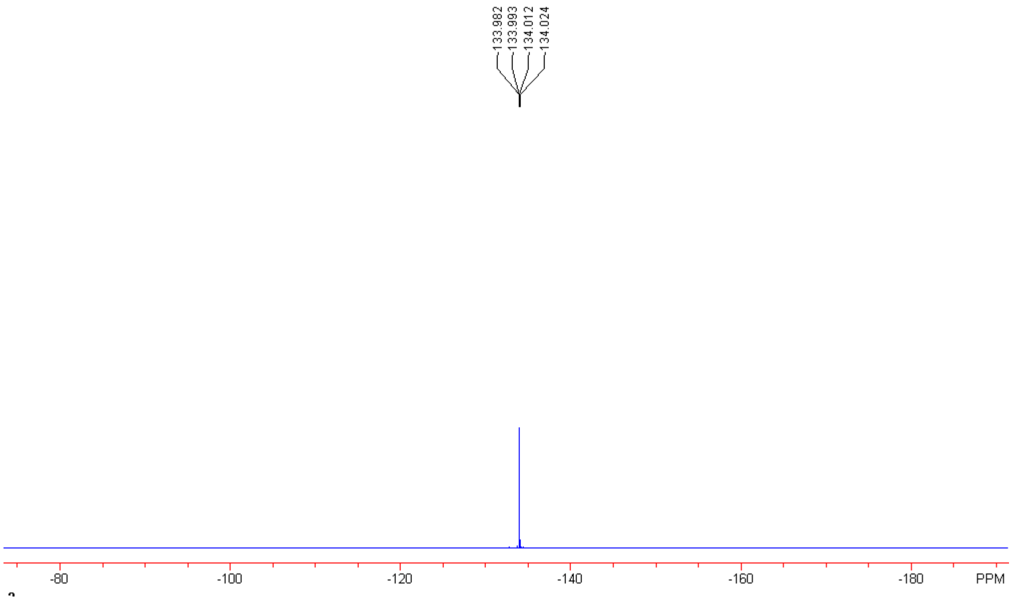
133.982
133.993
134.012
134.024
-80
-100
-120
-140
-160
-180
PPM

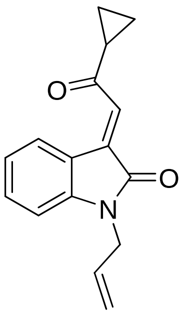

Compound 2p: Yield: 73 mg. 52%. A red solid. Mp: 95-97 ^{o}C. IR (neat) ν 2923, 1714, 1663, 1353, 1189, 754 cm^{-1}. ^{1}H NMR ($CDCl_3$, 400 MHz, TMS) δ 1.06-1.11 (m, 2H), 1.23-1.27 (m, 2H), 2.28-2.34 (m, 1H), 4.36 (dt, *J* = 5.2 Hz, *J* = 1.6 Hz, 2H), 5.21-5.25 (m, 2H), 5.79-5.88 (m, 1H), 6.77 (d, *J* = 8.0 Hz, 1H), 7.01 (dt, *J* = 0.8 Hz, *J* = 7.6 Hz, 1H), 7.28-7.34 (m, 2H), 8.50 (d, *J* = 7.6 Hz, 1H). ^{13}C NMR ($CDCl_3$, 100 MHz, TMS) δ 12.6, 23.3, 42.3, 108.8, 117.5, 120.1, 122.6, 128.0, 128.1, 131.0, 132.5, 134.2, 145.1, 167.9, 200.7. MS (ESI) *m/z* 254.1 (M+H)$^{+}$. HRMS (ESI) Calcd. for $C_{16}H_{16}NO_2$ (M+H)$^{+}$: 254.1176, Found: 254.1178.

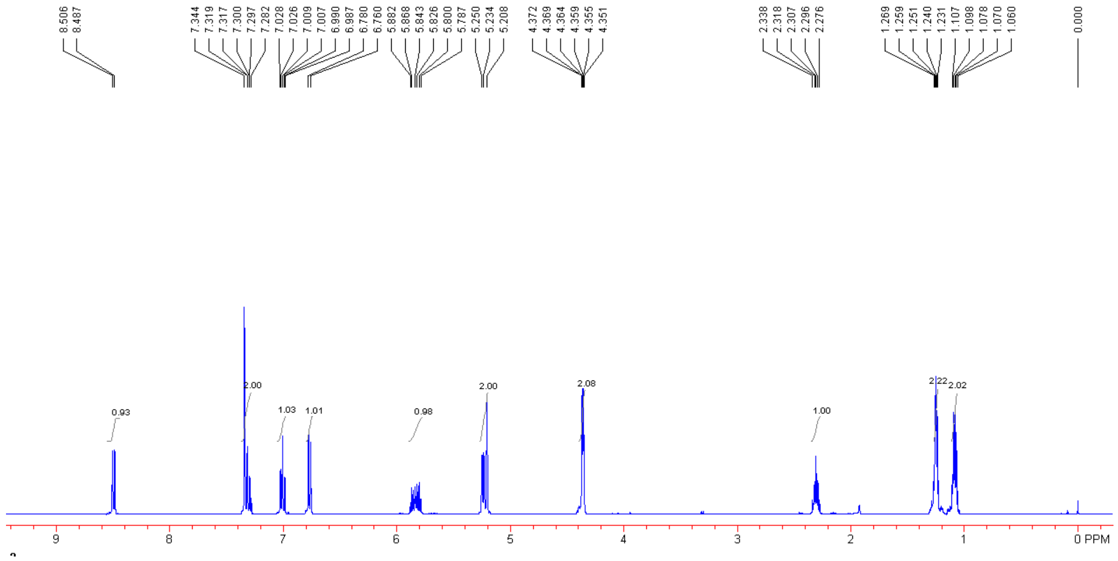

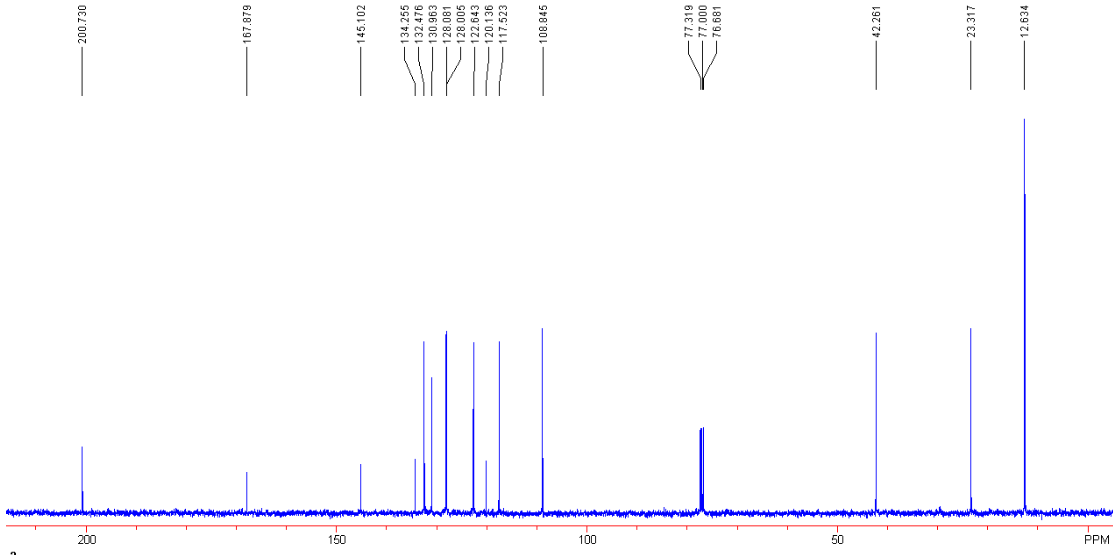
200.730
167.879
145.102
134.255
132.476
130.963
128.081
128.005
122.643
120.136
117.523
108.845
77.319
77.000
76.681
42.261
23.317
12.634
200
150
100
50
PPM

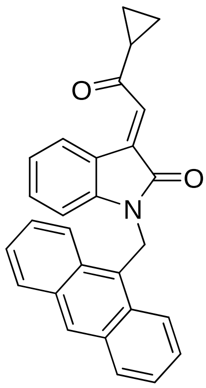

Compound 2q: Yield: 35 mg. 34%. A red solid. Mp: 210-212 °C. IR (neat) ν 2927, 1709, 1673, 1381, 1178, 731 cm^{-1}. ^{1}H NMR ($CDCl_3$, 400 MHz, TMS) δ 1.07-1.11 (m, 2H), 1.23-1.27 (m, 2H), 2.31-2.37 (m, 1H), 5.95 (s, 2H), 6.25 (d, *J* = 7.6 Hz, 1H), 6.75-6.84 (m, 2H), 7.47-7.50 (m, 3H), 7.56-7.60 (m, 2H), 8.03 (d, *J* = 8.4 Hz, 2H), 8.37 (d, *J* = 8.0 Hz, 1H), 8.43 (d, *J* = 9.2 Hz, 2H), 8.47 (s, 1H). ^{13}C NMR ($CDCl_3$, 100 MHz, TMS) δ 12.8, 23.5, 37.8, 110.1, 120.4, 122.5, 123.6, 125.1, 125.5, 127.0, 127.8, 128.5, 129.0, 129.6, 130.8, 131.3, 132.5, 134.2, 145.3, 168.5, 200.9. MS (ESI) *m/z* 404.2 (M+H)$^{+}$. HRMS (ESI) Calcd. for $C_{28}H_{22}NO_2$ (M+H)$^{+}$: 404.1645, Found: 404.1650.

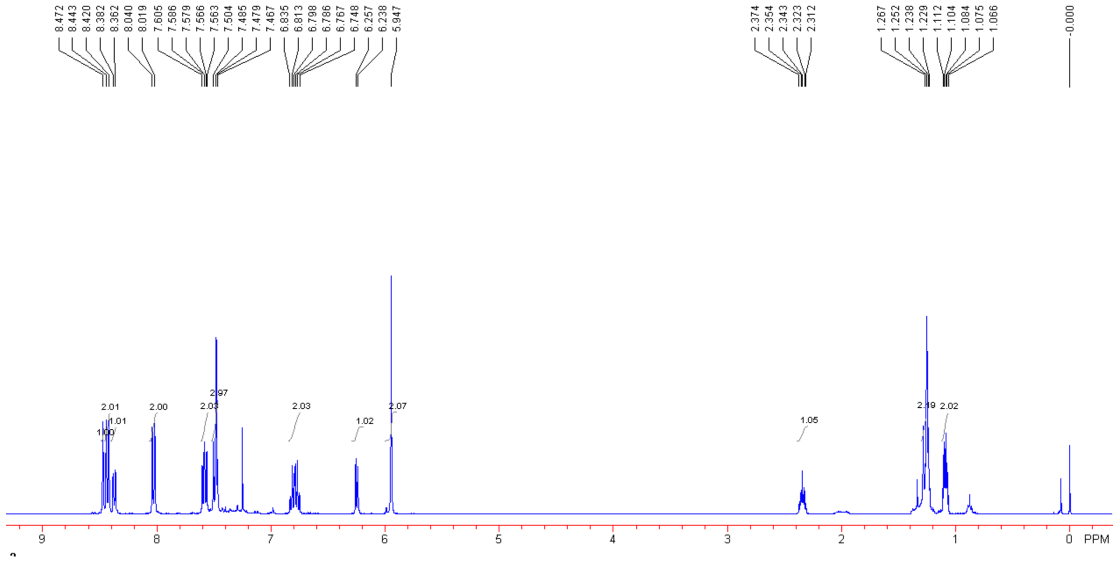

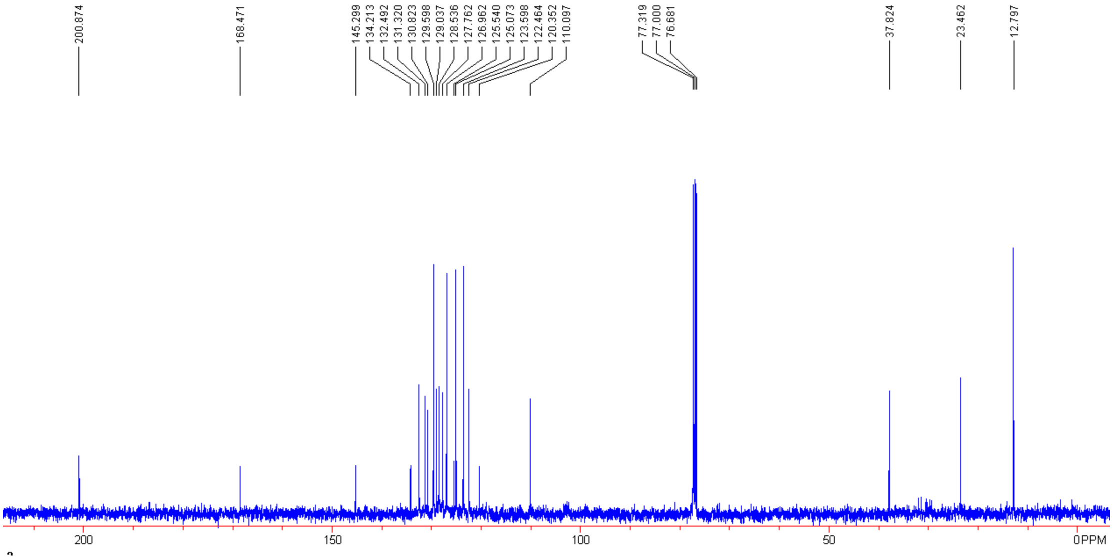
200.874
168.471
145.299
134.213
132.492
131.320
130.823
129.598
129.037
128.536
127.762
126.962
125.540
125.073
123.598
122.464
120.352
110.097
77.319
77.000
76.681
37.824
23.462
12.797
200
150
100
50
0PPM

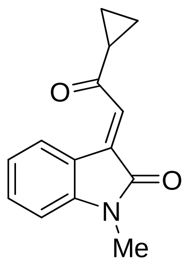

Compound 2r: Yield: 93 mg. 55%. A red solid. Mp: 93-95 °C. IR (neat) ν 2923, 1712, 1666, 1378, 1133, 745 cm^{-1}. ^{1}H NMR ($CDCl_3$, 400 MHz, TMS) δ 1.04-1.09 (m, 2H), 1.21-1.25 (m, 2H), 2.24-2.30 (m, 1H), 3.17 (s, 3H), 6.73 (d, J = 7.6 Hz, 1H), 6.99 (dt, J = 1.2 Hz, J = 8.0 Hz, 1H), 7.31-7.35 (m, 2H), 8.45 (d, J = 8.0 Hz, 1H). ^{13}C NMR ($CDCl_3$, 100 MHz, TMS) δ 12.6, 23.3, 26.0, 107.9, 120.0, 122.6, 126.7, 128.2, 132.5, 134.3, 145.8, 168.1, 200.8. MS (ESI) *m/z* 228.1 $(M+H)^+$. HRMS (ESI) Calcd. for $C_{14}H_{14}NO_2$ $(M+H)^+$: 228.1019, Found: 228.1019.

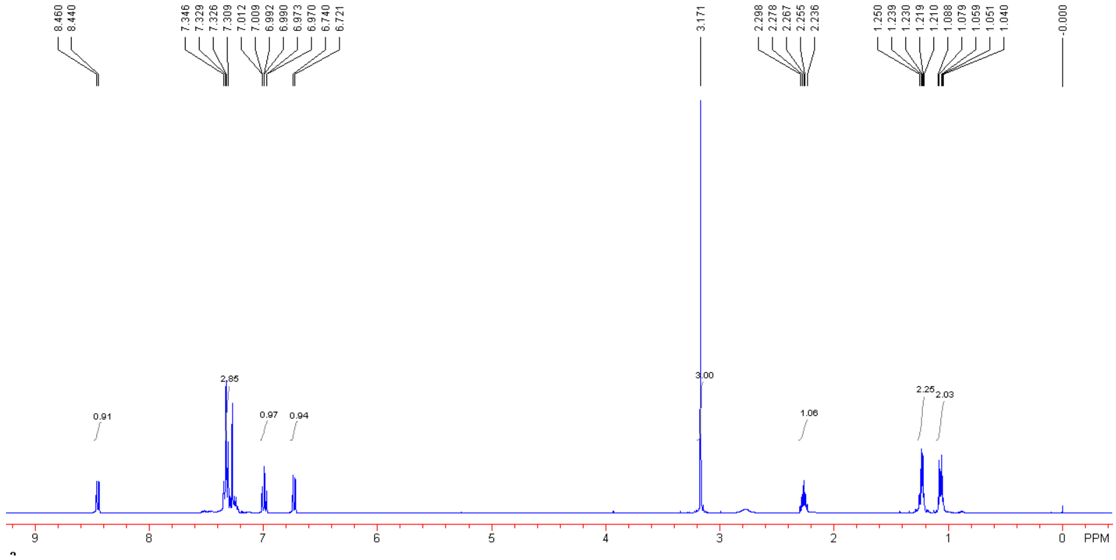

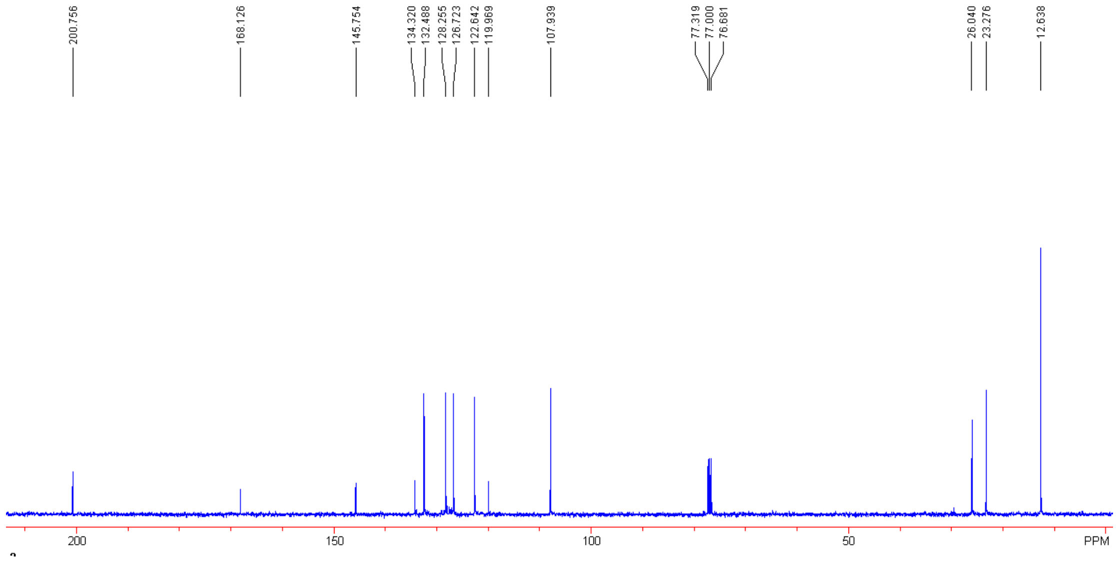

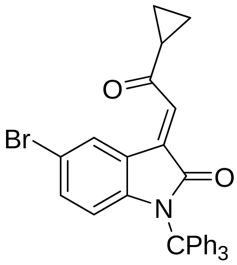

Compound 2s: Yield: 33 mg. 39%. A red solid. Mp: 203-205 °C. IR (neat) ν 3055, 2926, 1717, 1666, 1283, 706 cm^{-1}. ^{1}H NMR ($CDCl_3$, 400 MHz, TMS) δ 1.04-1.08 (m, 2H), 1.22-1.25 (m, 2H), 2.19-2.25 (m, 1H), 6.12 (d, J = 8.8 Hz, 1H), 7.06 (dd, J = 2.4 Hz, J = 8.8 Hz, 1H), 7.22-7.28 (m, 10H), 7.40 (d, J = 7.6 Hz, 6H), 8.68 (d, J = 2.0 Hz, 1H). ^{13}C NMR ($CDCl_3$, 100 MHz, TMS) δ 12.8, 23.6, 74.9, 115.4, 117.1, 122.7, 127.1, 127.7, 128.8, 129.3, 129.9, 133.6, 133.9, 141.4, 144.7, 168.6, 200.8. MS (ESI) *m/z* 556.1 (M+Na)$^+$. HRMS (ESI) Calcd. for $C_{32}H_{24}NO_2BrNa$ (M+Na)$^+$: 556.0883, Found: 556.0882.

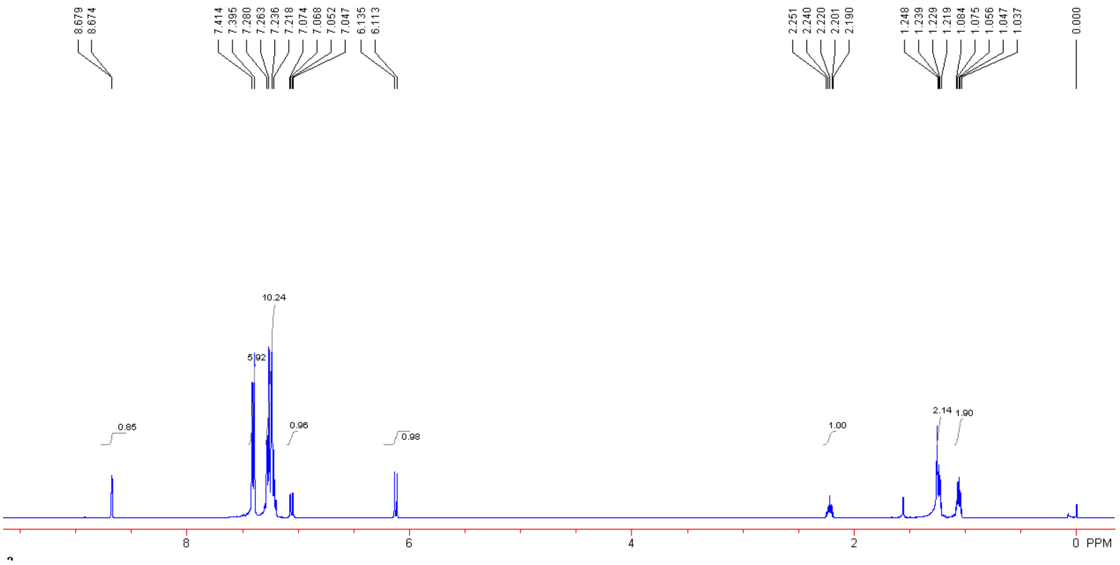

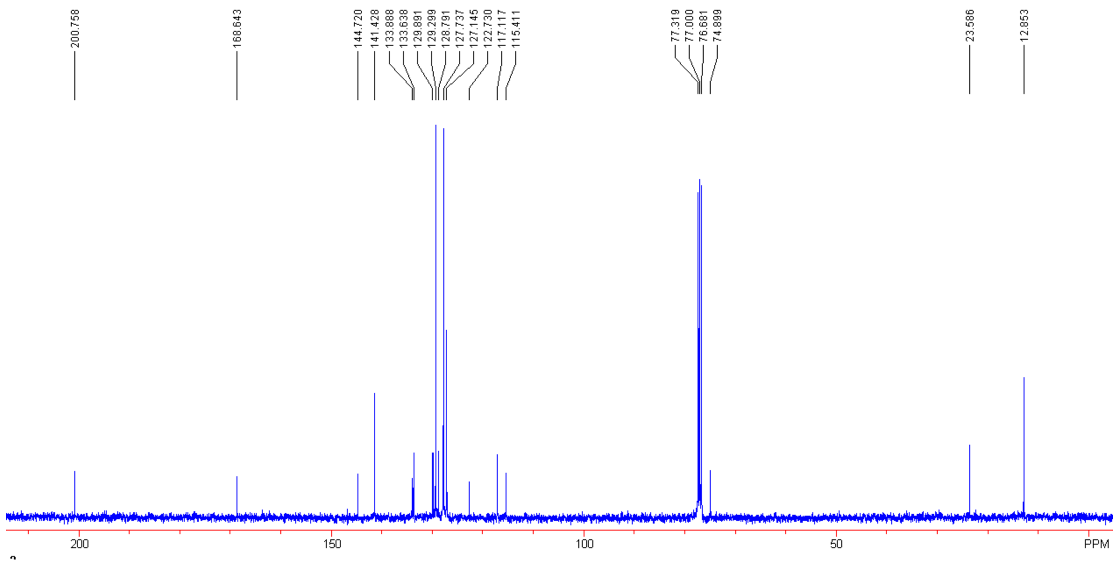
200.758
168.643
144.720
141.428
133.888
133.638
129.891
129.299
128.791
127.737
127.145
122.730
117.117
115.411
77.319
77.000
76.681
74.899
23.586
12.853
200
150
100
50
PPM

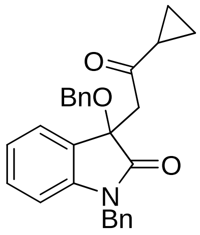

Compound 3a: Yield: 7 mg. 27%. A colorless oil. IR (neat) ν 3031, 2904, 1722, 1613, 1361, 1062, 696 cm^{-1}. ^{1}H NMR ($CDCl_3$, 400 MHz, TMS) δ 0.74-0.83 (m, 3H), 0.91-0.95 (m, 1H), 1.90-1.96 (m, 1H), 3.46 (d, J = 16.4 Hz, 1H), 3.54 (d, J = 16.4 Hz, 1H), 4.02 (d, J = 10.4 Hz, 1H), 4.24 (d, J = 10.4 Hz, 1H), 4.86 (d, J = 15.6 Hz, 1H), 5.07 (d, J = 15.6 Hz, 1H), 6.73 (d, J = 7.6 Hz, 1H), 7.05 (t, J = 7.6 Hz, 1H), 7.20-7.39 (m, 12H). ^{13}C NMR ($CDCl_3$, 100 MHz, TMS) δ 11.0, 11.1, 21.2, 44.2, 50.6, 66.5, 79.1, 109.5, 122.9, 124.1, 126.4, 127.4, 127.6, 127.8, 128.0, 128.2, 128.8, 130.1, 135.7, 137.3, 144.0, 175.0, 206.1. MS (ESI) m/z 434.1 $(M+Na)^+$. HRMS (ESI) Calcd. for $C_{27}H_{25}NO_3Na$ $(M+Na)^+$: 434.1727, Found: 434.1714.

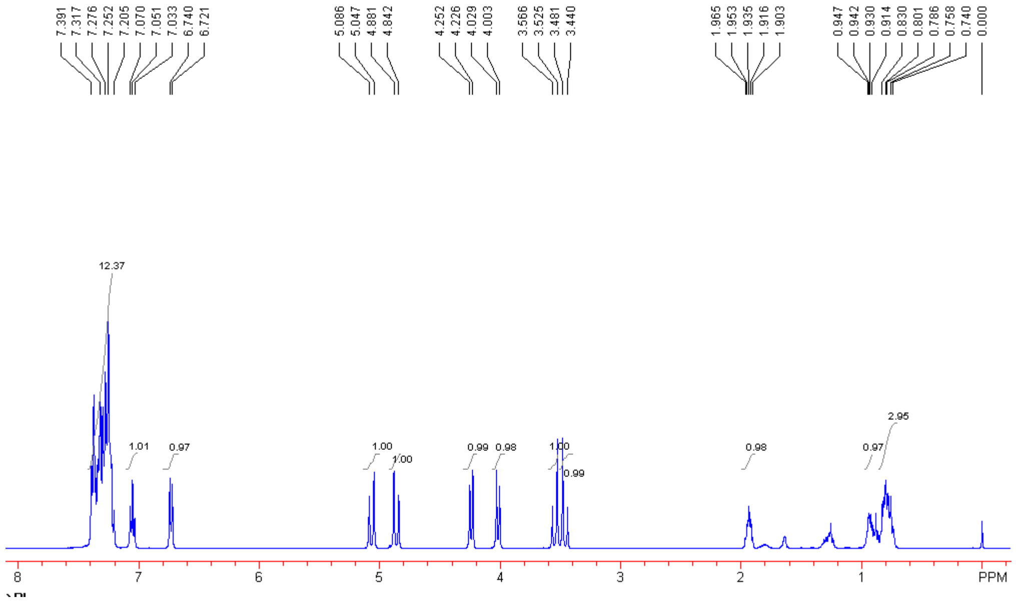

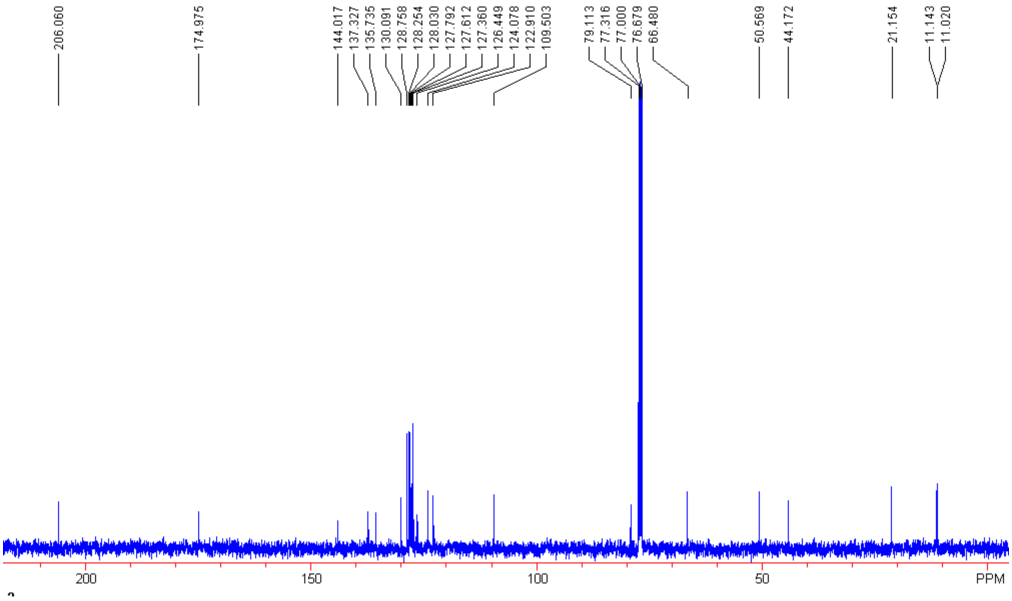
206.060
174.975
144.017
137.327
135.735
130.091
128.758
128.254
128.030
127.792
127.612
127.360
126.449
124.078
122.910
109.503
79.113
77.316
77.000
76.679
66.480
50.569
44.172
21.154
11.143
11.020
200
150
100
50
PPM

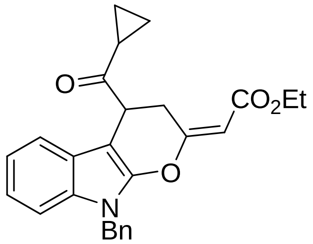

Compound 5a: Yield: 29 mg. 86%. A red oil. IR (neat) ν 3059, 2981, 1698, 1657, 1461, 1108, 735 cm^{-1}. ^{1}H NMR ($CDCl_3$, 400 MHz, TMS) δ 0.80-0.89 (m, 2H), 0.96-1.01 (m, 1H), 1.05-1.10 (m, 1H), 1.29 (t, *J* = 7.2 Hz, 3H), 2.13-2.19 (m, 1H), 3.07 (ddd, *J* = 1.6 Hz, *J* = 6.4 Hz, *J* = 15.6 Hz, 1H), 4.14-4.22 (m, 3H), 4.27 (dd, *J* = 4.0 Hz, *J* = 15.6 Hz, 1H), 5.25 (s, 2H), 5.80 (d, *J* = 1.2 Hz, 1H), 7.09-7.21 (m, 5H), 7.24-7.30 (m, 3H), 7.46 (d, *J* = 7.2 Hz, 1H). ^{13}C NMR ($CDCl_3$, 100 MHz, TMS) δ 11.5, 11.6, 14.2, 18.8, 25.5, 29.6, 42.4, 45.3, 60.1, 85.8, 102.3, 109.6, 117.5, 120.5, 120.6, 124.8, 126.8, 127.6, 128.7, 131.7, 136.7, 145.3, 165.1, 166.5, 209.1. HRMS (ESI) Calcd. for $C_{26}H_{26}NO_4$ $(M+H)^+$: 416.1856, Found: 416.1852.

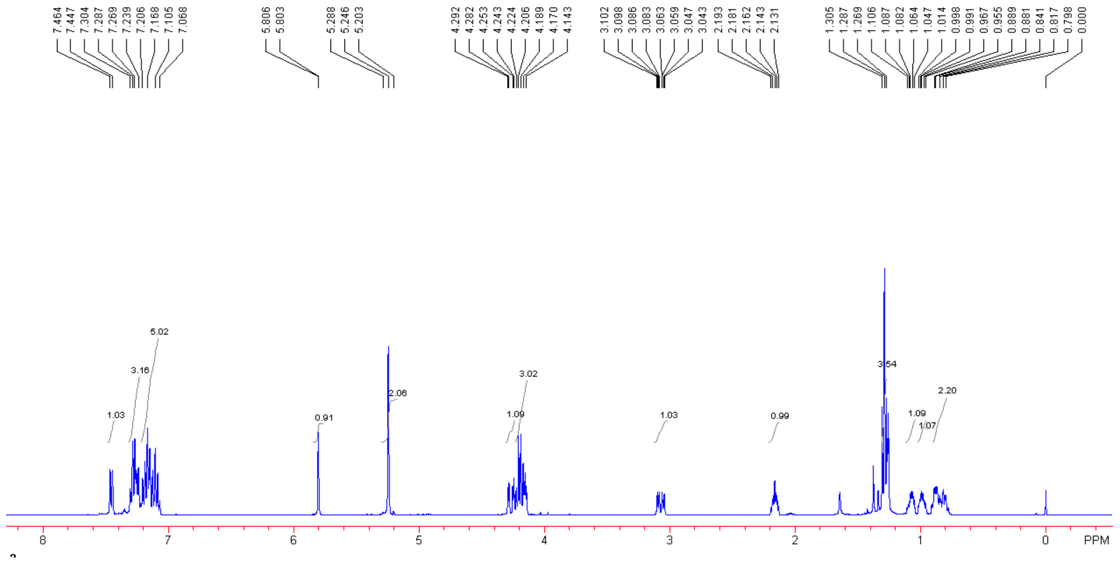

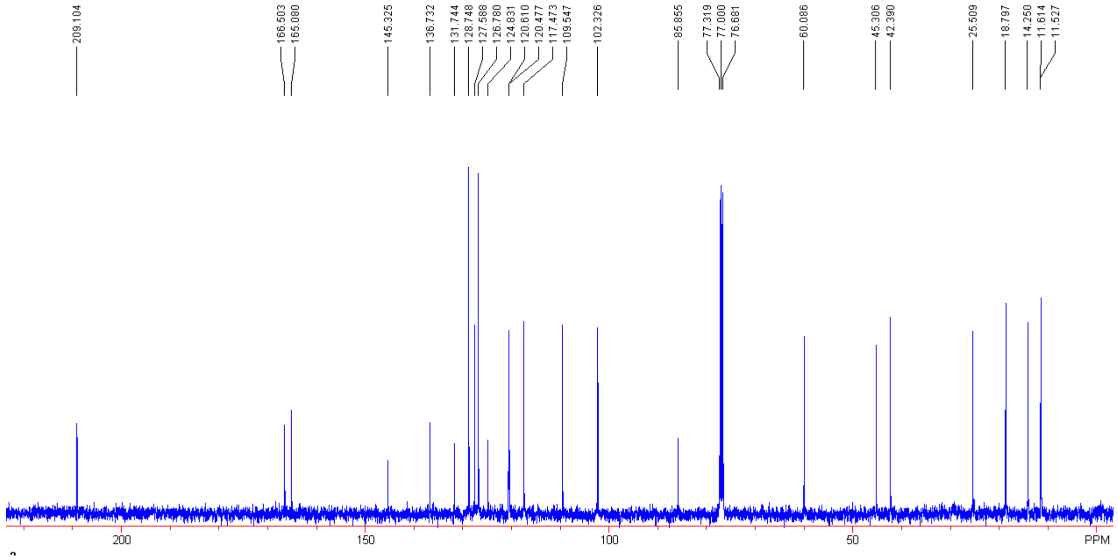

209.104
166.503
165.080
145.325
136.732
131.744
128.748
127.588
126.780
124.831
120.610
120.477
117.473
109.547
102.326
85.855
77.319
77.000
76.681
60.086
45.306
42.390
25.509
18.797
14.250
11.614
11.527
200
150
100
50
PPM

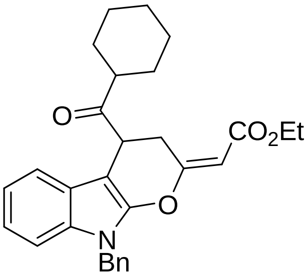

Compound 5b: Yield: 38 mg. 83%. A white solid. Mp: 142-144 °C. IR (neat) ν 2928, 1710, 1666, 1466, 1104, 740 cm^{-1}. ^{1}H NMR ($CDCl_3$, 400 MHz, TMS) δ 1.15-1.20 (m, 2H), 1.28 (t, *J* = 7.2 Hz, 3H), 1.33-1.44 (m, 3H), 1.65-1.72 (m, 3H), 1.79-1.82 (m, 1H), 1.92-1.95 (m, 1H), 2.70-2.77 (m, 1H), 2.86 (dd, *J* = 6.0 Hz, *J* = 15.6 Hz, 1H), 4.16-4.22 (m, 3H), 4.29 (dd, *J* = 2.8 Hz, *J* = 15.6 Hz, 1H), 5.23 (s, 2H), 5.80 (s, 1H), 7.06-7.18 (m, 5H), 7.22-7.29 (m, 3H), 7.38 (d, *J* = 8.0 Hz, 1H). ^{13}C NMR ($CDCl_3$, 100 MHz, TMS) δ 14.2, 25.4, 25.5, 25.7, 25.8, 28.2, 29.1, 39.6, 45.2, 48.9, 60.0, 85.7, 102.1, 109.6, 117.1, 120.4, 120.6, 124.7, 126.7, 127.5, 128.7, 131.6, 136.7, 145.5, 165.3, 166.6, 211.8. HRMS (ESI) Calcd. for $C_{29}H_{32}NO_4$ $(M+H)^+$: 458.2326, Found: 458.2322.

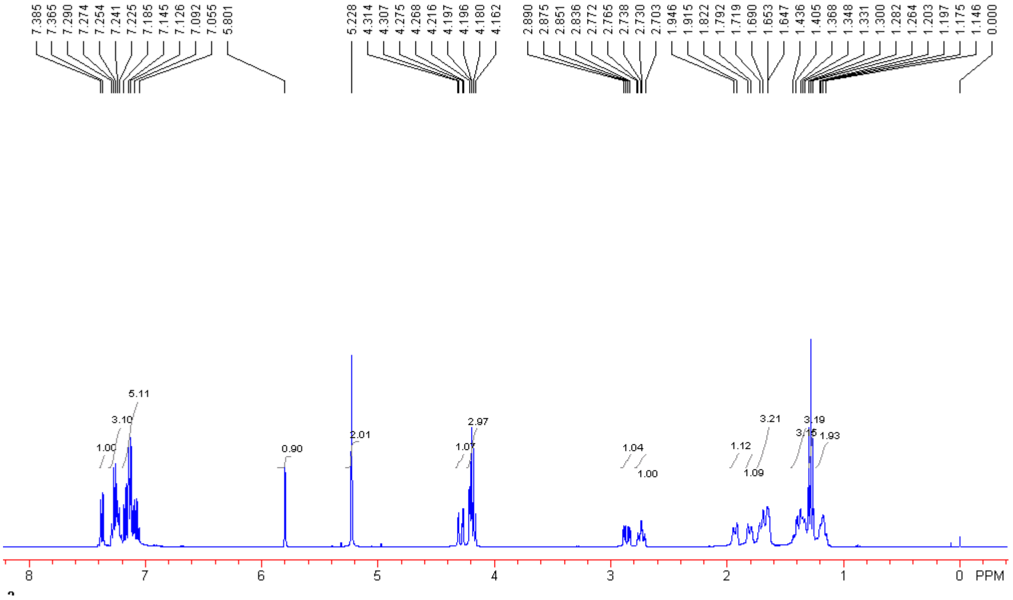

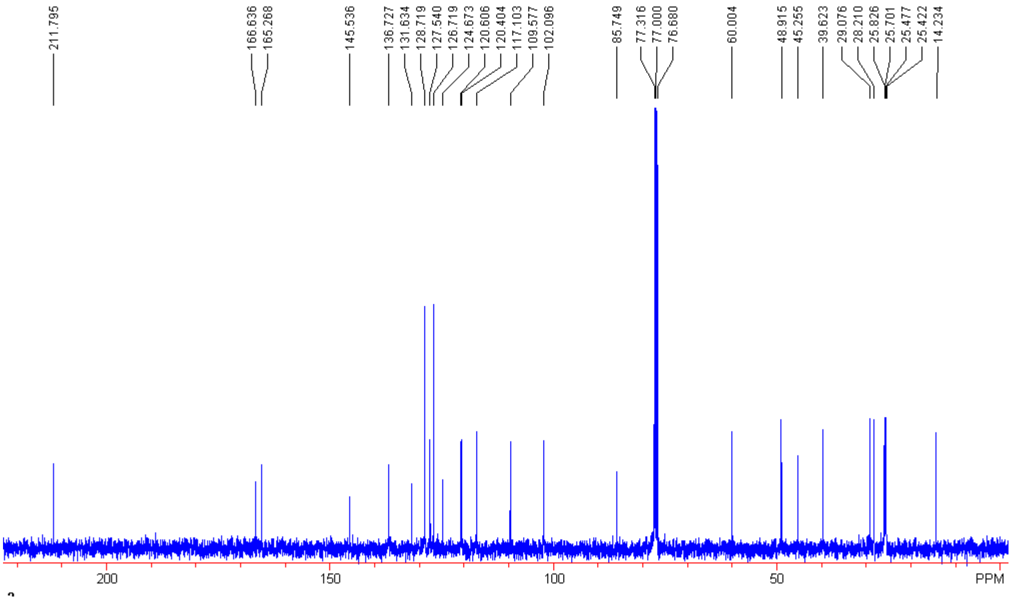
211.795
166.636
165.268
145.536
136.727
131.634
128.719
127.540
126.719
124.673
120.606
120.404
117.103
109.577
102.096
85.749
77.316
77.000
76.680
60.004
48.915
45.255
39.623
29.076
28.210
25.826
25.701
25.477
25.422
14.234
200
150
100
50
PPM

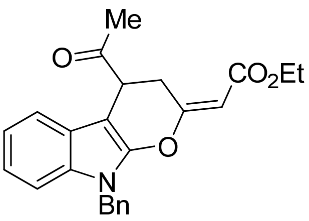

Compound 5c: Yield: 11 mg. 79%. A red solid. Mp: 118-120 °C. IR (neat) ν 2923, 1709, 1656, 1462, 1164, 740 cm^{-1}. ^{1}H NMR ($CDCl_3$, 400 MHz, TMS) δ 1.30 (t, *J* = 7.2 Hz, 3H), 2.24 (s, 3H), 2.86 (ddd, *J* = 1.6 Hz, *J* = 6.4 Hz, *J* = 15.6 Hz, 1H), 4.02 (dd, *J* = 2.8 Hz, *J* = 6.4 Hz, 1H), 4.21 (q, *J* = 7.2 Hz, 2H), 4.40 (dd, *J* = 2.8 Hz, *J* = 15.6 Hz, 1H), 5.25 (s, 2H), 5.82 (d, *J* = 1.6 Hz, 1H), 7.10-7.31 (m, 8H), 7.44 (d, *J* = 7.6 Hz, 1H). ^{13}C NMR ($CDCl_3$, 100 MHz, TMS) δ 14.3, 25.0, 28.0, 42.2, 45.3, 60.2, 85.4, 102.6, 109.6, 117.4, 120.6, 120.7, 124.7, 126.8, 127.6, 128.8, 131.7, 136.7, 145.3, 164.8, 166.6, 206.8. MS (ESI) *m/z* 390.1 $(M+H)^{+}$. HRMS (ESI) Calcd. for $C_{24}H_{24}NO_4$ $(M+H)^{+}$: 390.1700, Found: 390.1696.

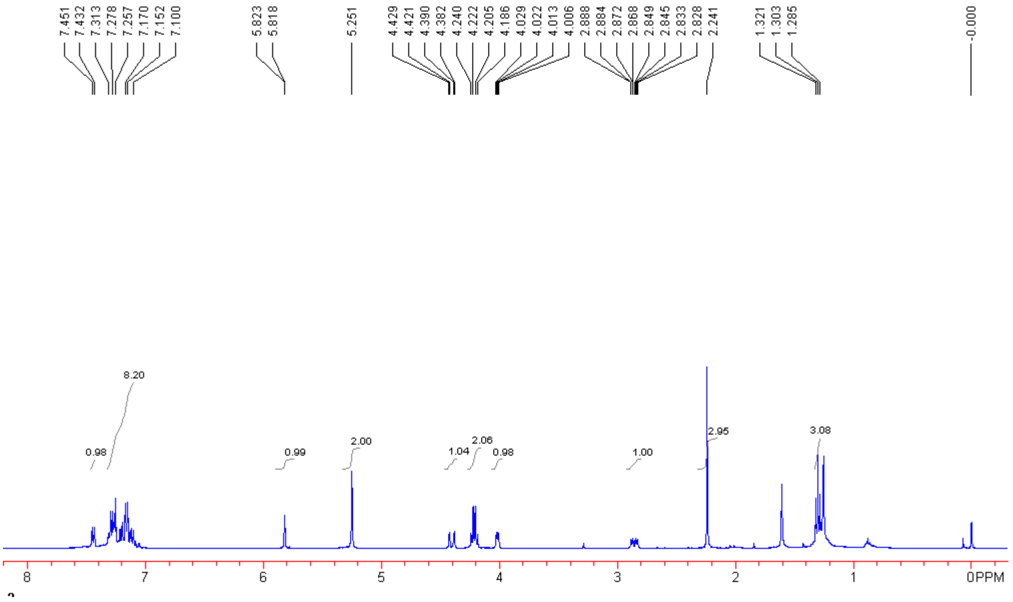

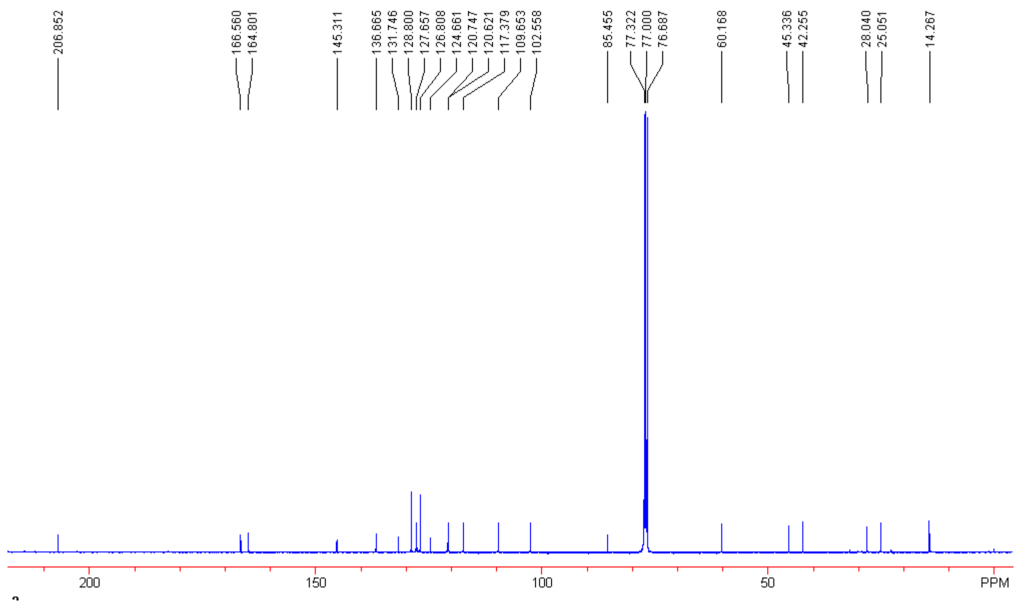

206.852
166.560
164.801
145.311
136.665
131.746
128.800
127.657
126.808
124.661
120.747
120.621
117.379
109.653
102.558
85.455
77.322
77.000
76.687
60.168
45.336
42.255
28.040
25.051
14.267
200
150
100
50
PPM

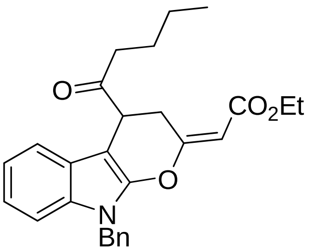

Compound 5d: Yield: 31 mg. 70%. A light red solid. Mp: 66-68 °C. IR (neat) ν 2926, 1703, 1644, 1462, 1115, 841 cm^{-1}. ^{1}H NMR ($CDCl_3$, 400 MHz, TMS) δ 0.82 (t, *J* = 7.2 Hz, 3H), 1.17-1.25 (m, 2H), 1.29 (t, *J* = 7.2 Hz, 3H), 1.47-1.55 (m, 2H), 2.52-2.66 (m, 2H), 2.81 (ddd, *J* = 2.0 Hz, *J* = 6.4 Hz, *J* = 15.6 Hz, 1H), 4.02 (dd, *J* = 2.8 Hz, *J* = 6.4 Hz, 1H), 4.20 (q, *J* = 7.2 Hz, 2H), 4.40 (dd, *J* = 2.8 Hz, *J* = 15.6 Hz, 1H), 5.24 (s, 2H), 5.81 (d, *J* = 2.0 Hz, 1H), 7.07-7.16 (m, 4H), 7.19 (d, *J* = 8.4 Hz, 1H), 7.24-7.30 (m, 3H), 7.43 (d, *J* = 8.0 Hz, 1H). ^{13}C NMR ($CDCl_3$, 100 MHz, TMS) δ 13.8, 14.2, 22.2, 25.2, 25.4, 40.2, 41.5, 45.3, 60.1, 85.6, 102.2, 109.6, 117.3, 120.5, 120.7, 124.7, 126.8, 127.6, 128.7, 131.7, 136.7, 145.3, 165.1, 166.6, 209.0. HRMS (ESI) Calcd. for $C_{27}H_{30}NO_4$ $(M+H)^+$: 432.2169, Found: 432.2165.

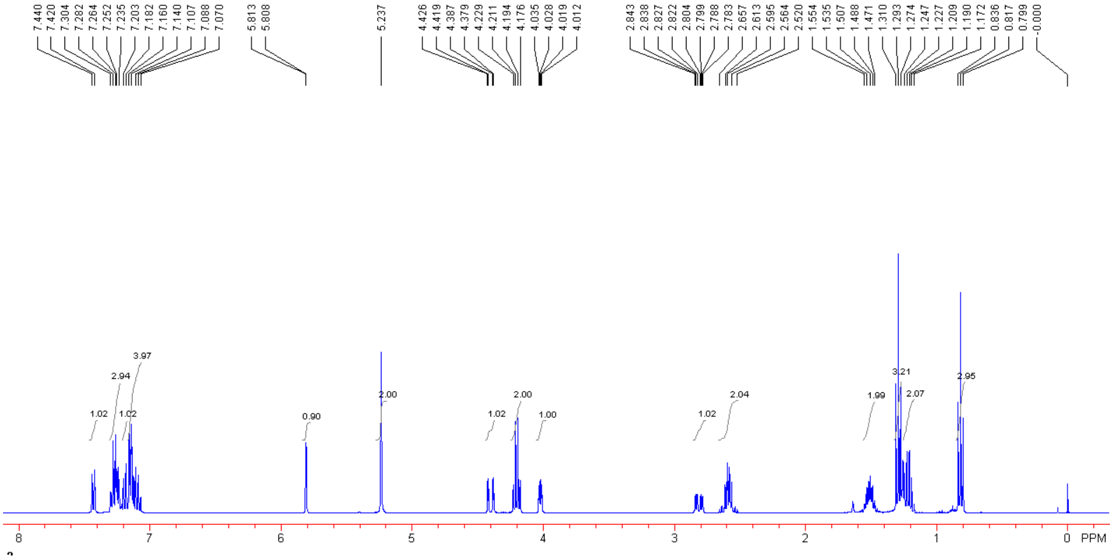

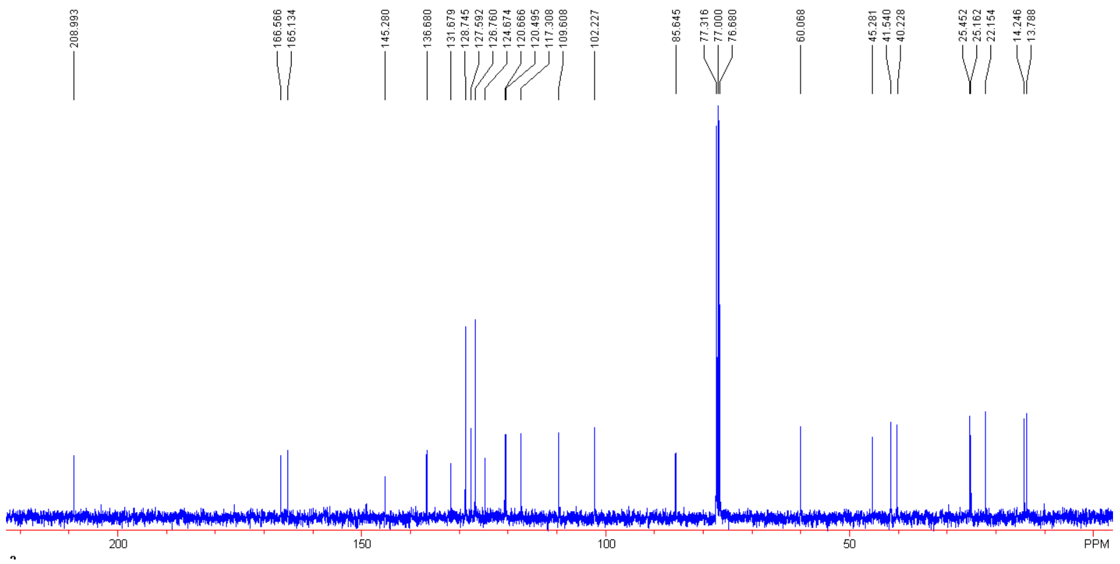
208.993
166.566
165.134
145.280
136.680
131.679
128.745
127.592
126.760
124.674
120.666
120.495
117.308
109.608
102.227
85.645
77.316
77.000
76.680
60.068
45.281
41.540
40.228
25.452
25.162
22.154
14.246
13.788
200
150
100
50
PPM

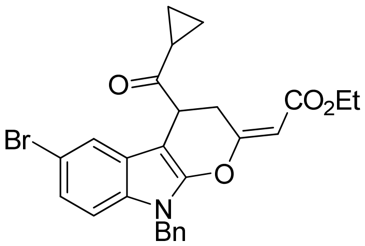

Compound 5e: Yield: 40 mg. 83%. A red solid. Mp: 141-143 °C. IR (neat) ν 2923, 1693, 1649, 1460, 1081, 847 cm^{-1}. ^{1}H NMR ($CDCl_3$, 400 MHz, TMS) δ 0.86-0.90 (m, 2H), 0.98-1.04 (m, 1H), 1.06-1.12 (m, 1H), 1.29 (t, J = 7.2 Hz, 3H), 2.09-2.15 (m, 1H), 3.09 (ddd, J = 1.6 Hz, J = 6.4 Hz, J = 15.6 Hz, 1H), 4.12 (dd, J = 4.0 Hz, J = 6.0 Hz, 1H), 4.17-4.28 (m, 3H), 5.20 (d, J = 16.8 Hz, 1H), 5.25 (d, J = 16.8 Hz, 1H), 5.81 (d, J = 1.6 Hz, 1H), 7.04 (d, J = 8.8 Hz, 1H), 7.12 (d, J = 6.4 Hz, 2H), 7.16 (dd, J = 2.0 Hz, J = 8.8 Hz, 1H), 7.27-7.31 (m, 3H), 7.55 (d, J = 1.2 Hz, 1H). ^{13}C NMR ($CDCl_3$, 100 MHz, TMS) δ 11.7, 11.8, 14.2, 19.0, 25.5, 42.2, 45.4, 60.2, 85.8, 102.8, 111.0, 113.9, 120.1, 123.3, 126.6, 126.7, 127.8, 128.8, 130.4, 136.3, 146.0, 164.6, 166.4, 208.7. HRMS (ESI) Calcd. for $C_{26}H_{25}BrNO_4$ $(M+H)^+$: 494.0961, Found: 494.0956.

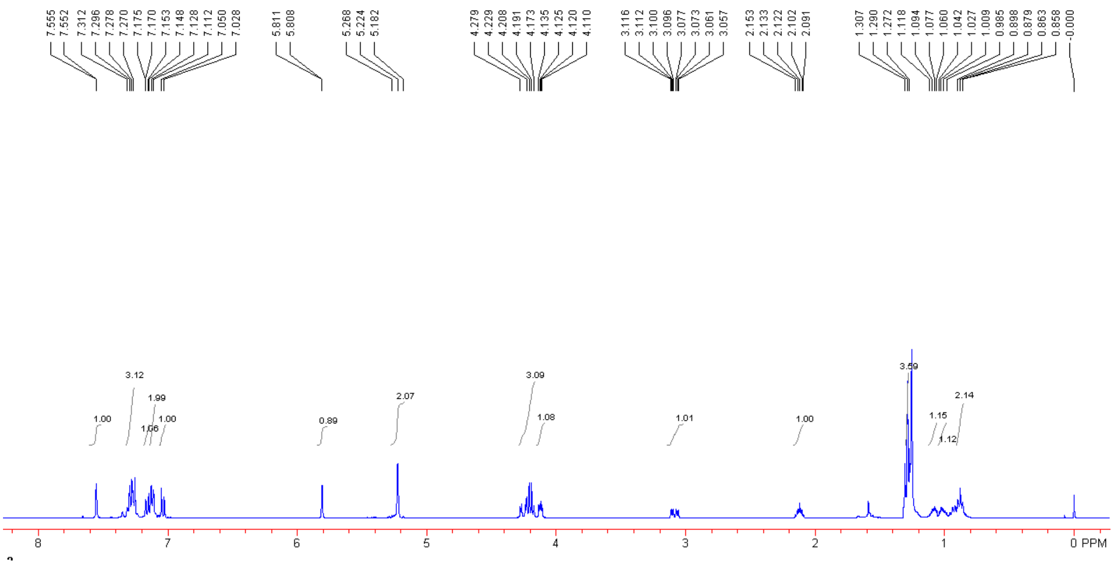

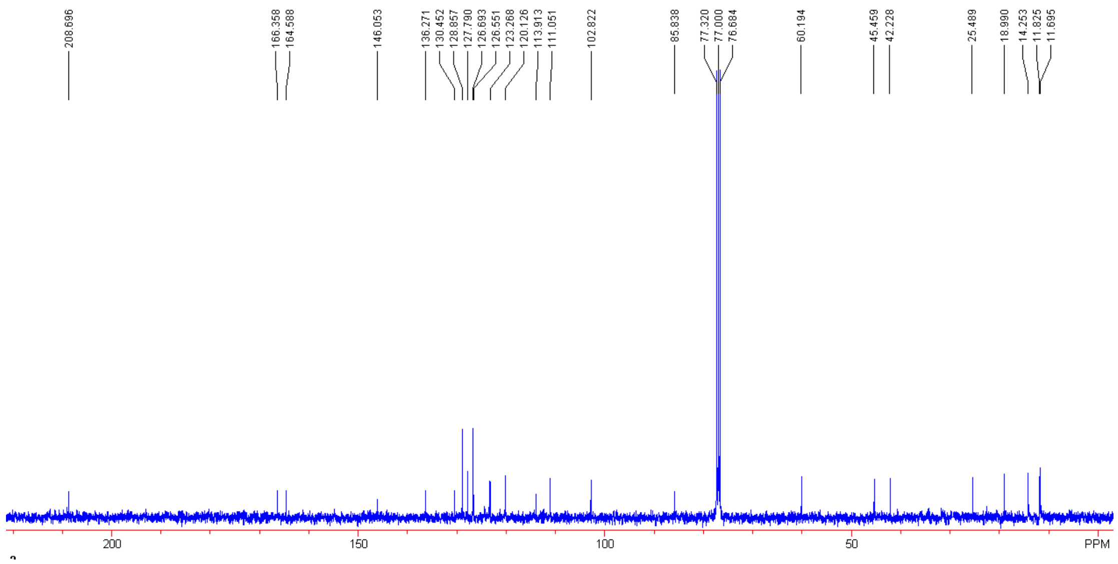
208.696
166.358
164.588
146.053
136.271
130.452
128.857
127.790
126.693
126.551
123.268
120.126
113.913
111.051
102.822
85.838
77.320
77.000
76.684
60.194
45.459
42.228
25.489
18.990
14.253
11.825
11.695
200
150
100
50
PPM

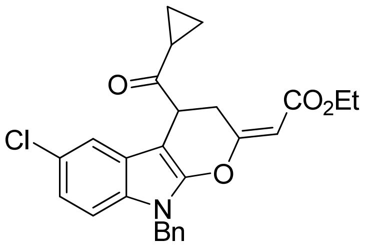

Compound 5f: Yield: 14 mg. 84%. A light red solid. Mp: 115-117 °C. IR (neat) ν 2925, 1695, 1662, 1475, 1116, 738 cm^{-1}. ^1H NMR ($CDCl_3$, 400 MHz, TMS) δ 0.85-0.94 (m, 2H), 0.98-1.04 (m, 1H), 1.06-1.12 (m, 1H), 1.29 (t, *J* = 7.2 Hz, 3H), 2.10-2.16 (m, 1H), 3.09 (ddd, *J* = 1.6 Hz, *J* = 6.4 Hz, *J* = 15.6 Hz, 1H), 4.12 (dd, *J* = 4.0 Hz, *J* = 6.4 Hz, 1H), 4.17-4.28 (m, 3H), 5.20 (d, *J* = 17.2 Hz, 1H), 5.24 (d, *J* = 17.2 Hz, 1H), 5.81 (d, *J* = 1.6 Hz, 1H), 7.02 (dd, *J* = 2.0 Hz, *J* = 8.8 Hz, 1H), 7.08 (d, *J* = 8.8 Hz, 1H), 7.13 (d, *J* = 8.0 Hz, 2H), 7.25-7.32 (m, 3H), 7.40 (d, *J* = 2.0 Hz, 1H). ^{13}C NMR ($CDCl_3$, 100 MHz, TMS) δ 11.7, 11.8, 14.2, 19.0, 25.5, 42.2, 45.4, 60.2, 85.9, 102.8, 110.6, 117.1, 120.6, 125.9, 126.4, 126.7, 127.8, 128.8, 130.1, 136.3, 146.2, 164.6, 166.4, 208.7. HRMS (ESI) Calcd. for $C_{26}H_{25}ClNO_4$ $(M+H)^+$: 450.1467, Found: 450.1474.

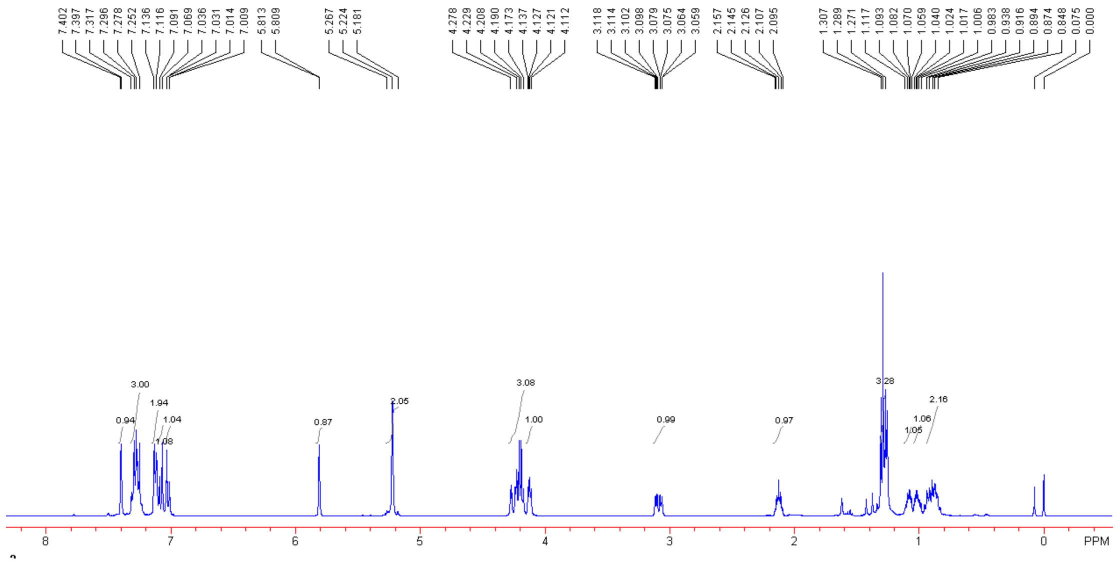

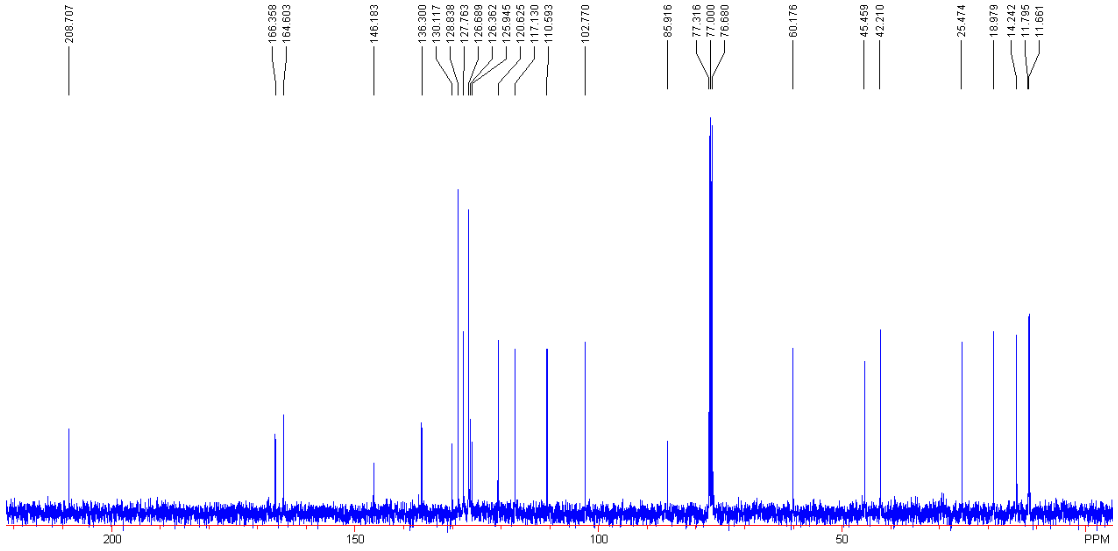
208.707
166.358
164.603
146.183
136.300
130.117
128.838
127.763
126.689
126.362
125.945
120.625
117.130
110.593
102.770
85.916
77.316
77.000
76.680
60.176
45.459
42.210
25.474
18.979
14.242
11.795
11.661
200
150
100
50
PPM

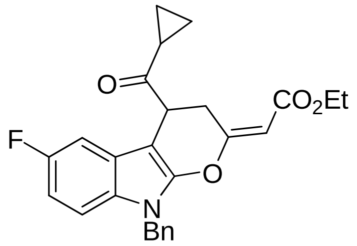

Compound 5g: Yield: 60 mg. 82%. A white solid. Mp: 114-116 °C. IR (neat) ν 2920, 1696, 1656, 1472, 1100, 701 cm^{-1}. ^{1}H NMR ($CDCl_3$, 400 MHz, TMS) δ 0.83-0.92 (m, 2H), 0.97-1.03 (m, 1H), 1.05-1.11 (m, 1H), 1.28 (t, *J* = 7.2 Hz, 3H), 2.10-2.16 (m, 1H), 3.11 (ddd, *J* = 1.2 Hz, *J* = 6.4 Hz, *J* = 15.6 Hz, 1H), 4.11 (dd, *J* = 4.0 Hz, *J* = 6.4 Hz, 1H), 4.17-4.26 (m, 3H), 5.19 (d, *J* = 17.2 Hz, 1H), 5.23 (d, *J* = 17.2 Hz, 1H), 5.81 (s, 1H), 6.79 (dt, *J* = 2.0 Hz, *J* = 9.2 Hz, 1H), 7.05-7.14 (m, 4H), 7.22-7.30 (m, 3H). ^{13}C NMR ($CDCl_3$, 100 MHz, TMS) δ 11.6, 11.7, 14.2, 18.9, 25.5, 42.2, 45.4, 60.1, 86.3 (d, *J* = 4.0 Hz), 102.6, 103.2 (d, *J* = 24.5 Hz), 108.2 (d, *J* = 25.7 Hz), 110.2 (d, *J* = 9.7 Hz), 125.3 (d, *J* = 10.4 Hz), 126.7, 127.7, 128.1, 128.8, 136.4, 146.4, 158.5 (d, *J* = 233.9 Hz), 164.7, 166.4, 208.8. ^{19}F NMR ($CDCl_3$, 376 MHz, CF_3COOH) δ -122.73 ~ -122.67 (m). HRMS (ESI) Calcd. for $C_{26}H_{25}FNO_4$ $(M+H)^+$: 434.1762, Found: 434.1759.

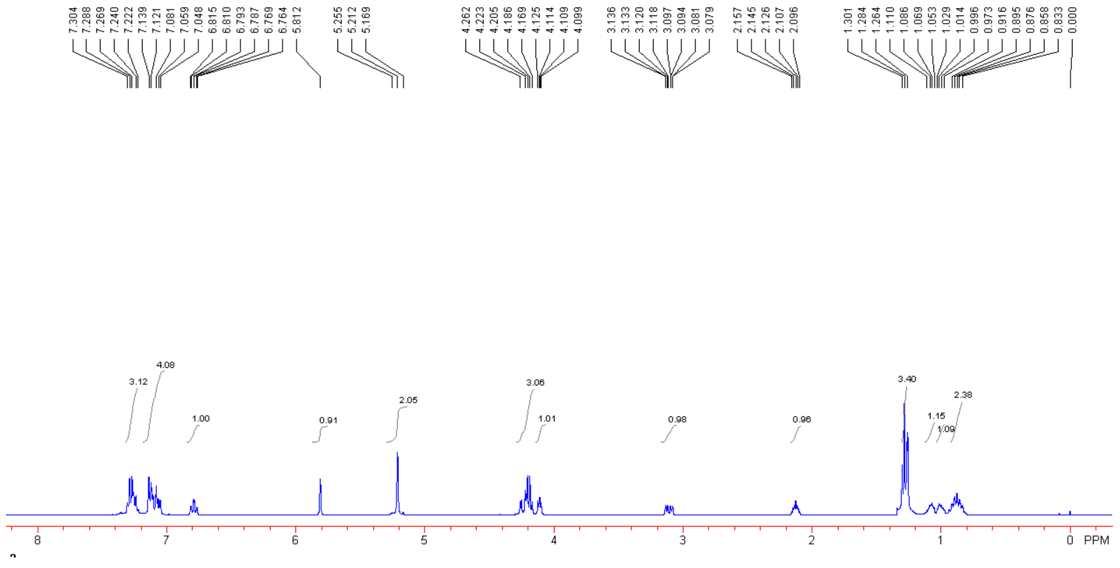

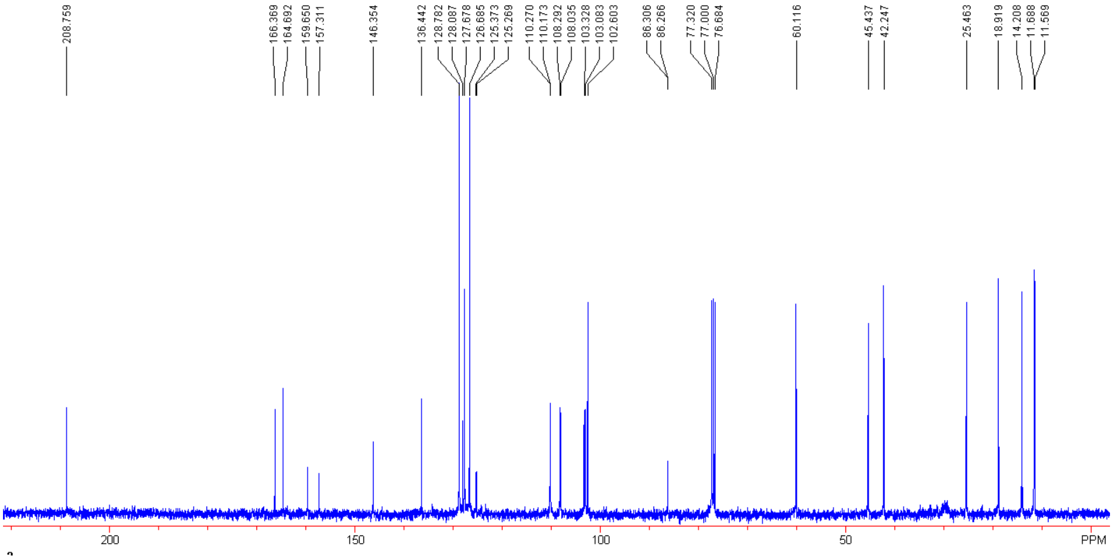
208.759
166.369
164.692
159.650
157.311
146.354
136.442
128.782
128.087
127.678
126.685
125.373
125.269
110.270
110.173
108.292
108.035
103.328
103.083
102.603
86.306
86.266
77.320
77.000
76.684
60.116
45.437
42.247
25.463
18.919
14.208
11.688
11.569
200
150
100
50
PPM

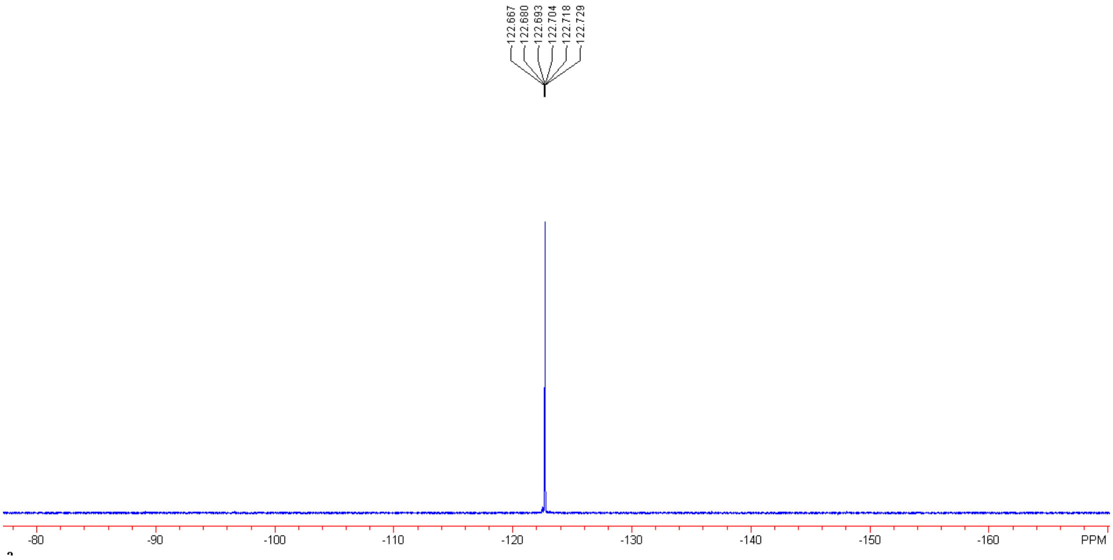
122.667
122.680
122.693
122.704
122.718
122.729
-80
-90
-100
-110
-120
-130
-140
-150
-160
PPM

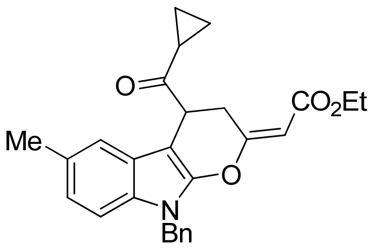

Compound 5h: Yield: 21 mg. 84%. A light red solid. Mp: 132-134 °C. IR (neat) ν 2923, 1704, 1649, 1466, 1116, 728 cm^{-1}. ^1H NMR ($CDCl_3$, 300 MHz, TMS) δ 0.80-0.88 (m, 2H), 0.96-1.02 (m, 1H), 1.04-1.11 (m, 1H), 1.28 (t, J = 7.2 Hz, 3H), 2.11-2.19 (m, 1H), 2.41 (s, 3H), 3.04 (dd, J = 6.3 Hz, J = 15.6 Hz, 1H), 4.11 (t, J = 4.2 Hz, 1H), 4.19 (q, J = 7.2 Hz, 2H), 4.28 (dd, J = 2.7 Hz, J = 15.6 Hz, 1H), 5.21 (s, 2H), 5.79 (s, 1H), 6.90 (d, J = 8.4 Hz, 1H), 7.07 (d, J = 8.1 Hz, 1H), 7.14 (d, J = 7.2 Hz, 1H), 7.22-7.29 (m, 4H). ^{13}C NMR ($CDCl_3$, 75 MHz, TMS) δ 11.5, 11.6, 14.2, 18.7, 21.5, 25.5, 42.2, 45.3, 60.0, 85.4, 102.2, 109.2, 117.4, 121.8, 125.0, 126.7, 127.5, 128.7, 130.0, 136.8, 145.3, 165.1, 166.5, 209.2. HRMS (ESI) Calcd. for $C_{27}H_{28}NO_4$ $(M+H)^+$: 430.2013, Found: 430.2009.

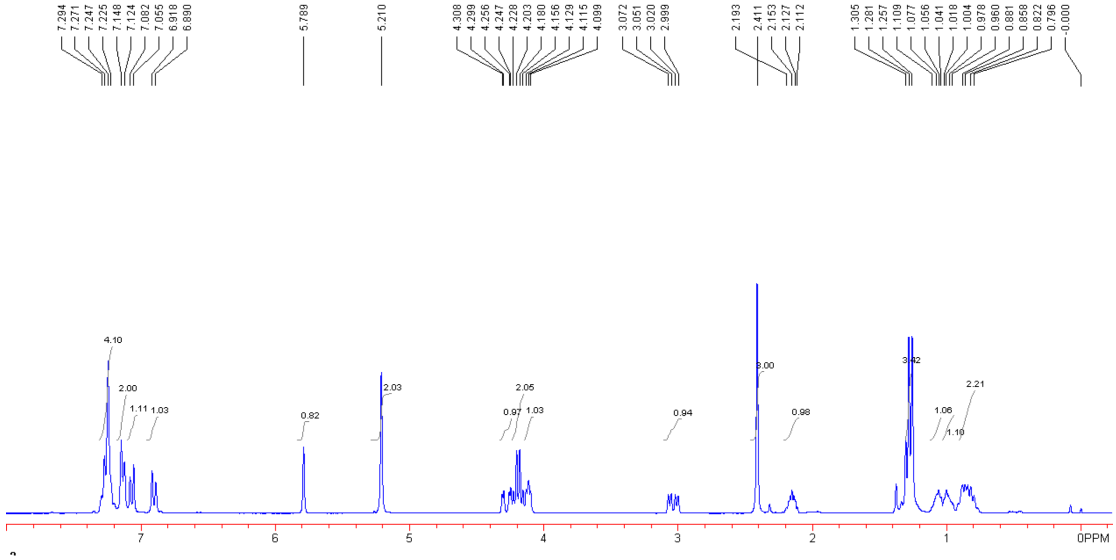

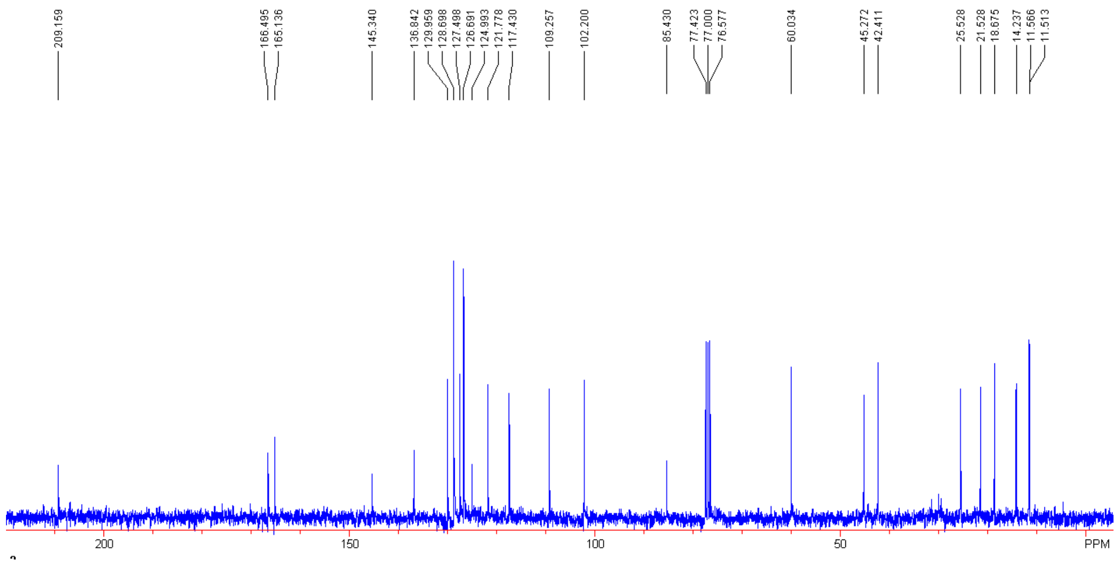

209.159
166.495
165.136
145.340
136.842
129.959
128.698
127.498
126.691
124.993
121.778
117.430
109.257
102.200
85.430
77.423
77.000
76.577
60.034
45.272
42.411
25.528
21.528
18.675
14.237
11.566
11.513
200
150
100
50
PPM

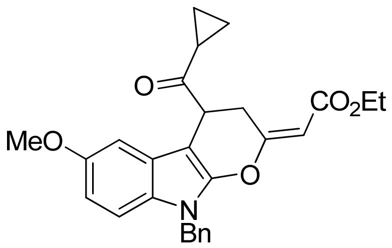

Compound 5i: Yield: 30 mg. 70%. A white solid. Mp: 143-145 °C. IR (neat) ν 2924, 1705, 1654, 1474, 1113, 704 cm^{-1}. ^{1}H NMR ($CDCl_3$, 400 MHz, TMS) δ 0.82-0.89 (m, 2H), 0.97-1.02 (m, 1H), 1.06-1.11 (m, 1H), 1.29 (t, J = 7.2 Hz, 3H), 2.11-2.17 (m, 1H), 3.11 (ddd, J = 1.6 Hz, J = 6.4 Hz, J = 15.6 Hz, 1H), 3.81 (s, 3H), 4.10 (dd, J = 4.0 Hz, J = 6.4 Hz, 1H), 4.17-4.26 (m, 3H), 5.19 (d, J = 16.8 Hz, 1H), 5.23 (d, J = 16.8 Hz, 1H), 5.79 (d, J = 1.2 Hz, 1H), 6.72 (dd, J = 2.4 Hz, J = 8.8 Hz, 1H), 6.93 (d, J = 2.4 Hz, 1H), 7.06 (d, J = 9.2 Hz, 1H), 7.14 (d, J = 6.4 Hz, 2H), 7.24-7.30 (m, 3H). ^{13}C NMR ($CDCl_3$, 100 MHz, TMS) δ 11.6, 14.2, 18.7, 25.6, 42.5, 45.4, 55.8, 60.1, 85.9, 100.9, 102.3, 109.1, 110.3, 125.4, 126.66, 126.71, 127.6, 128.7, 136.8, 145.7, 154.8, 165.0, 166.5, 209.2. HRMS (ESI) Calcd. for $C_{27}H_{28}NO_5$ $(M+H)^+$: 446.1962, Found: 446.1959.

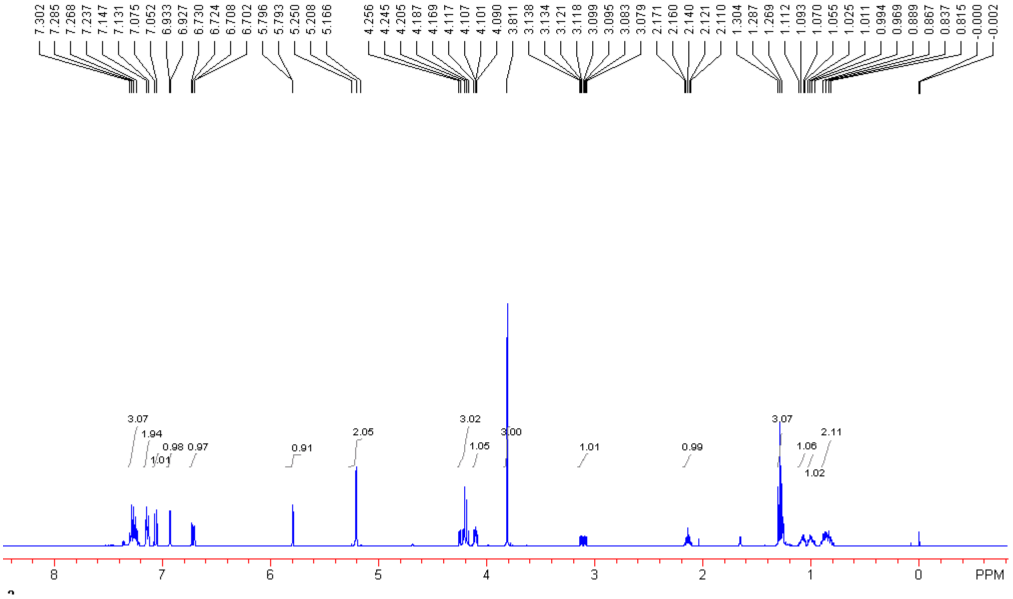

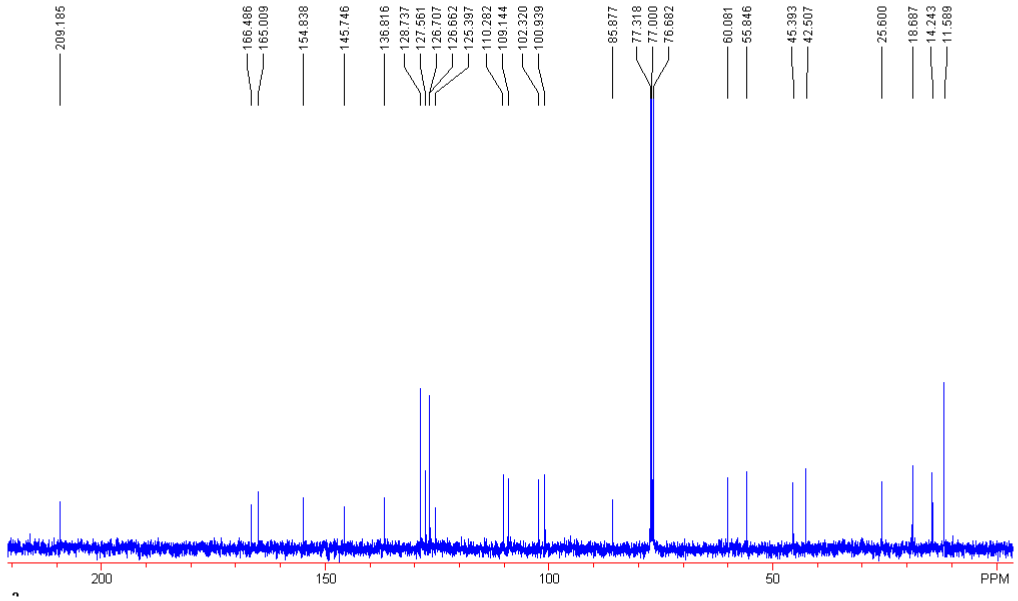

209.185
166.486
165.009
154.838
145.746
136.816
128.737
127.561
126.707
126.662
125.397
110.282
109.144
102.320
100.939
85.877
77.318
77.000
76.682
60.081
55.846
45.393
42.507
25.600
18.687
14.243
11.589
200
150
100
50
PPM

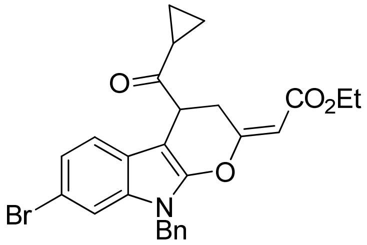

Compound 5j: Yield: 33 mg. 69%. A light yellow solid. Mp: 143-145 °C. IR (neat) ν 2926, 1694, 1652, 1465, 1119, 798 cm^{-1}. ^{1}H NMR ($CDCl_3$, 400 MHz, TMS) δ 0.82-0.90 (m, 2H), 0.96-1.02 (m, 1H), 1.05-1.10 (m, 1H), 1.29 (t, *J* = 7.2 Hz, 3H), 2.08-2.14 (m, 1H), 3.13 (ddd, *J* = 1.6 Hz, *J* = 6.4 Hz, *J* = 15.6 Hz, 1H), 4.13 (dd, *J* = 4.4 Hz, *J* = 6.4 Hz, 1H), 4.17-4.24 (m, 3H), 5.18 (d, *J* = 16.8 Hz, 1H), 5.23 (d, *J* = 16.8 Hz, 1H), 5.81 (d, *J* = 1.2 Hz, 1H), 7.13 (d, *J* = 7.6 Hz, 2H), 7.21 (dd, *J* = 1.6 Hz, *J* = 8.4 Hz, 1H), 7.26-7.33 (m, 5H). ^{13}C NMR ($CDCl_3$, 100 MHz, TMS) δ 11.6, 11.7, 14.2, 18.8, 25.4, 42.3, 45.4, 60.2, 86.2, 102.7, 112.5, 113.5, 118.8, 123.7, 123.8, 126.7, 127.8, 128.9, 132.6, 136.2, 145.6, 164.6, 166.4, 208.8. HRMS (ESI) Calcd. for $C_{26}H_{25}BrNO_4$ $(M+H)^+$: 494.0961, Found: 494.0959.

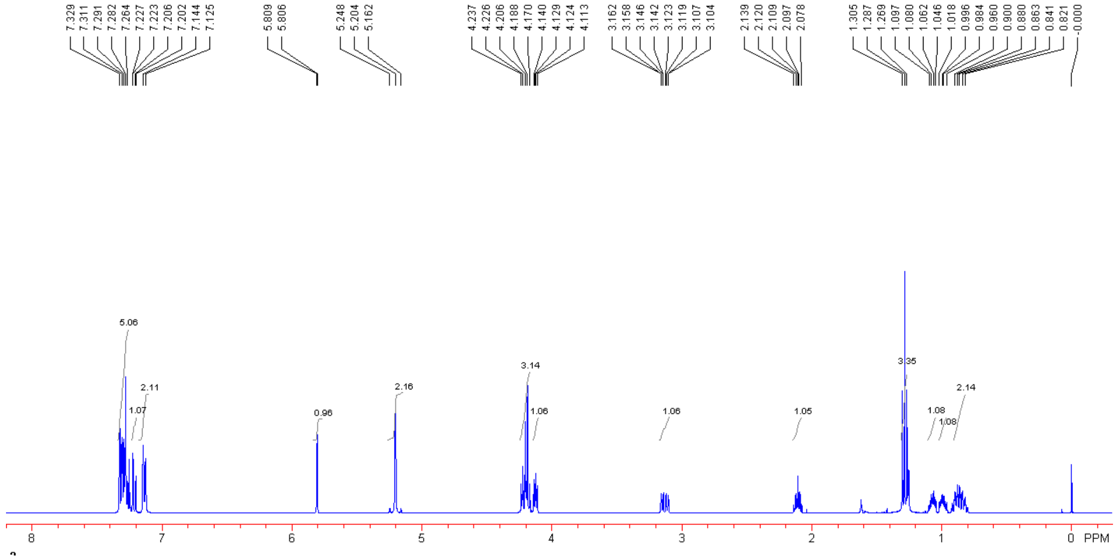

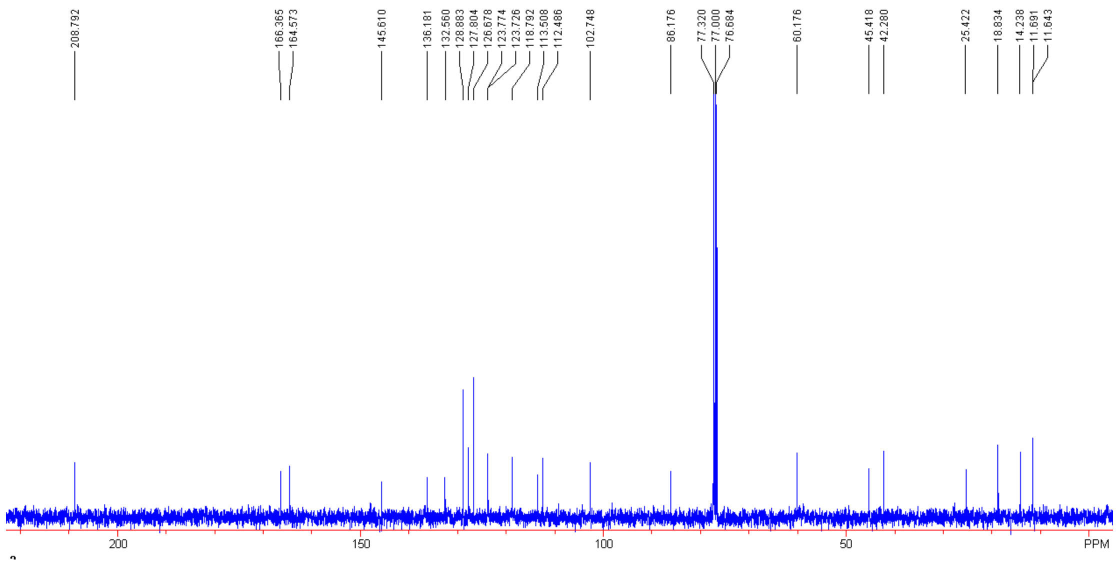
208.792
166.365
164.573
145.610
136.181
132.560
128.883
127.804
126.678
123.774
123.726
118.792
113.508
112.486
102.748
86.176
77.320
77.000
76.684
60.176
45.418
42.280
25.422
18.834
14.238
11.691
11.643
200
150
100
50
PPM

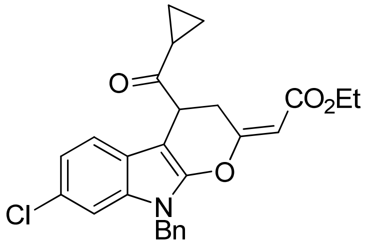

Compound 5k: Yield: 36 mg. 67%. A light yellow solid. Mp: 140-142 °C. IR (neat) ν 2925, 1703, 1658, 1467, 1111, 838 cm^{-1}. ^{1}H NMR ($CDCl_3$, 400 MHz, TMS) δ 0.82-0.90 (m, 2H), 0.96-1.02 (m, 1H), 1.05-1.10 (m, 1H), 1.28 (t, J = 7.2 Hz, 3H), 2.08-2.14 (m, 1H), 3.13 (dd, J = 6.0 Hz, J = 15.6 Hz, 1H), 4.12 (dd, J = 4.4 Hz, J = 6.0 Hz, 1H), 4.17-4.24 (m, 3H), 5.18 (d, J = 17.2 Hz, 1H), 5.22 (d, J = 17.2 Hz, 1H), 5.81 (s, 1H), 7.08 (d, J = 8.4 Hz, 1H), 7.14 (d, J = 7.2 Hz, 2H), 7.17 (s, 1H), 7.25-7.34 (m, 4H). ^{13}C NMR ($CDCl_3$, 100 MHz, TMS) δ 11.6, 11.7, 14.2, 18.8, 25.4, 42.3, 45.4, 60.1, 86.1, 102.7, 109.6, 118.4, 121.1, 123.4, 126.2, 126.7, 127.8, 128.8, 132.2, 136.2, 145.7, 164.6, 166.4, 208.8. HRMS (ESI) Calcd. for $C_{26}H_{24}ClNaNO_4$ $(M+Na)^+$: 472.1286, Found: 472.1278.

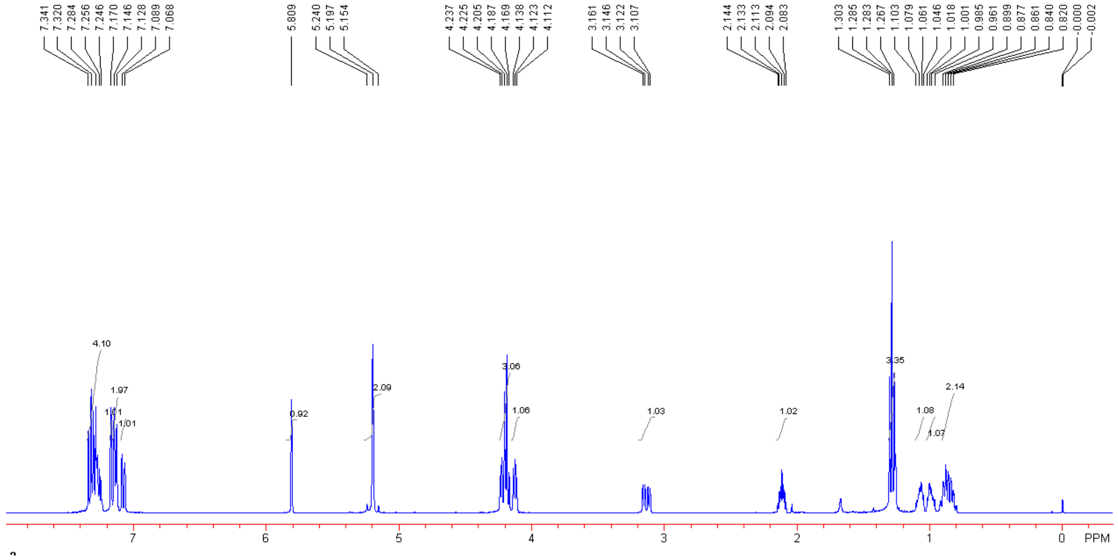

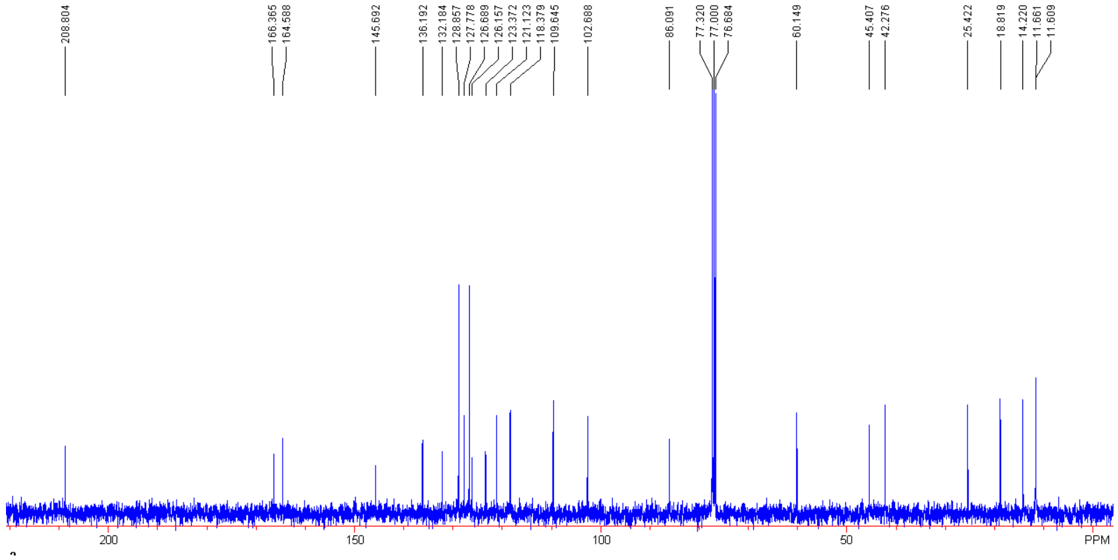
208.804
166.365
164.588
145.692
136.192
132.184
128.857
127.778
126.689
126.157
123.372
121.123
118.379
109.645
102.688
86.091
77.320
77.000
76.684
60.149
45.407
42.276
25.422
18.819
14.220
11.661
11.609
200
150
100
50
PPM

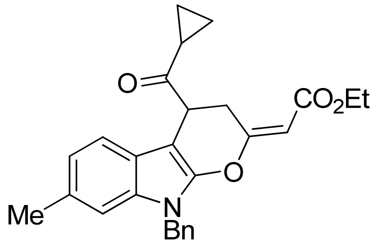

Compound 5l: Yield: 40 mg. 85%. A yellow solid. Mp: 138-140 °C. IR (neat) ν 2924, 1688, 1646, 1468, 1112, 702 cm^{-1}. ^{1}H NMR ($CDCl_3$, 400 MHz, TMS) δ 0.78-0.88 (m, 2H), 0.94-1.00 (m, 1H), 1.04-1.09 (m, 1H), 1.28 (t, J = 6.8 Hz, 3H), 2.12-2.18 (m, 1H), 2.40 (s, 3H), 3.06 (ddd, J = 1.6 Hz, J = 6.4 Hz, J = 15.6 Hz, 1H), 4.12 (dd, J = 4.0 Hz, J = 6.4 Hz, 1H), 4.16-4.27 (m, 3H), 5.21 (s, 2H), 5.78 (d, J = 1.2 Hz, 1H), 6.96 (d, J = 8.0 Hz, 1H), 7.00 (s, 1H), 7.15 (d, J = 6.8 Hz, 2H), 7.24-7.31 (m, 3H), 7.34 (d, J = 8.0 Hz, 1H). ^{13}C NMR ($CDCl_3$, 100 MHz, TMS) δ 11.5, 11.6, 14.2, 18.7, 21.8, 25.5, 42.4, 45.1, 60.0, 85.6, 102.1, 109.6, 117.2, 122.0, 122.5, 126.7, 127.5, 128.7, 130.3, 132.1, 136.9, 144.9, 165.2, 166.5, 209.1. HRMS (ESI) Calcd. for $C_{27}H_{28}NO_4$ $(M+H)^+$: 430.2013, Found: 430.2007.

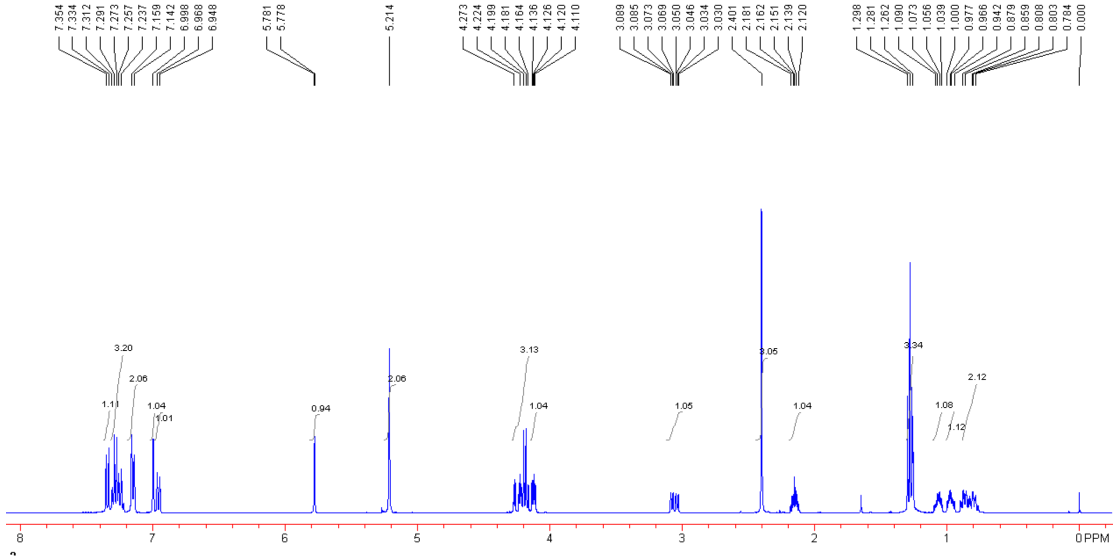

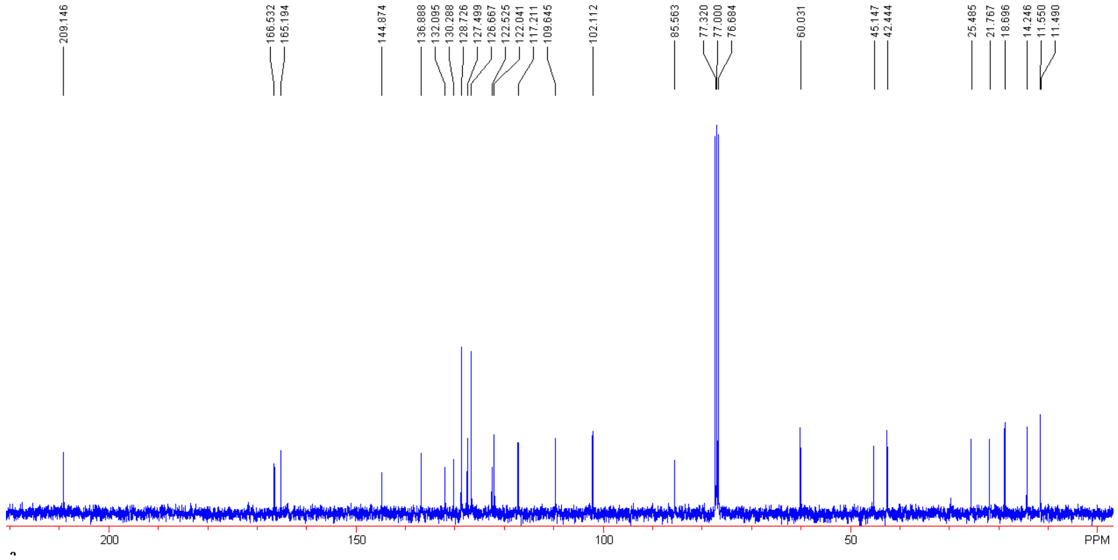
209.146
166.532
165.194
144.874
136.888
132.095
130.288
128.726
127.499
126.667
122.525
122.041
117.211
109.645
102.112
85.563
77.320
77.000
76.684
60.031
45.147
42.444
25.485
21.767
18.696
14.246
11.550
11.490
200
150
100
50
PPM

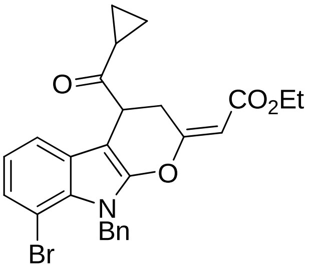

Compound 5m: Yield: 29 mg. 63%. A white solid. Mp: 110-112 °C. IR (neat) ν 2922, 1710, 1644, 1465, 1117, 727 cm^{-1}. ^{1}H NMR ($CDCl_3$, 400 MHz, TMS) δ 0.81-0.88 (m, 2H), 0.96-1.01 (m, 1H), 1.04-1.09 (m, 1H), 1.27 (t, *J* = 7.2 Hz, 3H), 2.06-2.12 (m, 1H), 3.08 (dd, *J* = 6.4 Hz, *J* = 15.6 Hz, 1H), 4.12 (dd, *J* = 4.0 Hz, *J* = 6.4 Hz, 1H), 4.18 (q, *J* = 7.2 Hz, 2H), 4.26 (dd, *J* = 3.2 Hz, *J* = 15.6 Hz, 1H), 5.68 (d, *J* = 16.8 Hz, 1H), 5.78 (s, 1H), 5.81 (d, *J* = 16.8 Hz, 1H), 6.97 (t, *J* = 8.0 Hz, 1H), 7.04 (d, *J* = 7.6 Hz, 2H), 7.22-7.28 (m, 4H), 7.37 (d, *J* = 7.6 Hz, 1H). ^{13}C NMR ($CDCl_3$, 100 MHz, TMS) δ 11.61, 11.65, 14.2, 18.7, 25.5, 42.2, 45.8, 60.2, 86.8, 102.9, 103.7, 116.7, 121.8, 125.99, 126.05, 127.2, 128.0, 128.5, 128.6, 138.2, 146.6, 164.4, 166.3, 208.7. HRMS (ESI) Calcd. for $C_{26}H_{25}BrNO_4$ $(M+H)^+$: 494.0961, Found: 494.0956.

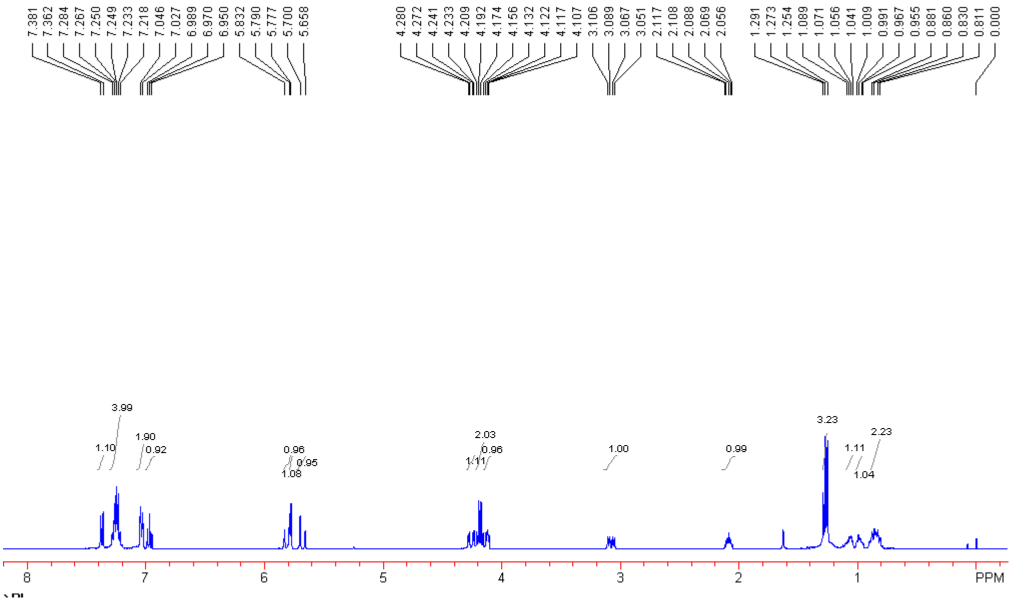

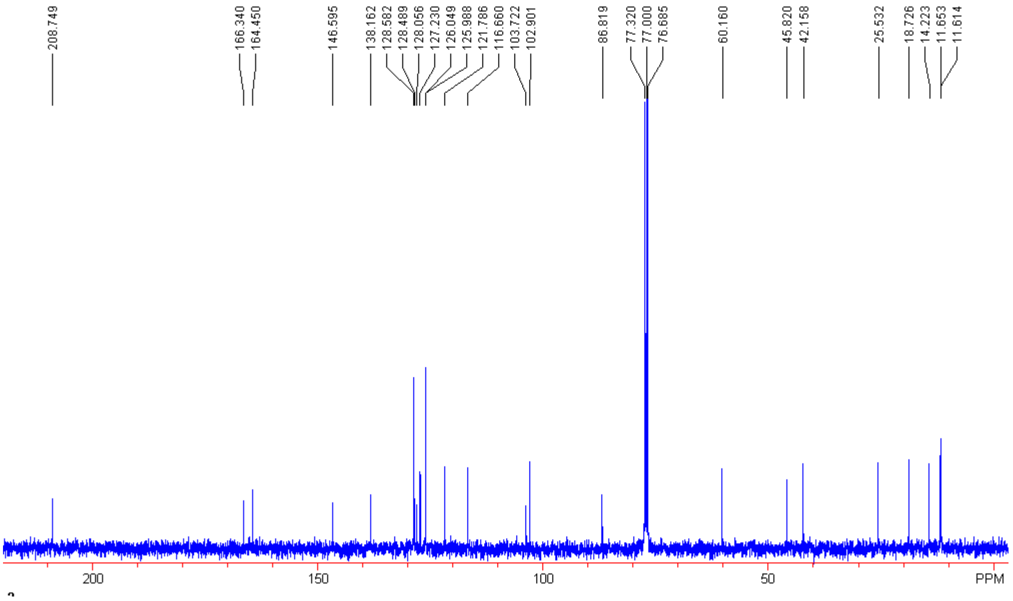
208.749
166.340
164.450
146.595
138.162
128.582
128.489
128.056
127.230
126.049
125.988
121.786
116.660
103.722
102.901
86.819
77.320
77.000
76.685
60.160
45.820
42.158
25.532
18.726
14.223
11.653
11.614
200
150
100
50
PPM

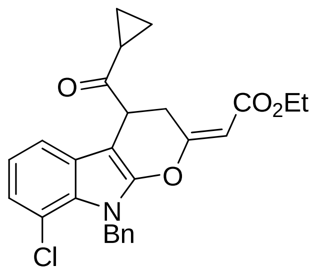

Compound 5n: Yield: 33 mg. 73%. A white solid. Mp: 111-113 °C. IR (neat) ν 2962, 1711, 1647, 1447, 1100, 781 cm^{-1}. ^{1}H NMR ($CDCl_3$, 400 MHz, TMS) δ 0.81-0.89 (m, 2H), 0.96-1.01 (m, 1H), 1.04-1.10 (m, 1H), 1.28 (t, *J* = 7.2 Hz, 3H), 2.07-2.13 (m, 1H), 3.08 (ddd, *J* = 1.6 Hz, *J* = 6.4 Hz, *J* = 15.6 Hz, 1H), 4.13 (dd, *J* = 4.0 Hz, *J* = 6.4 Hz, 1H), 4.19 (q, *J* = 7.2 Hz, 2H), 4.26 (dd, *J* = 4.0 Hz, *J* = 15.6 Hz, 1H), 5.64 (d, *J* = 16.8 Hz, 1H), 5.75 (d, *J* = 16.8 Hz, 1H), 5.79 (d, *J* = 1.6 Hz, 1H), 7.01-7.08 (m, 4H), 7.22-7.29 (m, 3H), 7.33 (dd, *J* = 2.0 Hz, *J* = 7.2 Hz, 1H). ^{13}C NMR ($CDCl_3$, 100 MHz, TMS) δ 11.64, 11.65, 14.2, 18.7, 25.5, 42.2, 46.3, 60.2, 86.9, 102.9, 116.1, 116.7, 121.4, 122.5, 126.2, 127.3, 127.8, 128.6, 138.2, 146.4, 164.5, 166.4, 208.8. HRMS (ESI) Calcd. for $C_{26}H_{25}ClNO_4$ $(M+H)^+$: 450.1467, Found: 450.1463.

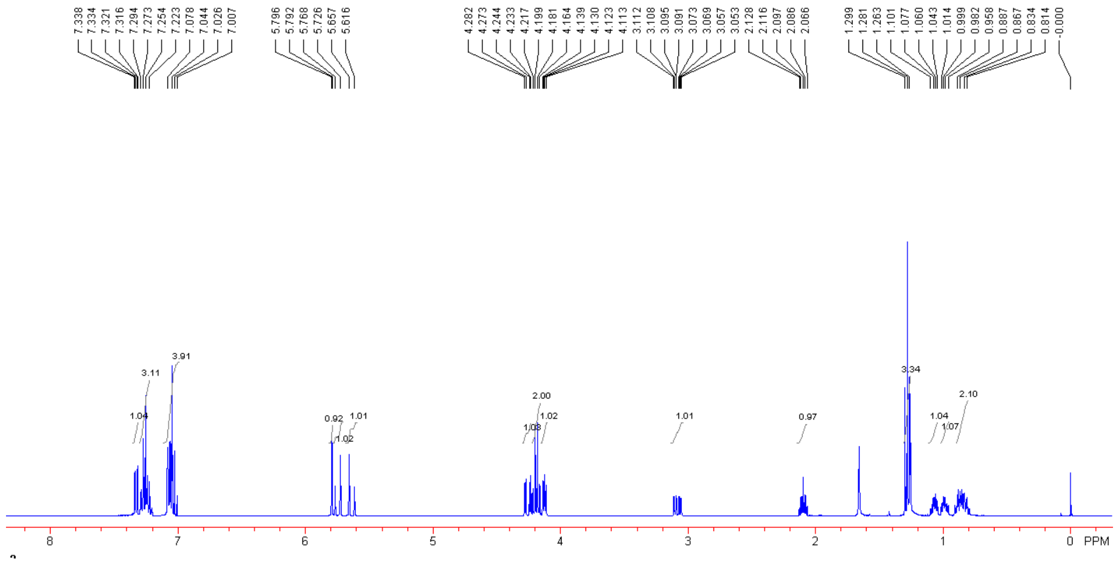

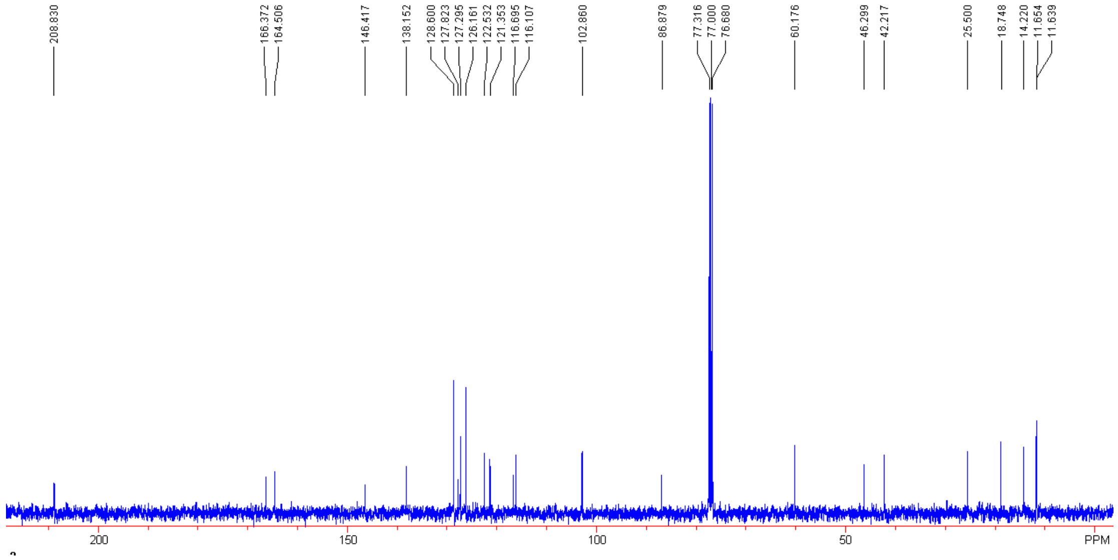
208.830
166.372
164.506
146.417
138.152
128.600
127.823
127.295
126.161
122.532
121.353
116.695
116.107
102.860
86.879
77.316
77.000
76.680
60.176
46.299
42.217
25.500
18.748
14.220
11.654
11.639
200
150
100
50
PPM

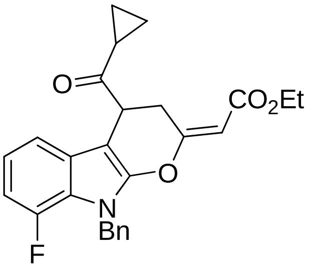

Compound 5o: Yield: 28 mg. 72%. A light red solid. Mp: 79-81 °C. IR (neat) ν 2924, 1691, 1600, 1456, 1098, 724 cm^{-1}. ^{1}H NMR ($CDCl_3$, 400 MHz, TMS) δ 0.80-0.89 (m, 2H), 0.95-1.01 (m, 1H), 1.04-1.10 (m, 1H), 1.29 (t, J = 7.2 Hz, 3H), 2.08-2.14 (m, 1H), 3.08 (ddd, J = 0.8 Hz, J = 6.4 Hz, J = 15.2 Hz, 1H), 4.12 (dd, J = 4.0 Hz, J = 6.4 Hz, 1H), 4.18-4.27 (m, 3H), 5.37 (d, J = 16.0 Hz, 1H), 5.42 (d, J = 16.0 Hz, 1H), 5.84 (s, 1H), 6.80 (dd, J = 7.6 Hz, J = 12.8 Hz, 1H), 6.98-7.03 (m, 1H), 7.16-7.30 (m, 6H). ^{13}C NMR ($CDCl_3$, 100 MHz, TMS) δ 11.59, 11.66, 14.2, 18.8, 25.4, 42.4, 47.2 (d, J = 4.8 Hz), 60.2, 87.1 (d, J = 1.7 Hz), 102.7, 106.9 (d, J = 18.2 Hz), 113.3 (d, J = 3.0 Hz), 119.0 (d, J = 9.6 Hz), 120.8 (d, J = 7.3 Hz), 127.0, 127.6, 128.4 (d, J = 5.6 Hz), 128.7, 137.4, 145.8, 149.8 (d, J = 241.7 Hz), 164.7, 166.4, 208.8. ^{19}F NMR ($CDCl_3$, 376 MHz, CF_3COOH) δ -134.86 ~ -134.81 (m). HRMS (ESI) Calcd. for $C_{26}H_{25}FNO_4$ $(M+H)^+$: 434.1762, Found: 434.1756.

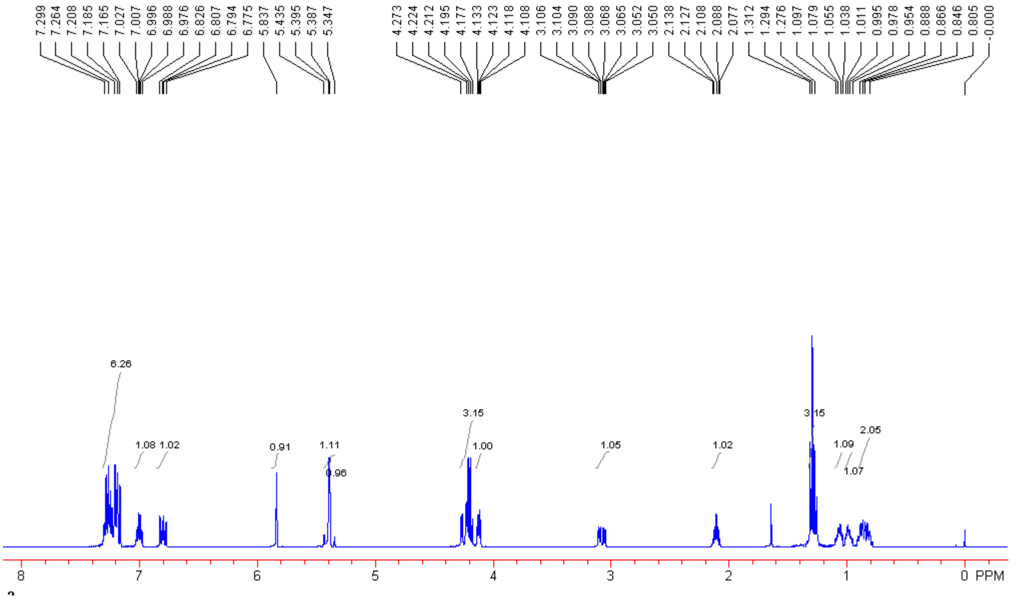

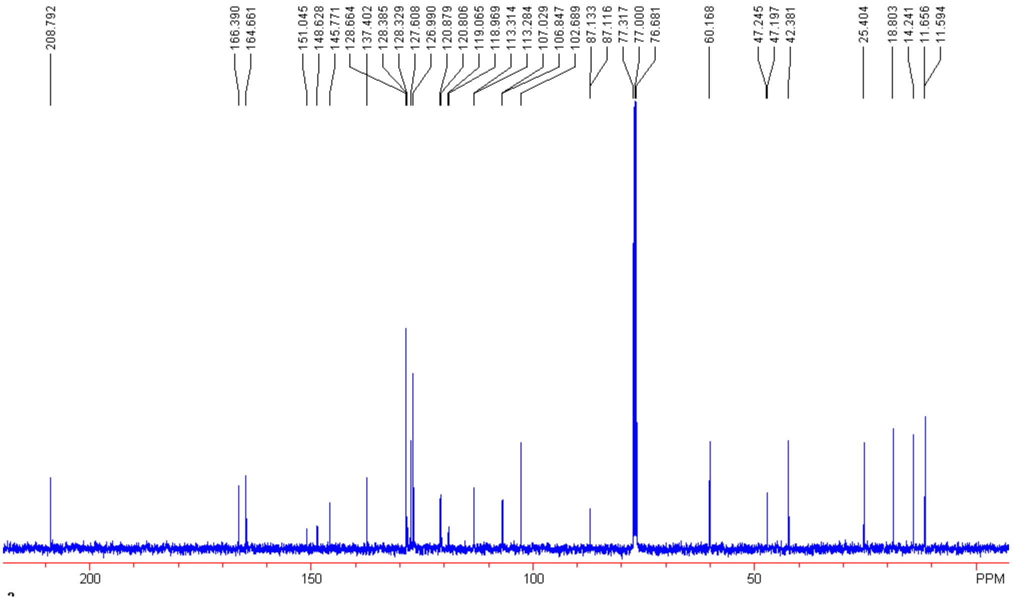
208.792
166.390
164.661
151.045
148.628
145.771
128.664
137.402
128.385
128.329
127.608
126.990
120.879
120.806
119.065
118.969
113.314
113.284
107.029
106.847
102.689
87.133
87.116
77.317
77.000
76.681
60.168
47.245
47.197
42.381
25.404
18.803
14.241
11.656
11.594
200
150
100
50
PPM

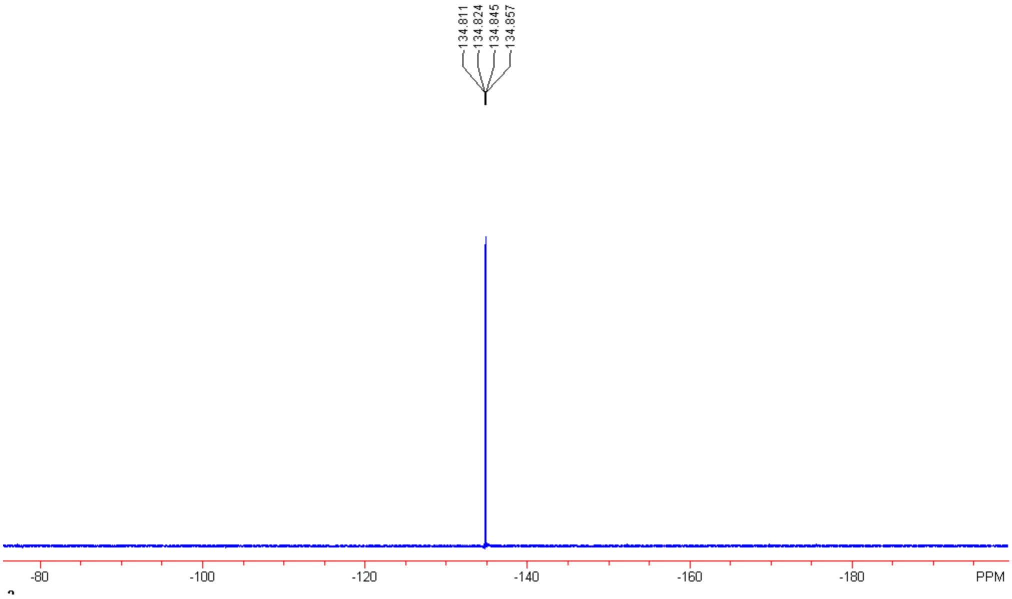
134.811
134.824
134.845
134.857
-80
-100
-120
-140
-160
-180
PPM

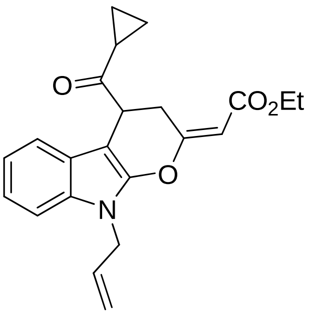

Compound 5p: Yield: 26 mg. 74%. A red solid. Mp: 70-72 °C. IR (neat) ν 2925, 1696, 1648, 1462, 1112, 732 cm^{-1}. ^1H NMR ($CDCl_3$, 400 MHz, TMS) δ 0.79-0.90 (m, 2H), 0.94-1.00 (m, 1H), 1.04-1.10 (m, 1H), 1.30 (t, J = 7.2 Hz, 3H), 2.12-2.18 (m, 1H), 3.05 (ddd, J = 1.6 Hz, J = 6.4 Hz, J = 15.2 Hz, 1H), 4.14 (dd, J = 4.0 Hz, J = 6.4 Hz, 1H), 4.18-4.28 (m, 3H), 4.67 (d, J = 5.2 Hz, 2H), 5.05 (dd, J = 1.2 Hz, J = 17.2 Hz, 1H), 5.17 (dd, J = 1.2 Hz, J = 10.4 Hz, 1H), 5.82 (d, J = 1.6 Hz, 1H), 5.88-5.98 (m, 1H), 7.12-7.15 (m, 2H), 7.21-7.24 (m, 1H), 7.44-7.46 (m, 1H). ^{13}C NMR ($CDCl_3$, 100 MHz, TMS) δ 11.5, 11.6, 14.3, 18.8, 25.5, 42.4, 44.0, 60.1, 85.7, 102.3, 109.5, 117.1, 117.4, 120.4, 120.5, 124.8, 131.7, 132.5, 145.2, 165.2, 166.6, 209.1. HRMS (ESI) Calcd. for $C_{22}H_{24}NO_4$ $(M+H)^+$: 366.1700, Found: 366.1698.

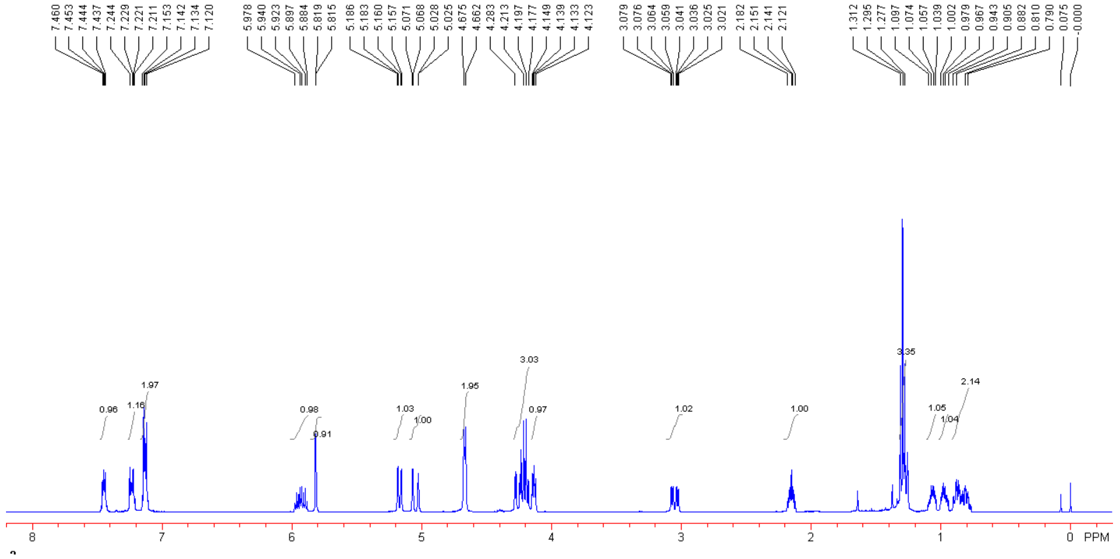

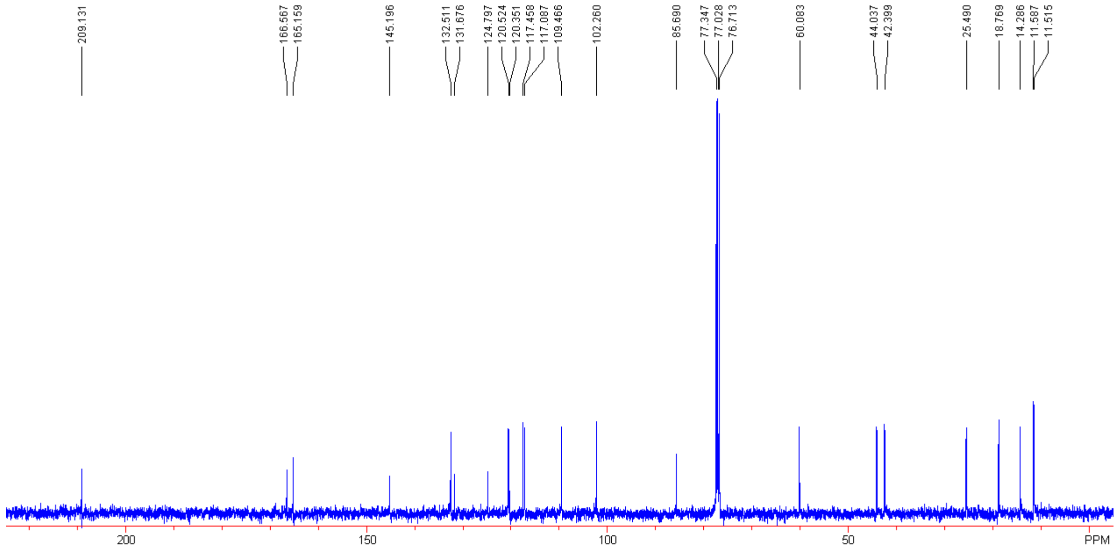
209.131
166.567
165.159
145.196
132.511
131.676
124.797
120.524
120.351
117.458
117.087
109.466
102.260
85.690
77.347
77.028
76.713
60.083
44.037
42.399
25.490
18.769
14.286
11.587
11.515
200
150
100
50
PPM

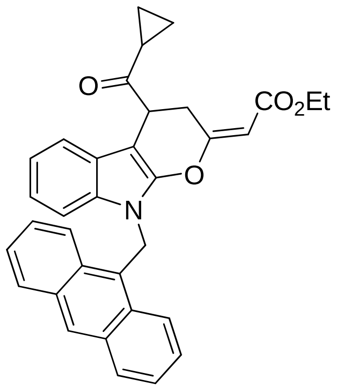

Compound 5q: Yield: 28 mg. 80%. A light red solid. Mp: 200-202 °C. IR (neat) ν 2926, 1700, 1614, 1463, 1112, 735 cm^{-1}. ^{1}H NMR ($CDCl_3$, 400 MHz, TMS) δ 0.73-0.82 (m, 2H), 0.91-0.96 (m, 1H), 1.01-1.06 (m, 1H), 1.28 (t, *J* = 7.2 Hz, 3H), 2.04-2.10 (m, 1H), 2.97 (ddd, *J* = 1.6 Hz, *J* = 6.4 Hz, *J* = 15.6 Hz, 1H), 4.07 (dd, *J* = 4.0 Hz, *J* = 6.4 Hz, 1H), 4.14-4.21 (m, 3H), 5.43 (s, 1H), 6.10 (s, 2H), 6.85 (t, *J* = 8.0 Hz, 1H), 6.92 (d, *J* = 8.4 Hz, 1H), 7.01 (t, *J* = 7.6 Hz, 1H), 7.37 (d, *J* = 8.0 Hz, 1H), 7.44-7.51 (m, 4H), 8.02 (d, *J* = 8.8 Hz, 2H), 8.32 (d, *J* = 8.8 Hz, 2H), 8.48 (s, 1H). ^{13}C NMR ($CDCl_3$, 100 MHz, TMS) δ 11.5, 14.3, 18.7, 25.4, 39.7, 42.3, 60.1, 86.1, 102.1, 110.2, 117.3, 120.3, 120.4, 123.6, 125.0, 126.1, 126.7, 129.0, 129.4, 130.9, 131.3, 132.1, 145.8, 164.9, 166.5, 209.1. HRMS (ESI) Calcd. for $C_{34}H_{30}NO_4$ $(M+H)^+$: 516.2169, Found: 516.2171.

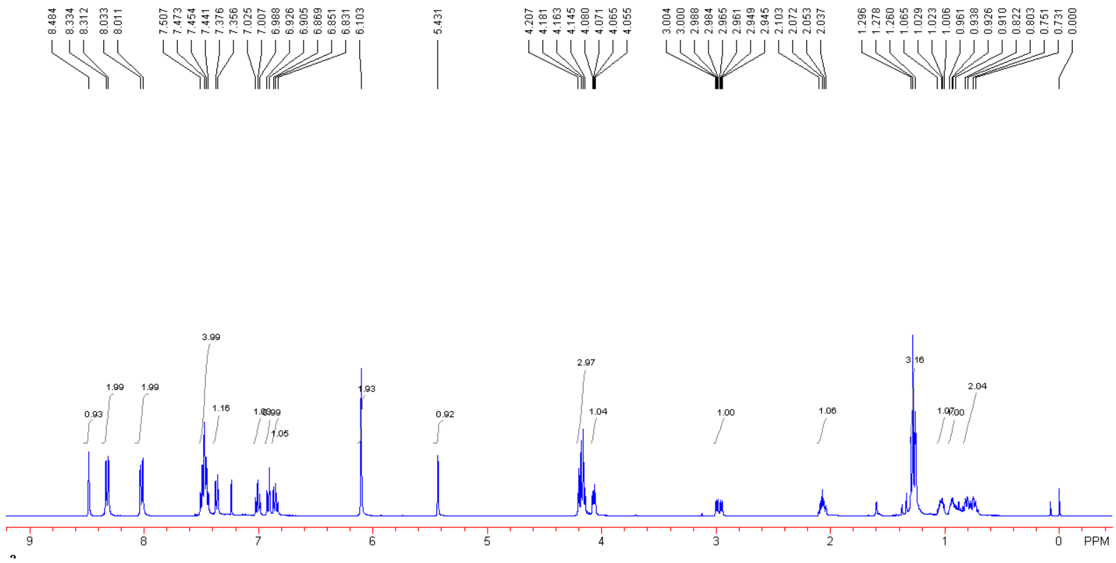

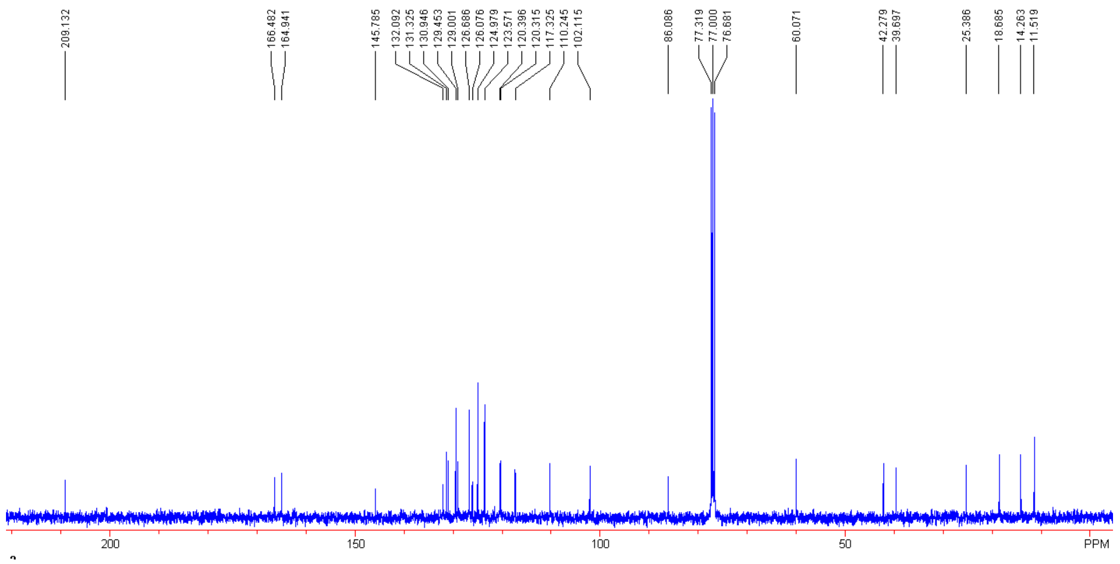
209.132
166.482
164.941
145.785
132.092
131.325
130.946
129.453
129.001
126.686
126.076
124.979
123.571
120.396
120.315
117.325
110.245
102.115
86.086
77.319
77.000
76.681
60.071
42.279
39.697
25.386
18.685
14.263
11.519
200
150
100
50
PPM

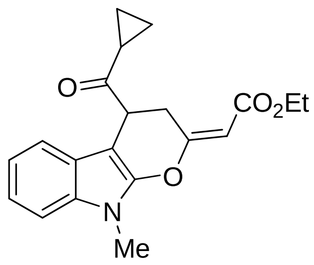

Compound 5r: Yield: 32 mg. 80%. A red oil. IR (neat) ν 2938, 1706, 1658, 1465, 1112, 737 cm^{-1}. ^1H NMR ($CDCl_3$, 400 MHz, TMS) δ 0.79-0.88 (m, 2H), 0.94-1.00 (m, 1H), 1.04-1.08 (m, 1H), 1.30 (t, *J* = 7.2 Hz, 3H), 2.12-2.19 (m, 1H), 3.03 (ddd, *J* = 1.6 Hz, *J* = 6.4 Hz, *J* = 15.6 Hz, 1H), 3.64 (s, 3H), 4.13 (dd, *J* = 4.0 Hz, *J* = 6.4 Hz, 1H), 4.18-4.29 (m, 3H), 5.83 (d, *J* = 1.6 Hz, 1H), 7.13-7.16 (m, 2H), 7.24 (d, *J* = 6.8 Hz, 1H), 7.44 (d, *J* = 7.2 Hz, 1H). ^{13}C NMR ($CDCl_3$, 100 MHz, TMS) δ 11.5, 11.6, 14.3, 18.8, 25.5, 27.7, 42.4, 60.1, 85.4, 102.1, 108.8, 117.3, 120.2, 120.4, 124.6, 132.2, 145.5, 165.2, 166.6, 209.2. HRMS (ESI) Calcd. for $C_{20}H_{22}NO_4$ $(M+H)^+$: 340.1543, Found: 340.1544.

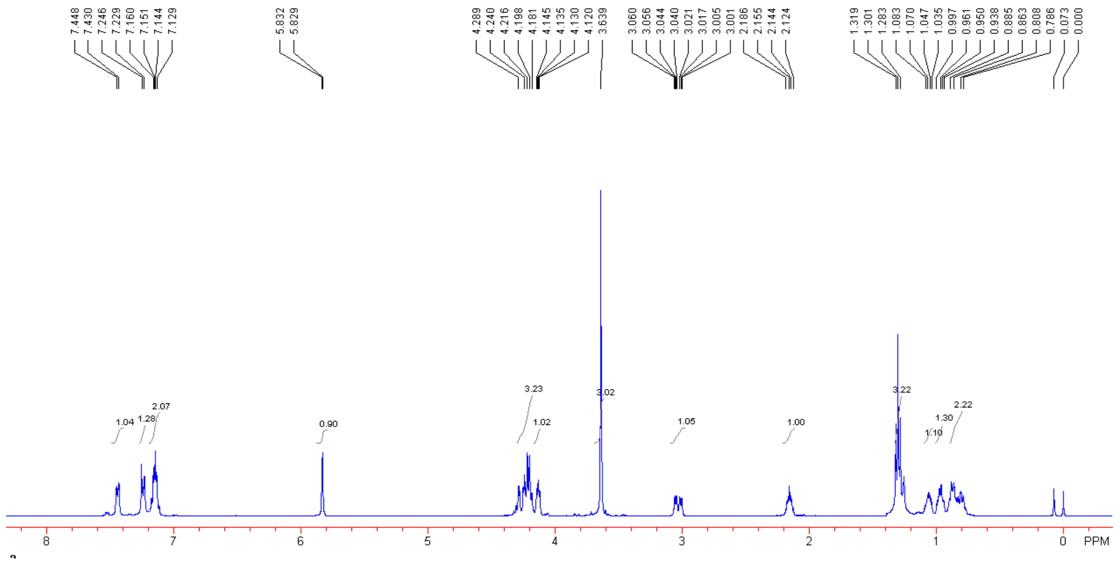

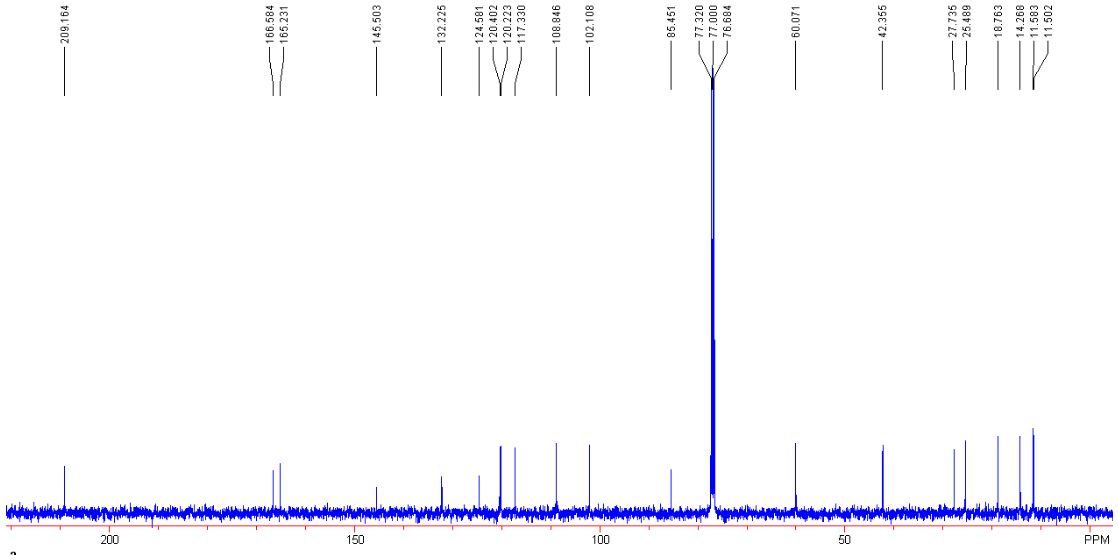
209.164
166.584
165.231
145.503
132.225
124.581
120.402
120.223
117.330
108.846
102.108
85.451
77.320
77.000
76.684
60.071
42.355
27.735
25.489
18.763
14.268
11.583
11.502
200
150
100
50
PPM

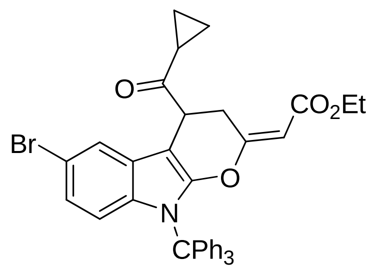

Compound 5s: Yield: 56 mg. 89%. A white solid. Mp: 196-198 °C. IR (neat) ν 2925, 1710, 1649, 1372, 1113, 698 cm^{-1}. ^1H NMR ($CDCl_3$, 400 MHz, TMS) δ 0.77-0.84 (m, 2H), 0.91-0.97 (m, 1H), 1.00-1.06 (m, 1H), 1.23 (t, J = 7.2 Hz, 3H), 1.90-1.96 (m, 1H), 2.80 (ddd, J = 2.0 Hz, J = 6.4 Hz, J = 15.6 Hz, 1H), 3.98 (dd, J = 3.6 Hz, J = 6.4 Hz, 1H), 4.08-4.13 (m, 3H), 4.86 (d, J = 1.6 Hz, 1H), 6.08 (d, J = 9.2 Hz, 1H), 6.80 (dd, J = 2.0 Hz, J = 8.8 Hz, 1H), 7.23-7.27 (m, 9H), 7.34-7.36 (m, 6H), 7.44 (d, J = 2.0 Hz, 1H). ^{13}C NMR ($CDCl_3$, 100 MHz, TMS) δ 11.5, 11.6, 14.2, 18.6, 24.9, 41.8, 60.0, 76.2, 88.5, 101.9, 114.0, 116.8, 119.4, 122.6, 127.3, 127.6, 127.9, 129.6, 131.9, 142.4, 148.2, 163.8, 166.4, 208.5. HRMS (ESI) Calcd. for $C_{38}H_{32}BrNaNO_4$ $(M+Na)^+$: 668.1407, Found: 668.1412.

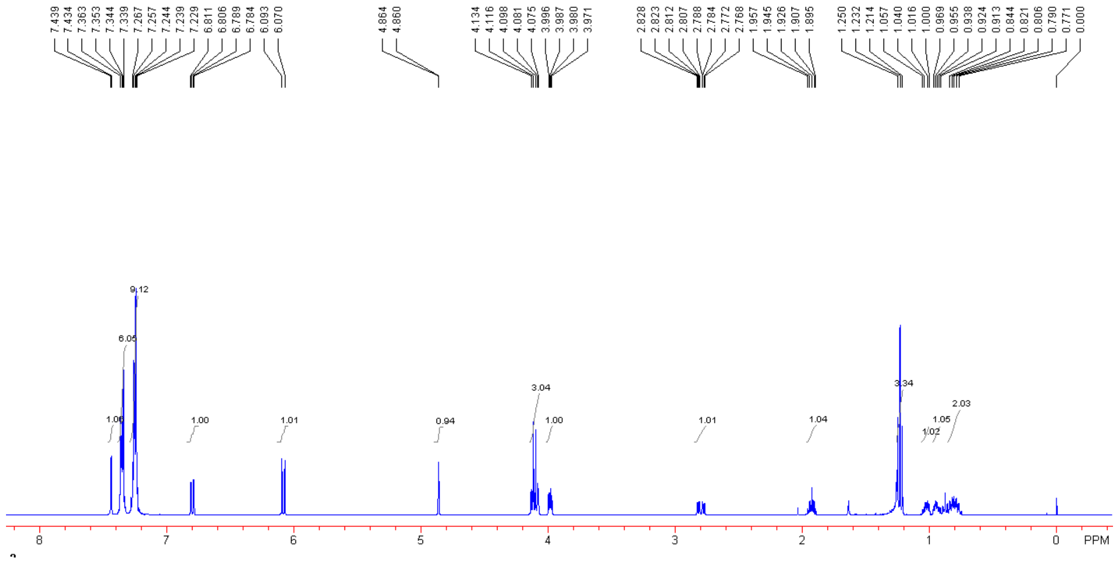

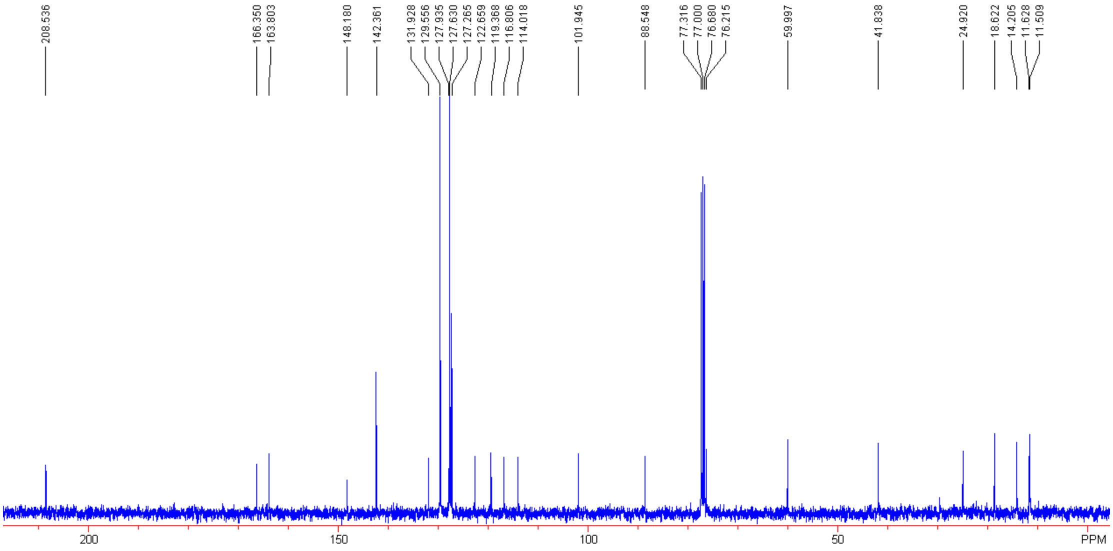

208.536
166.350
163.803
148.180
142.361
131.928
129.556
127.935
127.630
127.265
122.659
119.368
116.806
114.018
101.945
88.548
77.316
77.000
76.680
76.215
59.997
41.838
24.920
18.622
14.205
11.628
11.509
200
150
100
50
PPM

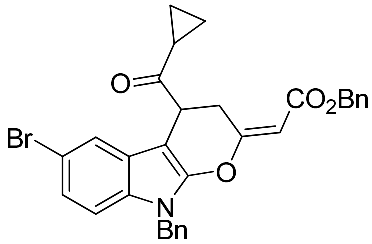

Compound 5t: Yield: 45 mg. 82%. A white solid. Mp: 161-163 °C. IR (neat) ν 2926, 1708, 1658, 1462, 1112, 733 cm^{-1}. ^{1}H NMR ($CDCl_3$, 400 MHz, TMS) δ 0.84-0.92 (m, 2H), 0.98-1.01 (m, 1H), 1.06-1.08 (m, 1H), 2.08-2.14 (m, 1H), 3.08 (dd, *J* = 6.0 Hz, *J* = 16.0 Hz, 1H), 4.12 (dd, *J* = 4.4 Hz, *J* = 6.0 Hz, 1H), 4.28 (dd, *J* = 4.4 Hz, *J* = 16.0 Hz, 1H), 5.14-5.23 (m, 4H), 5.87 (s, 1H), 7.04 (d, *J* = 8.8 Hz, 1H), 7.11 (d, *J* = 6.8 Hz, 2H), 7.16 (d, *J* = 8.4 Hz, 1H), 7.24-7.37 (m, 8H), 7.56 (s, 1H). ^{13}C NMR ($CDCl_3$, 100 MHz, TMS) δ 11.7, 11.9, 19.0, 25.5, 42.2, 45.4, 66.0, 85.8, 102.4, 111.1, 113.9, 120.1, 123.3, 126.5, 126.7, 127.8, 128.2, 128.5, 128.8, 130.4, 136.0, 136.2, 145.9, 165.2, 166.2, 208.6. HRMS (ESI) Calcd. for $C_{31}H_{27}BrNO_4$ $(M+H)^+$: 556.1118, Found: 556.1122.

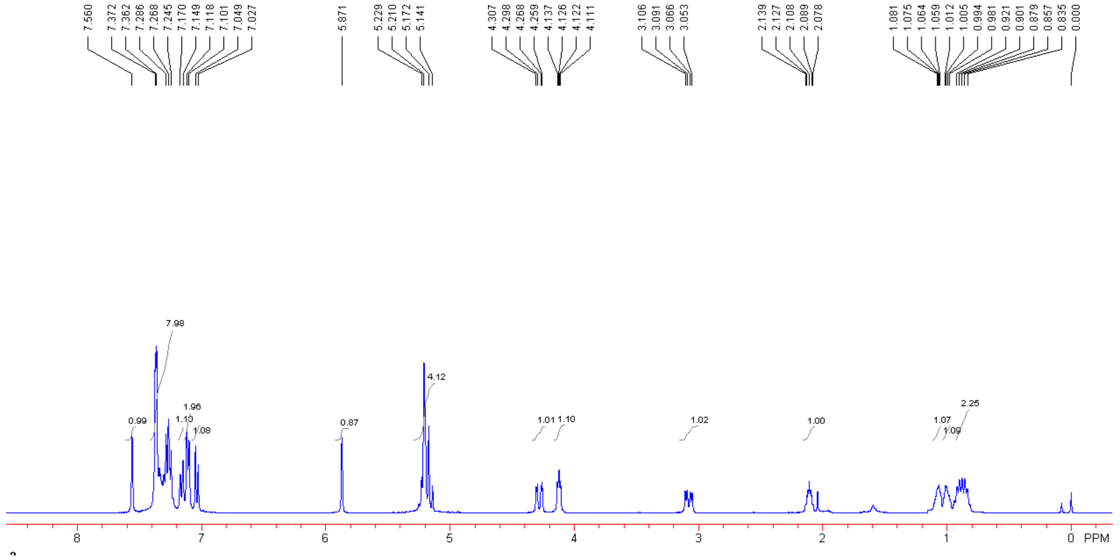

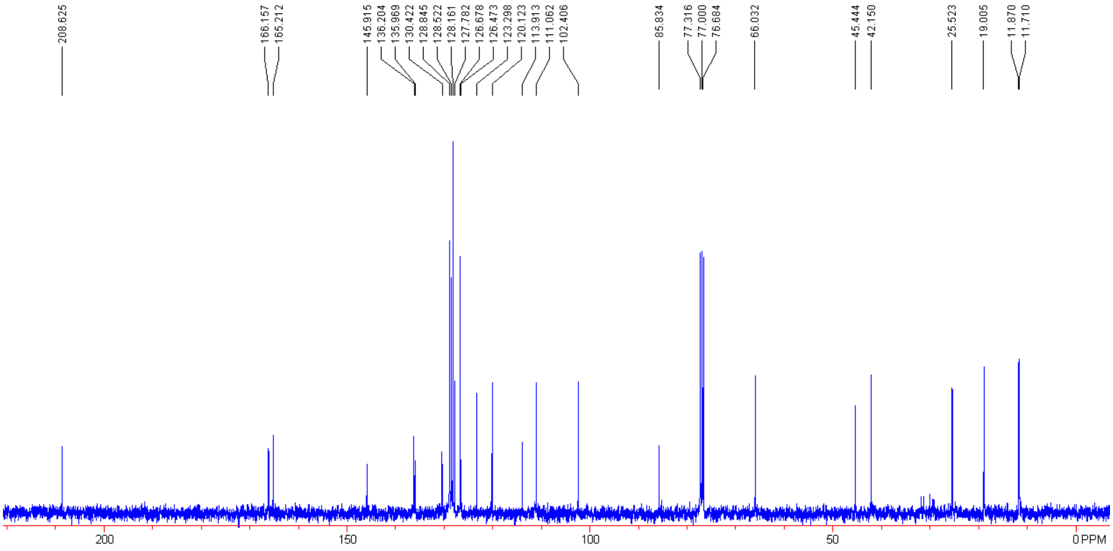
208.625
166.157
165.212
145.915
136.204
135.969
130.422
128.845
128.522
128.161
127.782
126.678
126.473
123.298
120.123
113.913
111.062
102.406
85.834
77.316
77.000
76.684
66.032
45.444
42.150
25.523
19.005
11.870
11.710
200
150
100
50
0 PPM

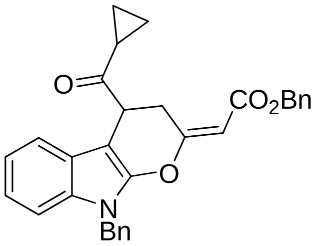

Compound 5u: Yield: 17 mg. 86%. A red solid. Mp: 50-52 ^{o}C. IR (neat) ν 2932, 1696, 1656, 1461, 1098, 735 cm^{-1}. ^{1}H NMR ($CDCl_3$, 400 MHz, TMS) δ 0.86-0.94 (m, 2H), 0.97-1.00 (m, 1H), 1.04-1.10 (m, 1H), 2.12-2.18 (m, 1H), 3.08 (ddd, *J* = 1.6 Hz, *J* = 6.4 Hz, *J* = 15.6 Hz, 1H), 4.16 (dd, *J* = 4.0 Hz, *J* = 6.4 Hz, 1H), 4.29 (dd, *J* = 4.0 Hz, *J* = 15.6 Hz, 1H), 5.14-5.28 (m, 4H), 5.86 (d, *J* = 1.6 Hz, 1H), 7.07-7.16 (m, 4H), 7.19-7.37 (m, 9H), 7.46 (d, *J* = 7.6 Hz, 1H). ^{13}C NMR ($CDCl_3$, 100 MHz, TMS) δ 11.56, 11.66, 18.8, 25.6, 42.4, 45.4, 66.0, 85.9, 102.0, 109.6, 117.5, 120.56, 120.65, 124.8, 126.8, 127.6, 128.1, 128.2, 128.5, 128.8, 131.8, 136.1, 136.7, 145.2, 165.7, 166.3, 209.0. HRMS (ESI) Calcd. for $C_{31}H_{28}NO_4$ $(M+H)^+$: 478.2013, Found: 478.2005.

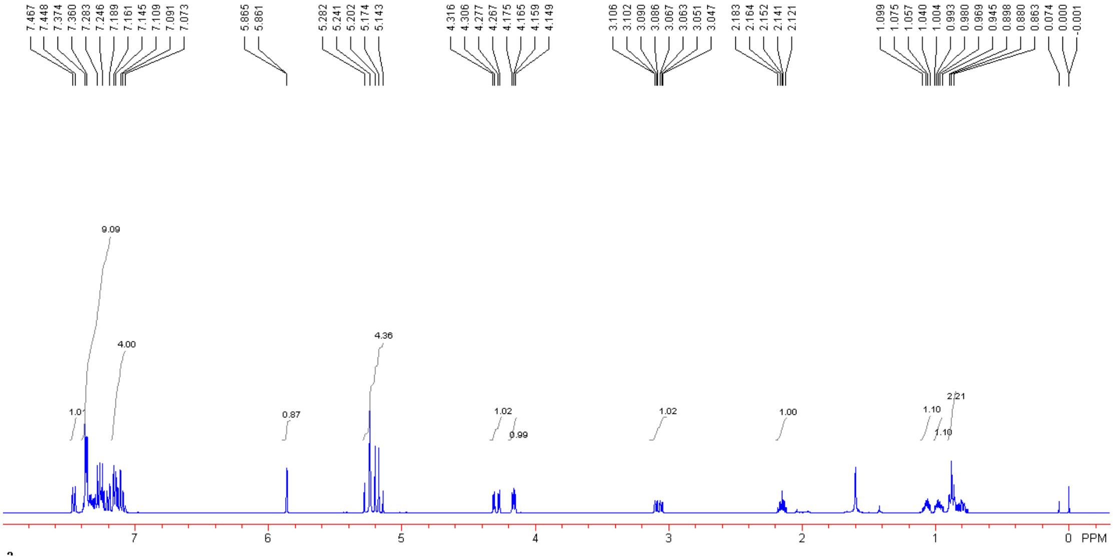

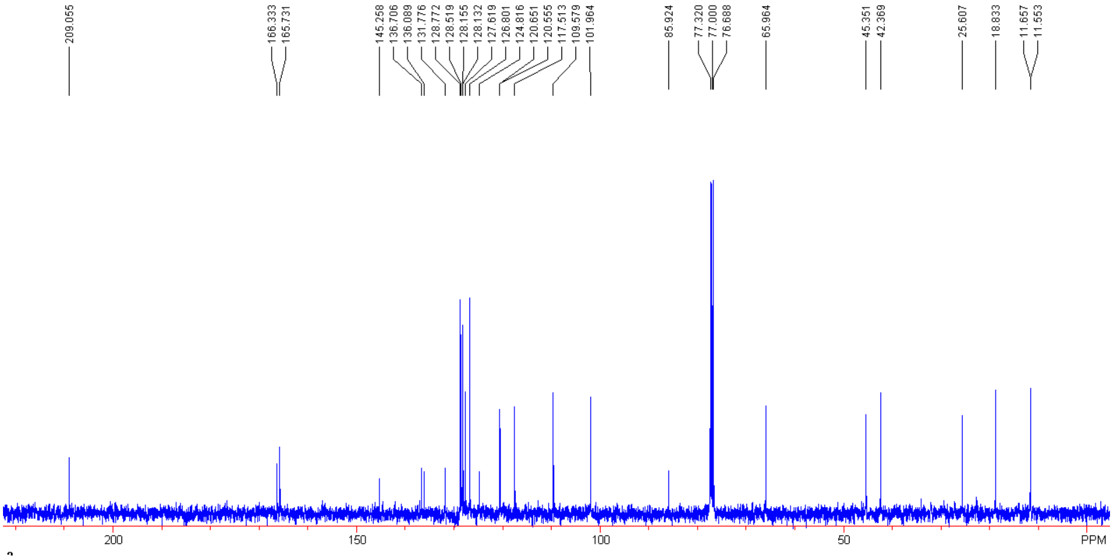
209.055
166.333
165.731
145.258
136.706
136.089
131.776
128.772
128.519
128.155
128.132
127.619
126.801
124.816
120.651
120.555
117.513
109.579
101.964
85.924
77.320
77.000
76.688
65.964
45.351
42.369
25.607
18.833
11.657
11.553
200
150
100
50
PPM

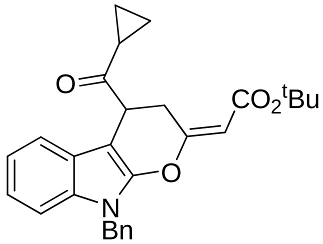

Compound 5v: Yield: 20 mg. 49%. A red solid. Mp: 99-101 °C. IR (neat) ν 2926, 1702, 1645, 1463, 1104, 750 cm^{-1}. ^1H NMR ($CDCl_3$, 400 MHz, TMS) δ 0.81-0.89 (m, 2H), 0.96-1.02 (m, 1H), 1.05-1.11 (m, 1H), 1.50 (s, 9H), 2.11-2.17 (m, 1H), 3.10-3.18 (m, 1H), 4.11-4.18 (m, 2H), 5.24 (s, 2H), 5.75 (d, *J* = 1.2 Hz, 1H), 7.06-7.20 (m, 5H), 7.24-7.30 (m, 3H), 7.41-7.44 (m, 1H). ^{13}C NMR ($CDCl_3$, 100 MHz, TMS) δ 11.5, 11.6, 18.6, 25.5, 28.2, 42.6, 45.3, 80.4, 85.8, 104.2, 109.5, 117.5, 120.4, 120.6, 124.9, 126.8, 127.6, 128.8, 131.8, 136.8, 145.5, 163.9, 165.8, 209.3. HRMS (ESI) Calcd. for $C_{28}H_{30}NO_4$ $(M+H)^+$: 444.2169, Found: 444.2163.

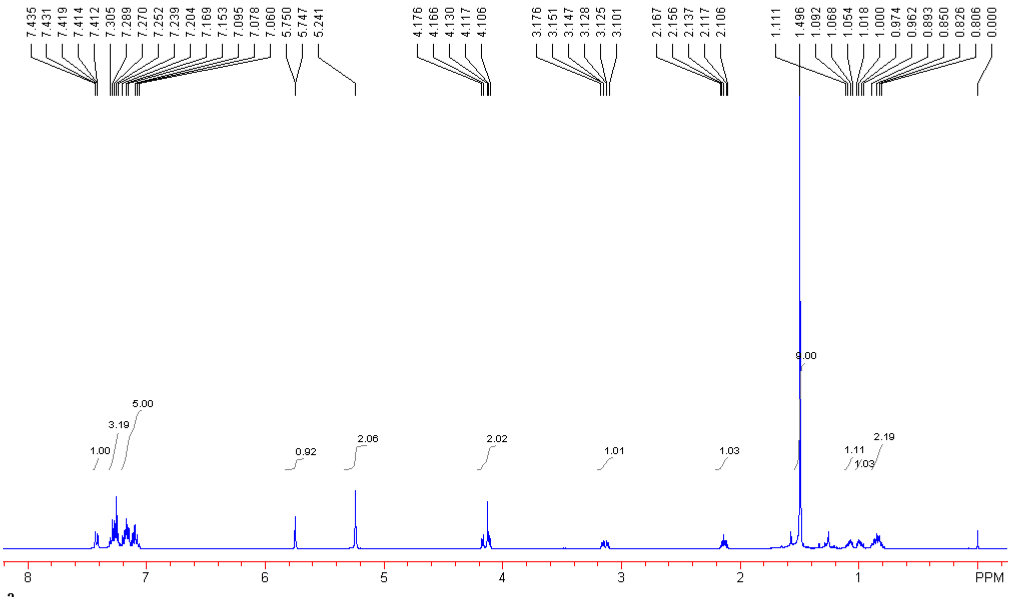

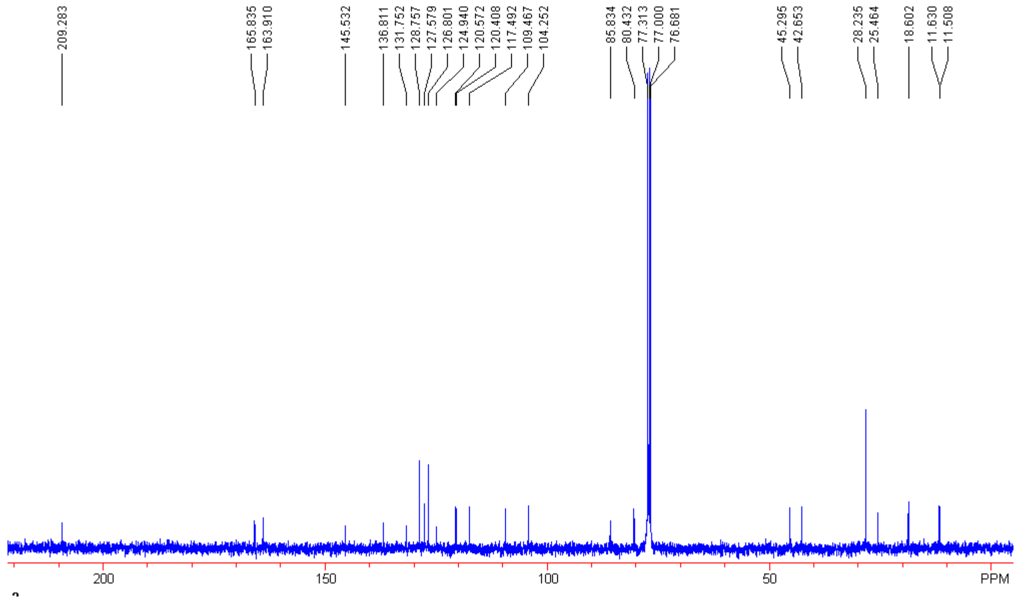
209.283
165.835
163.910
145.532
136.811
131.752
128.757
127.579
126.801
124.940
120.572
120.408
117.492
109.467
104.252
85.834
80.432
77.313
77.000
76.681
45.295
42.653
28.235
25.464
18.602
11.630
11.508
200
150
100
50
PPM

Gas chromatograph (GC) for BnOH

数据文件： C:\CHEM32\1\DATA\SIG10055.D
样品名称： BnOH

===

操作者 :
仪器 : 仪器 1 位置 : 样品瓶 1
进样日期 : 2012-4-17 12:41:25
进样量 : 手动
采集方法 : C:\CHEM32\1\METHODS\DEF_GC.M
最后修改 : 2012-4-17 12:41:24
(调用后修改)
分析方法 : C:\CHEM32\1\METHODS\DEF_GC_OFF.M
最后修改 : 2012-4-18 9:44:24
(调用后修改)

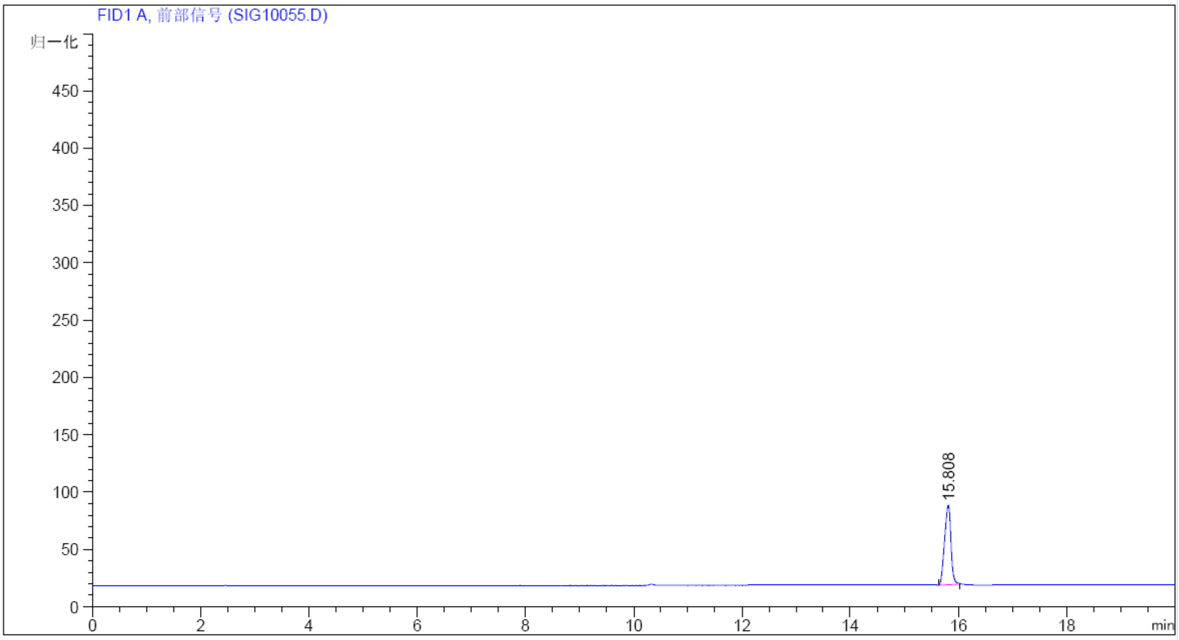

===

面积百分比报告

===

排序 : 信号
乘积因子: : 1.0000
稀释因子: : 1.0000
内标使用乘积因子和稀释因子

信号 1: FID1 A, 前部信号

峰 #	保留时间 [min]	类型	峰宽 [min]	峰面积 [pA*s]	峰高 [pA]	峰面积 %
1	15.808	BB	0.1101	558.41864	69.22991	1.000e2

总量 : 558.41864 69.22991

===

*** 报告结束 ***

Gas chromatograph (GC) for reaction mixture of 1a under the standard conditions

数据文件: C:\CHEM32\1\DATA\SIG10057.D
样品名称: zdh-13-33

===

操作者	:		
仪器	: 仪器 1	位置	: 样品瓶 1
进样日期	: 2012-4-17 14:07:59		
		进样量	: 手动
采集方法	: C:\CHEM32\1\METHODS\DEF_GC.M		
最后修改	: 2012-4-17 14:07:57 (调用后修改)		
分析方法	: C:\CHEM32\1\METHODS\DEF_GC_OFF.M		
最后修改	: 2012-4-18 9:44:24 (调用后修改)		

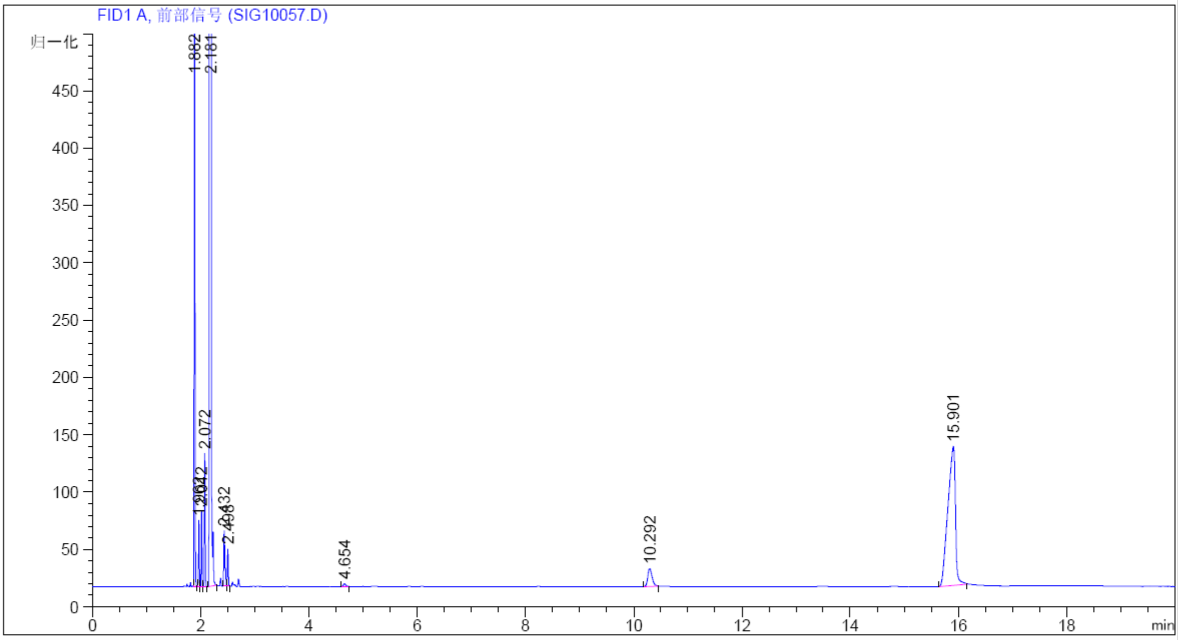

===

面积百分比报告

===

排序 : 信号
乘积因子: : 1.0000
稀释因子: : 1.0000
内标使用乘积因子和稀释因子

信号 1: FID1 A, 前部信号

峰 #	保留时间 [min]	类型	峰宽 [min]	峰面积 [pA*s]	峰高 [pA]	峰面积 %
1	1.882	BB	0.0128	752.05359	913.27466	1.93872
2	1.962	BV	0.0147	56.55251	57.20582	0.14579
3	2.012	VV	0.0152	63.24458	66.04362	0.16304
4	2.072	VB	0.0168	119.23566	115.54932	0.30738
5	2.181	BB S	0.0231	3.64309e4	2.66477e4	93.91537
6	2.432	BV	0.0224	73.03480	48.42099	0.18828
7	2.498	VB	0.0187	38.92102	32.68049	0.10033

数据文件: C:\CHEM32\1\DATA\SIG10057.D
样品名称: zdh-13-33

峰 #	保留时间 [min]	类型	峰宽 [min]	峰面积 [pA*s]	峰高 [pA]	峰面积 %
8	4.654	BB	0.0490	7.22298	2.28022	0.01862
9	10.292	BB	0.0884	87.09077	15.58698	0.22451
10	15.901	BB	0.1322	1162.94495	120.87982	2.99796

总量 : 3.87912e4 2.80196e4

==

*** 报告结束 ***
